# Control of paratuberculosis: who, why and how. A review of 48 countries

**DOI:** 10.1186/s12917-019-1943-4

**Published:** 2019-06-13

**Authors:** Richard Whittington, Karsten Donat, Maarten F. Weber, David Kelton, Søren Saxmose Nielsen, Suzanne Eisenberg, Norma Arrigoni, Ramon Juste, Jose Luis Sáez, Navneet Dhand, Annalisa Santi, Anita Michel, Herman Barkema, Petr Kralik, Polychronis Kostoulas, Lorna Citer, Frank Griffin, Rob Barwell, Maria Aparecida Scatamburlo Moreira, Iva Slana, Heike Koehler, Shoor Vir Singh, Han Sang Yoo, Gilberto Chávez-Gris, Amador Goodridge, Matjaz Ocepek, Joseba Garrido, Karen Stevenson, Mike Collins, Bernardo Alonso, Karina Cirone, Fernando Paolicchi, Lawrence Gavey, Md Tanvir Rahman, Emmanuelle de Marchin, Willem Van Praet, Cathy Bauman, Gilles Fecteau, Shawn McKenna, Miguel Salgado, Jorge Fernández-Silva, Radka Dziedzinska, Gustavo Echeverría, Jaana Seppänen, Virginie Thibault, Vala Fridriksdottir, Abdolah Derakhshandeh, Masoud Haghkhah, Luigi Ruocco, Satoko Kawaji, Eiichi Momotani, Cord Heuer, Solis Norton, Simeon Cadmus, Angelika Agdestein, Annette Kampen, Joanna Szteyn, Jenny Frössling, Ebba Schwan, George Caldow, Sam Strain, Mike Carter, Scott Wells, Musso Munyeme, Robert Wolf, Ratna Gurung, Cristobal Verdugo, Christine Fourichon, Takehisa Yamamoto, Sharada Thapaliya, Elena Di Labio, Monaya Ekgatat, Andres Gil, Alvaro Nuñez Alesandre, José Piaggio, Alejandra Suanes, Jacobus H. de Waard

**Affiliations:** 1 School of Veterinary Science, Faculty of Science, University of Sydney, 425 Werombi Road, Camden, NSW 2570 Australia; 2 Animal Health Service, Thuringian Animal Diseases Fund, 07745 Jena, Germany; 3 Clinic for Obstetrics, Gynecology and Andrology with Veterinary Ambulance, Justus-Liebig-University Giessen, 35392 Giessen, Germany; 4 GD Animal Health, 7400 AA Deventer, The Netherlands; 5 Department of Population Medicine, University of Guelph, Guelph, Ontario N1G 2W1 Canada; 6 Department of Veterinary and Animal Sciences, University of Copenhagen, DK-1870 Frederiksberg C, Denmark; 7 Niedersächsische Tierseuchenkasse, 30169 Hannover, Germany; 8 Istituto Zooprofilattico Sperimentale della Lombardia e dell’Emilia Romagna, 29027 Podenzano, Italy; 9 Servicio Regional de Investigación y Desarrollo Agroalimentario (SERIDA), 33300 Villaviciosa, Asturias Spain; 10 Ministry of Agriculture and Fisheries, Food and Environment, ES-28071 Madrid, Spain; 11 Faculty of Veterinary Science, University of Pretoria, Onderstepoort, 0110 South Africa; 12 Faculty of Veterinary Medicine, University of Calgary, Calgary, Alberta T2N 4N1 Canada; 13 Veterinary Research Institute, 621 00 Brno, Czech Republic; 14 Faculty of Veterinary Medicine, University of Thessaly, 43100 Karditsa, Greece; 15 Animal Health Ireland, Carrick on Shannon, Co. Leitrim, N41 WN27 Republic of Ireland; 16 Disease Research Limited, Invermay Agricultural Centre, Mosgiel, 9092 New Zealand; 17 Animal Health Australia, Turner, ACT 2612 Australia; 18 Veterinary Department, Universidade Federal de Viçosa, Viçosa, Minas Gerais 365700-900 Brazil; 19 Friedrich-Loeffler-Institut, Federal Research Institute of Animal Health, 07743 Jena, Germany; 20 Deparment of Biotechnology, GLA University, Mathura, Uttar Pradesh 281 406 India; 21 Department of Infectious Diseases, College of Veterinary Medicine, Seoul National University, Seoul, 08826 South Korea; 22 Facultad de Medicina Veterinaria y Zootecnia, Universidad Nacional Autonoma de México, 76750 Tequisquiapan, Queretaro, Mexico; 23 Instituto de Investigaciones Científicas y Servicios de Alta Tecnología, City of Knowledge, Panama City, 0843-01103 Panama; 24 National Veterinary Institute, Veterinary Faculty, University of Ljubljana, 1000 Ljubljana, Slovenia; 25 Instituto Vasco de Investigacion y Desarrollo Agrario-NEIKER, 48160 Derio, Bizkaia Spain; 26 Moredun Research Institute, Midlothian, EH26 0PZ UK; 27 School of Veterinary Medicine, University of Wisconsin-Madison, Wisconsin, 53706-1102 USA; 28 DILAB – SENASA, Martínez, 1640 Buenos Aires, Argentina; 29 Instituto Nacional de Tecnologia Agropecuaria, 7620 Balcarce, Argentina; 30 Biosecurity Queensland, Department of Agriculture and Fisheries, Toowoomba, Queensland 4350 Australia; 31 Faculty of Veterinary Science, Bangladesh Agricultural University, Mymensingh, 2202 Bangladesh; 32 Regional Association for Animal Identification and Health, 5590 Ciney, Belgium; 33 Animal Health Care Flanders, 2500 Lier, Belgium; 34 Faculté de Médecine Vétérinaire, University of Montreal, Quebec, J2S 6Z9 Canada; 35 Atlantic Veterinary College, Charlottetown, Prince Edward Island C1A 4P3 Canada; 36 Facultad de Ciencias Veterinarias, Universidad Austral de Chile, 5090000 Valdivia, Chile; 37 Escuela de Medicina Veterinaria, Universidad de Antioquia, Medellín, Antioquia 050034076 Colombia; 38 Instituto de Investigación en Salud Pública y Zoonosis, Universidad Central del Ecuador, 17-03-100 Quito, Ecuador; 39 Finnish Food Authority, Mustialankatu 3, 00790 Helsinki, Finland; 40 ANSES Laboratoire de Ploufragan-Plouzané-Niort and GDS France, CS 28440, 79024 Niort Cedex, France; 41 Institute for Experimental Pathology at Keldur, University of Iceland, IS-112 Reykjavík, Iceland; 42 School of Veterinary Medicine, Shiraz University, Shiraz, 71441-69155 Iran; 43 Ministry of Health, General Directorate of Animal Health and Veterinary Medicines, 00144 Rome, Italy; 44 National Institute of Animal Health, National Agriculture and Food Research Organization, Tsukuba, Ibaraki, 305-0856 Japan; 45 Comparative Medical Research Institute, Tsukuba, Ibaraki 305-0856 Japan; 46 School of Veterinary Sciences, Massey University, Palmerston North, 4441 New Zealand; 47 DeerPRO, Dunedin, 9016 New Zealand; 48 Department of Veterinary Public Health and Preventive Medicine, University of Ibadan, Ibadan, Nigeria; 49 Norwegian Veterinary Institute, N-0106 Oslo, Norway; 50 Faculty of Veterinary Medicine, University of Warmia and Mazury, 10-718 Olsztyn, Poland; 51 National Veterinary Institute, SE-751 89 Uppsala, Sweden; 52 Swedish Farm and Animal Health, 62254 Romakloster, Sweden; 53 Scotland’s Rural College, Edinburgh, EH9 3JG UK; 54 Animal Health and Welfare Northern Ireland, Dungannon Enterprise Centre, Dungannon, BT71 6JT UK; 55 USDA-APHIS-Veterinary Services, Riverdale, MD 20737 USA; 56 College of Veterinary Medicine, University of Minnesota, St. Paul, MN 55108 USA; 57 School of Veterinary Medicine, The University of Zambia, 10101 Lusaka, Zambia; 58 Fachabteilung Gesundheit und Pflegemanagement, 8010 Graz, Austria; 59 National Centre for Animal Health, Serbithang, Bhutan; 60 Oniris – INRA, Department Farm Animal Health and Public Health, 44307 Nantes cedex 3, France; 61 Faculty of Animal Science, Veterinary Science and Fisheries, Agriculture and Forestry University, Rampur, Chitwan Nepal; 62 Federal Food Safety and Veterinary Office, 3003 Bern, Switzerland; 63 National Institute of Animal Health, Chatuchak, Bangkok, 10900 Thailand; 64 Facultad de Veterinaria, Lasplaces 1620, CP 11600 Montevideo, Uruguay; 65 Ministry of Livestock Agriculture and Fisheries, CP 11400 Montevideo, Uruguay; 66 Ministry of Livestock Agriculture and Fisheries of Uruguay, CP 11300 Montevideo, Uruguay; 67 Servicio Autonomo Instituto de Biomedicina, Universidad Central de Venezuela, Caracas, Venezuela

**Keywords:** Paratuberculosis, Control, Review, Prevalence, Cattle, Sheep, Goat, Camelid, Deer, Wildlife

## Abstract

Paratuberculosis, a chronic disease affecting ruminant livestock, is caused by *Mycobacterium avium subsp. paratuberculosis* (MAP). It has direct and indirect economic costs, impacts animal welfare and arouses public health concerns. In a survey of 48 countries we found paratuberculosis to be very common in livestock. In about half the countries more than 20% of herds and flocks were infected with MAP. Most countries had large ruminant populations (millions), several types of farmed ruminants, multiple husbandry systems and tens of thousands of individual farms, creating challenges for disease control. In addition, numerous species of free-living wildlife were infected. Paratuberculosis was notifiable in most countries, but formal control programs were present in only 22 countries. Generally, these were the more highly developed countries with advanced veterinary services. Of the countries without a formal control program for paratuberculosis, 76% were in South and Central America, Asia and Africa while 20% were in Europe. Control programs were justified most commonly on animal health grounds, but protecting market access and public health were other factors. Prevalence reduction was the major objective in most countries, but Norway and Sweden aimed to eradicate the disease, so surveillance and response were their major objectives. Government funding was involved in about two thirds of countries, but operations tended to be funded by farmers and their organizations and not by government alone. The majority of countries (60%) had voluntary control programs. Generally, programs were supported by incentives for joining, financial compensation and/or penalties for non-participation. Performance indicators, structure, leadership, practices and tools used in control programs are also presented. Securing funding for long-term control activities was a widespread problem. Control programs were reported to be successful in 16 (73%) of the 22 countries. Recommendations are made for future control programs, including a primary goal of establishing an international code for paratuberculosis, leading to universal acknowledgment of the principles and methods of control in relation to endemic and transboundary disease. An holistic approach across all ruminant livestock industries and long-term commitment is required for control of paratuberculosis.

**Electronic supplementary material:**

The online version of this article (10.1186/s12917-019-1943-4) contains supplementary material, which is available to authorized users.

## Background

Paratuberculosis is a mycobacterial disease of ruminants caused by *Mycobacterium avium subsp. paratuberculosis* (MAP)*.* It begins as a localised infection that may become systemic and often results in chronic granulomatous enteritis leading eventually to weight loss, (diarrhoea in some species) and death. This syndrome is also known as Johne’s disease. Depending partly on how long it has been present in a herd, it can manifest as isolated clinical cases or persistent outbreaks. The pathogenesis of paratuberculosis is similar to tuberculosis and other mycobacterial diseases, MAP being a specialised intracellular pathogen that triggers a debilitating immunopathological response, sub-clinical infections however being more common [1]. Most species of ruminant livestock are susceptible to paratuberculosis. It is present in many countries and is perceived to be an important disease for reasons including its impacts on the economy, the impact of clinical disease on animal welfare, and public health.

### Why should it be controlled?

#### Economic impacts

Economic losses due to paratuberculosis are a key driver to control MAP. While the economic losses in dairy cattle have been extensively studied, the difficulties in quantifying them have been highlighted in two reviews [2, 3]. Losses to the dairy farmer consist of losses before, during or after culling [4]. Losses before culling may include reduced milk production of variable magnitude [5–10], increased somatic cell counts [7, 8, 10–12], increased incidence of clinical mastitis [10, 13, 14], reduced fertility [2, 15, 16], increased susceptibility to other diseases [17, 18] and costs of testing and treatment [4]. Cattle infected with MAP have higher on-farm mortality and cull rates [19–23], as do veal calves that originated from dairy herds with paratuberculosis [24]. Because MAP infection is associated with a lower body weight [25, 26], the slaughter value of infected cattle is reduced [4, 19, 20, 27, 28]. Costs after culling are the loss of unrealised future income by culling an individual [4, 29, 30].

The total annual economic losses per cow in infected USA dairy herds were estimated at US$21 to US$79 [20, 31–34] while those in infected Australian, Canadian, French, and UK dairy herds were estimated at A$45-A$88 [29, 35], CDN$49 [36, 37], €234 [38] and GBP27 [39], respectively. In ‘average’ Dutch dairy herds (both infected and uninfected), these losses were estimated at up to €67 per cow per year [32]. The effect of the reduction in milk production caused by MAP infection in US dairy herds to the US economy was estimated to be an annual loss of US$200 million ± US$ 160 million [40].

In beef cattle, the losses due to MAP infections depend on the markets targeted by the farmer. Pedigree farmers selling breeder cattle are much more affected than farmers selling all their cattle directly for slaughter or to feedlots [41]. As with dairy cattle, test-positivity in beef cattle was associated with lower cow fertility, weight loss, and lower calf body weight at birth and at 205 days of age [42]. Total annual losses per cow in the first 15–20 years after MAP introduction in suckler herds were estimated to be on average GBP16 in British herds [43] and €40 in French herds [38], whereas in Dutch suckler cow herds (both infected and uninfected) they were €10 (small herds) to €28 (large herds) [44]. Farmers and veterinarians perceived the annual losses per cow in infected suckler cow herds to be around on average US$16 to US$17 [45]. However, most of the potential losses due to MAP infection are hidden or opportunity costs, not out-of-pocket losses [46].

Published estimates of economic losses in other domestic ruminant species are sparse. In an Australian study of 12 sheep flocks, the average annual mortality rate due to paratuberculosis varied between 6.2 and 7.8%, resulting in a decrease of the average gross margin of between 6.4 and 8.5% [47]. However, in some Australian flocks, mortality approached 20% per annum [48]. In addition to mortalities, weight loss in affected sheep is of economic importance [49]. Infected fine wool Merino flocks in New Zealand lost US$1.5 per ewe annually which was about 3-fold as much as in mutton breeds, while a case fatality rate of 1.2–2.7% was the main cost factor (Gautam et al. 2019, in press). The economic losses of paratuberculosis to the British sheep industry were estimated at GBP 0.4 to 32 million per annum, depending on assumptions on the prevalence of infection, the mortality rate and the replacement strategy [50]. A recent economic study showed that MAP infection decreased the profit efficiency from 84 to 64% in Italian dairy sheep and goat farms [51].

In addition to the economic production losses described above, indirect losses may arise from trade restrictions at both the international [52] and national levels. For instance, at the national level, Dutch dairy herds can only deliver milk to their milk processors if they periodically test their herd and cull any test-positive cattle [53]. Furthermore, it is important to realise that production losses caused by MAP not only impact infected farms, but also have a negative economic impact on consumers [54]. Despite the magnitude of these economic losses to both infected farms and consumers, probably the most important economic driver to control MAP infection in livestock is uncertainty about the potential involvement of MAP in human disease [55, 56]. Even in the absence of a proven link between MAP and human disease, the economic consequences of reduced milk demand could be large if consumers’ perception of risk is large or if risk-mitigation strategies were perceived to be ineffective [57].

##### Potential public health implications

There are myriad publications concerning the link between MAP and diseases in humans. The authors of a series of review articles with rigorous methodologies concluded that sources of human exposure existed [58, 59], and “while the zoonotic potential of *M. paratuberculosis* cannot be ignored, due to important knowledge gaps in understanding its role and importance in the development or progression of human disease, its impact on public health cannot yet be quantified or described” [60] . Thus, MAP can infect humans but the infection may or may not cause Crohn’s disease, the condition with which MAP is most often linked. Consequently, the reviewers concluded that steps beyond the programs that the dairy and ruminant industries had already developed for animal health and economic reasons could not be justified by public health authorities [60]. In other words, there was reliance on animal health authorities to take the lead in managing the risk, i.e. to reduce the exposure of humans to MAP by controlling paratuberculosis in livestock.

##### Trade of livestock

A major conundrum exists regarding trade of livestock and their products. Whereas members of the World Trade Organization (WTO) are required to take an “appropriate”, scientifically based level of sanitary measures to protect human and animal health, WTO members cannot discriminate between members where similar conditions prevail, e.g. a WTO member cannot require freedom of MAP in traded livestock if they themselves do not carry out activities to document such freedom [52]. The World Organization for Animal Health (OIE), whose standards are recognised by WTO, offers no guidance on paratuberculosis [61]. The International Association for Paratuberculosis therefore provided guidelines for movement of livestock, according to the rules laid down in the Sanitary and Phytosanitary (SPS) Agreement of WTO [52]. Furthermore, the parliament of the European Union (EU) in 2016 decided to list diseases according to specific criteria through the EU Animal Health Law [62]. This law should be implemented in all member states of the EU in 2021 with specific rules for each disease.

The European Commission in 2016 asked the European Food Safety Authority (EFSA) for advice regarding paratuberculosis, and EFSA proposed that paratuberculosis complied with the criteria to be categorized with regards to movement control (Category D) and surveillance (Category E) to avoid spread of MAP [56]. After the exchange of views between European Commission and Member States in the Standing Committee on Plants, Animals, Food and Feed, paratuberculosis was finally included only under Category E (surveillance) for the listed animal species *Bison spp., Bos spp., Bubalus spp., Ovis spp., Capra spp., Camelidae* and *Cervidae.* Category D and other categories were disregarded, mainly due to the lack of individual animal sensitivity of current diagnostic tests and the difficulties associated with granting paratuberculosis free status to herds, areas and countries. Somewhat paradoxically the same tests are being used within countries for disease control purposes (see below).

#### Epidemiology and pathogenesis of paratuberculosis

MAP infections occur. in domestic and wild ruminants throughout the world, with some monogastric species also having been reported as infected [56, 63]. Apparent spillover or endemicity has been described in wildlife in some countries, with economic consequences for farmed wildlife and unknown consequences for conservation of free-ranging wildlife, endangered species or those in captive breeding programs [64, 65]. Transmission of MAP is often insidious [66]. No country can be considered without risk of introduction of the disease. Thus, freedom from disease is challenging to document and requires extensive surveillance and reporting. Sweden claims to be free of paratuberculosis in cattle since 2008, and Norway has had no known cases of paratuberculosis since 2015.

The age-profile within herds and flocks is a factor influencing the occurrence of clinical disease because of the long incubation period. While the onset of clinical paratuberculosis is usually at adult ages, many individuals become infected during the first weeks of life when they are exposed to MAP from older animals shedding the bacterium in their faeces. Infection at older ages is also possible [67, 68]. MAP is mainly transmitted directly by the faecal-oral route, including via faecal contamination of the udder or pasture, but the formation of aerosols might take bacteria to environmental niches from which it could spread to naive cattle [69]. Other routes like in utero are not negligible [70]. Lower environmental exposures may explain why the disease is much less prevalent in extensively raised beef cattle than in intensively raised dairy cattle. Commonly in beef cattle herds the animals are slaughtered before they reach the more advanced stages of infection where heavy MAP shedding occurs; thus there is less opportunity for environmental contamination. However, this does not explain the widespread disease in small ruminants raised in extensive conditions like those in Australia or Spain. MAP is resistant to acidic soils and low temperatures, including freezing, but it seems to be less resistant in hot and dry climates. Protected from sunlight, MAP survived for up to 55 weeks in a dry fully shaded environment [71]. If vegetation is removed and shading is limited, survival is reduced to a few weeks [72].

Within species, some breeds of livestock are more likely than others to develop clinical paratuberculosis in a given time frame [73]. Further, associations between susceptibility to paratuberculosis and specific genetic markers have been demonstrated [74], notwithstanding the problems of inconsistent phenotypic classification [75, 76]. Based on histopathological evidence, latent forms of the disease, probably representing varying degrees of resistance, account for the majority of all infections [77].

After oral exposure, the first site of MAP colonization is the ileal and jejunal Peyer’s patch, an organized lymphoid tissue in the intestinal mucosa and submucosa, from where it spreads to the mucosal lamina propria [78]. MAP shows a clear tropism for this site, exiting the host via the intestinal lumen, thus explaining the main route of spread in faeces [79]. The amount of mycobacteria shed in faeces is most relevant for transmission of the infection through environmental contamination.

#### Practices and tools for the control of paratuberculosis

The introduction of MAP into a herd is often recognized only after spread has occurred [66]. Control of paratuberculosis then depends on population-level measures such as the culling of animals that are shedding MAP, applying hygienic measures aimed at reducing infection of neonatal/young stock, and vaccination. Epidemiological models in dairy cattle suggest that test and cull or actions targeting infection routes are effective strategies to decrease MAP prevalence [80–82]. These results are reflected in independent data from other herds [83, 84]. Another risk factor that can be managed is the introduction of livestock onto the farm [83, 85]. However, based on studies conducted in several countries the compliance of farmers with recommendations on control of paratuberculosis can be low [86, 87].

MAP control programs can have different goals ranging from reduction of clinical cases and/or MAP prevalence in a herd, which has been shown to be feasible in general [84, 88, 89], to eradication of MAP from the herd, which can be achieved only in some herds [90, 91]. Stamping out can be mandated when the prevalence is low to zero.

Depending on the goal, several control strategies apply. One is the identification and elimination of clinically diseased and/or subclinically infected animals (‘test-and-cull’) [92]. However, as a stand-alone measure this will not eradicate paratuberculosis in the long term [93–95]. A second strategy applicable to all species is the prevention of MAP transmission within the herd by enhancing on-farm biosecurity especially during rearing of young stock. The main intervention strategy in dairy cattle operations is preventing calves from contacting the faeces of adult cows [96]. This avoids faecal-oral transmission and in practice is achieved by improving calving area hygiene and management of colostrum/milk feeding. A process called “snatching”, that involves removing goat kids at birth and separating them from their dams, was successfully used to sanitize goat herds in previously endemic areas in Norway [97], and is used in the Netherlands. Basic preventive measures concentrate on improving hygiene measures in young stock raising [98] but should be extended to all management areas to increase success [32, 88]. Small-scale field trials as well as simulation studies have shown that the most effective control strategy consists of a combination of ‘test-and-cull’ and increasing on-farm biosecurity [94, 99, 100]. The results of a modelling study indicated that improving calf hygiene and ‘test-and-cull’ were both necessary to stabilize the herd status, but reduced calf exposure was confirmed to be the most influential measure, followed by testing frequency and the proportion of infected animals that were detected and subsequently culled [101]. Culling the progeny of known infected cows may also be considered as part of the control strategy as there is a significant rate of in utero infection with MAP [70]. Considering the finite environmental survival of MAP, pasture and grazing management can be utilised to reduce the exposure of extensively grazed livestock [71, 72].

As a further tool, biosecurity measures have to be in place to control the transmission between herds and to protect MAP-negative herds from new infection or reintroduction of MAP. The purchase of sub-clinically infected animals is considered to be the main factor for between-herd transmission [102]. Therefore, MAP-herd status certification that reflects the risk of MAP transmission by animals originating from that herd is essential [103, 104].

Vaccination of ruminants has been demonstrated in controlled studies to be effective in reducing the clinical incidence of paratuberculosis, delaying the onset of the disease and reducing faecal shedding of MAP, thus reducing the economic losses as well as transmission of MAP [105, 106]. There can be considerable benefit of vaccination relative to the cost [107] but there does not appear to have been a critical review of this topic. Vaccination is not widely used in paratuberculosis control in cattle. This is because of the risk of interference with intradermal testing for bovine tuberculosis [108–110]. Importantly and in contrast, in the Australian, Icelandic and Spanish sheep industries, vaccination has been a key element in control leading to a significant reduction in within-herd prevalence [111, 112].

A very important element in MAP-control is communication to farmers and veterinarians about the importance and the components of the program [113–115] and the need to consider farmers’ attitudes towards the implementation of control measures on their farms [116].

#### Diagnostic tests for paratuberculosis

Diagnostic tests used to detect paratuberculosis are generally imperfect yet most are useful if applied properly when a specific purpose has been identified. Examples of purposes are establishment of populations at low risk of infection, reduction of economic impact in diseased animals, eradication, confirmation of clinical disease, surveillance and prevalence estimation [117]. The purpose defines the stage of disease that must be detected: “infected” (MAP has colonised the animal’s tissues), “infectious” (MAP is being shed in faeces) or “affected by MAP” (the animal is clinically diseased) [118]. Detection should result in a decision [119] which could, for example, be culling an animal to reduce the transmission of MAP or to reduce its suffering. The relevant stage of the disease to target and the applicable diagnostic test are linked. Case definitions and terminology are very important for consistent application of tests and interpretation of test results [76].

A wide range of tests exist. In general, they can be classified as tests for MAP, or tests for the host immune response against MAP. Bacterial culture or polymerase chain reaction (PCR) used for faecal samples are direct measures of bacterial shedding of the individual, whereas enzyme-linked immunosorbent assay (ELISA) used for serum or milk, and agar gel immunodiffusion (AGID) and complement fixation test (CFT) are measures of humoral immune responses, also of the individual. Tests for the humoral immune response are applicable in more advanced stages of the pathogenesis associated with major bacterial shedding, reduced milk yield and reduced weight at slaughter [27, 120–122]. Immune responses detected by such tests are not always specific for MAP infection [123], and similarly faecal tests may detect MAP that has merely been ingested and then passively shed, i.e. without true infection [124].

Faecal culture and faecal PCR are widely used tests for MAP, while ELISA is commonly used to detect an antibody response against MAP. AGID and CFT are less commonly used and less sensitive compared to ELISA [120, 125]. The choice between tests can be related to costs and logistics [100, 126]. Even though the sensitivity of serum ELISA is considerably lower than the sensitivity of faecal culture or faecal PCR, its sensitivity is fairly high in cattle in advanced stages of infection [118, 127] and higher S/P readings are associated with a higher proportion of cattle shedding MAP in their faeces [128]. A definitive diagnosis of MAP infection in the individual may be made on gross and histopathological examination, but even this test is not perfect and may require a high number of tissues to rule out infection [129].

The accuracy of diagnosis of the individual can be increased significantly if the results for the individual are used in context of historical results of the herd or flock of origin [130, 131]. These results can include individual diagnostics as mentioned above, or herd- or flock-level diagnostics such as culture or PCR on environmental samples [132] [133], or use of pooling [90, 126, 134, 135]. Herd- and flock level diagnostics can also be used to inform risk mitigation strategies for the herd or flock. If there are no historical results, different types of tests can be used in parallel or in series to increase the sensitivity or specificity, respectively.

The Ziehl-Neelsen (ZN) stain of faecal or tissue smears may serve as a fast diagnostic tool to support clinical diagnosis, but serum ELISA is superior for this purpose [127, 136]. Skin testing to detect cell-mediated immune responses is also an option [137, 138], but its use may affect the skin test for *Mycobacterium bovis*. Furthermore, detection of cell-mediated immune responses using the skin test or in vitro tests such as the interferon-gamma release assay may merely reflect exposure to MAP and not be related to infection status. Lastly, bulk tank milk ELISA is also used on occasion, but low within-herd prevalence usually precludes a practical application [139] even though there is statistical evidence of diagnostic value [140, 141]. OIE [117] provides an overview of different tests for different purposes, but a multitude of considerations are needed to establish a useful test-strategy (see e.g. [142]).

#### History of paratuberculosis control programs

Control programs for paratuberculosis have existed since early last century. Their chronology was documented by Benedictus et al. [113]: the first programs were set up in France in the 1920’s followed by the Netherlands in 1942, Great Britain and Norway in the 1960’s, then Cyprus and the United States of America in the 1970s. Iceland had a program to control paratuberculosis in sheep in the 1950s [112]. The Australian program began in the 1990’s [143]. Control programs for paratuberculosis were then implemented in other developed countries, and excellent reviews of these cover the period up until 2012, bringing information together particularly for cattle [144–147]. However, there are other important, susceptible, livestock species and since 2012 new programs have commenced (e.g. Ireland, Italy) while others have been discontinued or substantially modified (e.g. Australia, United States of America). While recent country-specific information may exist [148], in general there is a lack of authoritative information and it is currently impossible to ascertain from any single source what is being done about paratuberculosis in different countries, or the reasons for coordinated action. Furthermore, it is difficult to find information on the global distribution of paratuberculosis. Consequently, animal health authorities in many countries are not in a good position to make recommendations to their own governments or domestic animal industries, and sometimes they are forced to respond quickly to changing circumstances with incomplete information.

### Scope of this study

The aims of this study were to assess the existence and nature of control programs for paratuberculosis for the period 2012–2018 in countries for which there is reliable information. Further aims were to assess the rationale for control programs, outcomes of past and current control programs, and to make general recommendations for future control programs.

## Methods

### Participants

Collaborators in different countries were identified using chain referral sampling commencing in November 2017 with an invitation to members of the governing board of the International Association for Paratuberculosis (IAP), a not for profit expert body with aims to promote knowledge about paratuberculosis (http://www.paratuberculosis.net), to join a project team. Governing board members are elected by general members of the IAP in countries with multiple IAP members and hence were likely to have strong interest in paratuberculosis control and a network of expert contacts. Through a process of sequential referral from the board members, and subsequently from those whom they nominated, the senior author progressively invited experts to participate as collaborators. Criteria for nomination included: knowledge of, or access to, detailed information about paratuberculosis and national and/or regional control programs in cattle, sheep, goats, camelids, deer, wildlife or zoological collections; approval from employer to participate (if applicable); and willingness to work to a tight schedule in 2018. Those who agreed were sent additional information and asked to comment on the scope of the study and to complete a detailed on-line questionnaire, which was designed to collect information in a consistent manner. Participants (*n* = 27) who were delegates to the 14th International Colloquium on Paratuberculosis, Riviera Maya, Mexico met face to face on June 6, 2018 to discuss the project, review the results of preliminary analysis and recommend topics for further exploration in this paper. The outcomes were circulated to all participants for comment to inform the composition of this report.

### Questionnaire

A draft questionnaire was developed using an on-line platform (Survey Monkey, San Mateo, A, USA) and pre-tested on ten volunteer collaborators each of whom had past experience with surveys for paratuberculosis, from ten countries (Australia, Canada, Czech Republic, Denmark, Germany, Ireland, the Netherlands, New Zealand, South Africa and Spain). Their comments were used to improve the questionnaire, which was then made available by an e-mail link to all participants. A copy of the questionnaire is provided as Additional file 1. Data were exported to Excel (Microsoft Corp., Redmond, WA). To enable country-level assessment when more than one questionnaire was completed for a given country (typically where there were separate responses for different regions of a country), data were aggregated as follows. For animals: the largest regional population on the logarithmic scale was selected; numbers of farms were summed; the midpoint of average herd sizes and the overall minima and maxima were used. At country level: the disease was considered notifiable if it was at regional level; the highest prevalence estimate at regional level was selected; Yes was selected if this was a response in any region; the earliest year for all start events and the latest year for end events were selected; all responses to open questions, and comments, from all regions were concatenated; other data in separate fields at regional level were brought to country level.

### Statistical analyses

Summary statistics were calculated, and box-and-whisker plots were created for numeric variables, namely, number of farms and average, minimum and maximum herd sizes. The y-axes of box-plots were log_10_ transformed to improve their interpretability as their distributions were highly right skewed.

Frequency tables and bar charts were created for categorical variables such as animal population sizes, number of farms and herd/individual prevalence estimates for each species. Maps were prepared in Mapline (version July 2018, www.mapline.com).

Source of herd and individual prevalence estimates for each species were classified into (a) objective, if the source was one of the following: apparent or true prevalence based on serology, apparent or true prevalence based on individual or bulk milk ELISA, or apparent or true prevalence based on faecal culture or PCR; (b) subjective, if the source was one of the following: abattoir monitoring, other active surveillance, passive surveillance, or other; or (c) not applicable. Responses to open questions were manually paraphrased using consistent terms developed following assessment of all responses, summarised into groups and tabulated for analysis.

The relationship between herd-level prevalence estimates and log transformed herd size was determined using ordinal logistic regression. Number of farms and herd sizes were compared between countries with and without a paratuberculosis control program using Wilcoxon two sample tests. Statistical analyses were conducted using SAS (© 2002–2012 by SAS Institute Inc., USA), whereas graphs were produced using R Version 3.5.1 [149] and Excel. Proportions were compared using a two-sample two-sided binomial test in Genstat (VSN International).

### Definitions

#### Paratuberculosis

This was broadly defined as the disease (Johne’s disease), which has characteristic pathology and may be clinical, or infection with *M. avium* subsp. *paratuberculosis* (MAP)*.* The terminology used for paratuberculosis case definitions is available elsewhere [76].

#### Control program

The scope for a livestock disease control program in general was an ongoing process of measures intended to interfere with the unrestrained occurrence of the disease based on Thrusfield [150]. The measures must include elements of planning, coordination, documentation and evaluation, and may be conducted locally, regionally, or nationally. Examples of measures included: estimation of prevalence to inform the decision making; surveillance to detect infected herds; control of the infection in infected herds and flocks; measures to prevent introduction of a disease into free populations; certification of herds and flocks as a low-risk source. Objectives may range from preventing an increase in prevalence through to eradication. Excluded from this definition were: work done by an individual veterinarian on cases of disease on a farm; a private herd health program (also known as a private health and productivity scheme) offered by a veterinarian to an individual herd/farm.

## Results

### Countries and geographic regions

**Fig. 1:**
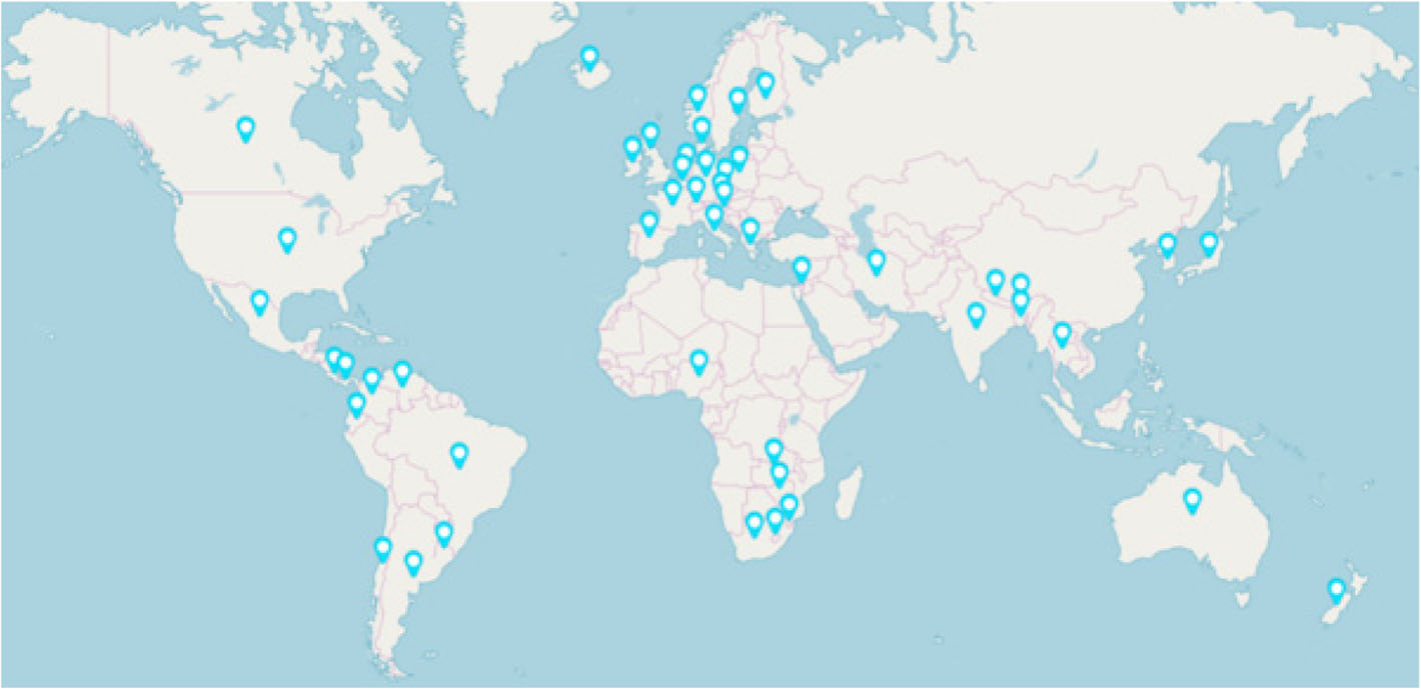
Countries represented in this study

Out of a total of 109 invitees, 83 accepted nominations to collaborate in the project. Over 10 weeks between 26 February and 10 May 2018, 82 collaborators, working individually or as teams within a country, completed the questionnaires. One participant died during the study so that data were obtained for 48 of the 49 countries that came together in the collaboration (Fig. 1): Argentina, Australia, Austria, Bangladesh, Belgium, Bhutan, Brazil, Canada, Chile, Colombia, Costa Rica, Czech Republic, Denmark, Ecuador, Finland, France, Germany, Greece, Iceland, India, Iran, Israel, Italy, Japan, South Korea, Lesotho, Mexico, Nepal, the Netherlands, New Zealand, Nigeria, Norway, Panama, Poland, Ireland, Slovenia, South Africa, Spain, Swaziland, Sweden, Switzerland, Thailand, United Kingdom, United States of America, Uruguay, Venezuela, Zambia, and Zimbabwe. There were six countries from Africa, 19 from Europe, two from the Middle East, seven from Asia, two from Australasia, three from North America, two from Central America and seven from South America. Information was not available from Russia and Central Asia as experts were either not identified or did not respond to invitations following nomination. These countries represented a range of socioeconomic levels. Measured in terms of the United Nations Development Program (http://www.undp.org/content/undp/en/home.html) index ranking, they ranged from 1 (most developed) to 160 (least developed) out of 188 countries. Median ranking was 28.5 and the third quartile was 87.5, indicating that the participating countries were skewed towards those of higher socioeconomic status. More than one questionnaire was completed for the following countries to enable region-specific information to be collated: Belgium (Wallonia, and whole country), Canada (Alberta, Atlantic Provinces, Ontario, and Québec), France (western France and whole country), Germany (Lower Saxony, Thuringia, and whole country), Spain (País Vasco, Castilla y Leon, Comunidad Valenciana, Galicia, and Navarra) and the United Kingdom (Great Britain, and Northern Ireland).

Within the broad geographic regions, the special interests of the collaborators included field aspects of disease control, laboratory science, management and coordination of disease control, communications and education, research and other skills related to disease control including public health. Collaborators from all 48 countries had at least one special interest or skill area and those from 38 countries had special interests in two or more of these categories.

### Animal populations at risk

All but one of 48 countries had at least two of the major types of farmed ruminants (dairy cattle, beef cattle, sheep, goats, camelids and deer), and 38 countries (79%) had at least three (Table 1). Considering also other farmed ruminants (for example buffalo, bison), all 48 countries had at least three types or species and two thirds of the countries (65%) had six or seven types/species.

**Table 1:** Distribution of farmed ruminant types and species among 48 countries

No. types of ruminants	No. countries	
	Dairy cattle, beef cattle, sheep, goats, camelids or deer	All types of farmed ruminants
1	1	–
2	9	–
3	7	1
4	3	9
5	15	7
6	13	17
7	–	14
Total	48	48

About 70% of countries had more than 1 million cattle, sheep or goats and 20% of them had more than 10 million. Beef cattle and sheep were the most numerous of the livestock types; ten countries had > 10 million beef cattle while eight had more than 10 million sheep (Table 2). Goats were the second most numerous type of livestock; five countries had > 10 million. Three countries had > 10 million dairy cattle whereas 20 countries had between 1 and 10 million. Dairy cattle, sheep and goats were present in all 48 countries, while beef cattle were not farmed in three countries (Bhutan, India, Nepal). Camelids and deer were the ruminants that were least often farmed, being reported in populations > 1000 from 12 countries and 15 countries, respectively. Country level data are provided in Additional file 2: Table S1.

**Table 2:** Size of populations of each of the livestock types among 48 countries. Data are the number of countries

Population size	Dairy cattle	Beef cattle	Sheep	Goats	Camelids	Deer-farmed	Other
< 1000			1	2	9	5	2
1000 to 10,000	1	1	1	8	6	8	3
10,000 to 100,000	2	2	7	14	1	5	2
100,000 to 1 million	20	13	15	7	7	2	6
1 million to 10 million	20	18	16	12			2
> 10 million	3	10	8	5			2
Unknown	2	1			9	9	5
Not applicable		3			16	19	26
Total	48	48	48	48	48	48	48

Median number of farms per country was highest for beef cattle (108,723), followed by sheep (36,313) and dairy cattle (21,090), whereas median herd sizes were highest for dairy cattle (108), sheep (68) and beef (50). Box-and-whisker plots for the numbers of farms and herd sizes are presented in Additional file 3: Figure S1 to S4). These data are addressed further in the context of control programs for paratuberculosis in the section below.

### Notifiability of paratuberculosis

Paratuberculosis was notifiable to the relevant authority in at least one of the farmed species in 35 (73%) of 48 countries, but the obligation to notify depended on the species affected (Table 3). Not all species were present in each country. It was notifiable regardless of species in Australia, Finland, Norway and Sweden and in all of the farmed species present in 32 countries. However, in eight countries (Argentina, Austria, Italy, Mexico, Slovenia, South Africa, United Kingdom, Venezuela) notification was not required for some species that were present, typically camelids and deer. In dairy cattle, it was notifiable in 35 of 48 (73%) countries. In beef cattle it was notifiable in 33 (73%) of the 45 countries in which this type of ruminant was farmed, in sheep and goats in 28 (64%) of 44 countries, in camelids in 12 of 28 (43%) countries and in farmed deer in 15 (50%) of 30 countries. It was notifiable in buffalo in Colombia, Brazil, Italy, Japan and Switzerland and in bison in Canada and Switzerland.

**Table 3:** Notifiability of paratuberculosis in each type of ruminant in 48 countries

Species/type	No. of countries			% countries in which species is applicable and paratuberculosis is notifiable
	Notifiable	Not notifiable	Not applicable	
Dairy cattle	35	13		72.9
Beef cattle	33	12	3	73.3
Sheep	28	16	4	63.6
Goats	28	16	4	63.6
Camelids	12	16	20	42.9
Deer-farmed	15	15	18	50.0
Other	10	11	27	47.6

Paratuberculosis was not notifiable in any species in 13 (27%) of 48 countries which were not clustered in any particular geographic region: Belgium, Czech Republic, Denmark, Ecuador, France, Greece, India, Iran, the Netherlands, New Zealand, Nigeria, United States of America, and Uruguay (see Additional file 2: Table S2).

#### Under-reporting

Paratuberculosis was believed to be under-reported in 26 (74%) of the 35 countries in which it was notifiable, regardless of the geographic zone. The reasons given for under-reporting in decreasing order of frequency included lack of knowledge (farmer or veterinarian) or awareness of the clinical signs of disease (eight countries), reluctance to report due to farmer concerns about the consequences of quarantine or other action (5), lack of surveillance (4), notification only of clinical cases (3), poor diagnostic tests (2), lack of concern about paratuberculosis from government (2), lack of application of national legislation (1), government not notifying OIE (1), listing too recent for notification (1), deliberate choice of tests, the results of which are not notifiable (1), farmer culling of cases to avoid detection (1), and farmer fear of the stigma of identification as a positive herd (1). There are also procedural causes of under-reporting, such as testing animals of relatively young age; this was cited by Italy where the productive life of dairy cows averages only 2.6 lactations.

### Prevalence of paratuberculosis

#### Herd-level prevalence

Notwithstanding missing information or unknown situations in some countries and even though a distinction could not be made between apparent and true prevalence (Tables 4 and 5), paratuberculosis was relatively common. Based on objective laboratory test data, herd-level prevalence < 1%, which may be consistent with definitions of freedom, was a feature in very few countries, for example 2 of 31 countries with prevalence data for dairy cattle and 2 of 15 countries with prevalence data for sheep (Table 4). But in 58% of the 31 countries where there were prevalence estimates, > 20% of cattle, sheep or goat herds were infected. Similar proportions were observed where prevalence had been estimated from non-laboratory data (Table 4). Herd-level prevalence by country for each type of ruminant is presented in Additional file 2: Table S3.

**Table 4:** Herd-level paratuberculosis prevalence^a^ estimates in countries where laboratory testing had (or had not) been conducted. Data are the number of countries at each prevalence level

Herd-level prevalence (%)^a^	Dairy cattle	Beef cattle	Sheep	Goats	Camelids	Deer-farmed
< 1	2 (1)	3 (3)	2 (1)	4 (2)	3 (1)	1 (1)
1–10	6 (2)	4	4 (2)	1 (1)		1
10–20	3	4	1	1	1	
20–40	3 (1)	3 (1)	2	2		
> 40	13	1 (1)	2 (1)	4		(1)
Total	27 (4)	15 (5)	11 (4)	12 (3)	4 (1)	2 (2)

^a^The definition of prevalence and the way it was measured may have differed between countries and between species within countries

**Table 5:** Animal-level (within infected herds) prevalence^a^ estimates in countries where laboratory testing had (or had not) been conducted. Data are the number of countries at each prevalence level

Within-herd prevalence (%)	Dairy cattle	Beef cattle	Sheep	Goats	Camelids	Deer-farmed
< 1	1	3 (1)	1 (1)	2	1 (1)	1 (1)
1–5	5	6 (2)	2	2		
5–10	10	5	2	2 (1)		1
10–15	7 (1)		2 (1)	1		
> 15	3	2	2	3		
Total	26 (1)	16 (3)	9 (2)	10 (1)	1 (1)	2 (1)

^a^The definition of prevalence and the way it was measured may have differed between countries and between species within countries

For dairy cattle there was a significant association between herd size and herd-level prevalence of paratuberculosis (*P* = 0.001); for each one log increase in herd size the odds of a country having a higher category of prevalence increased by 9.7 (95% confidence limits 1.9–48.8). There were many missing data for either herd size or prevalence (20 countries had missing values for dairy cattle compared to 30 for beef cattle, 35 for sheep, 35 for goats and 45 for farmed deer; camelids had very few observations).

#### Within-herd prevalence

Based on objective laboratory test data, within-herd prevalence in infected herds of < 1% was a feature in very few countries, for example 1 of 32 countries with dairy cattle and 3 of 21 countries with beef cattle (Table 5). But within-herd prevalence > 10% for most species was common and > 15% was observed in at least 10% of countries. Similar proportions were observed where prevalence had been estimated from non-laboratory data (Table 5). Within-herd prevalence was often unknown (in 12 countries with dairy cattle, 18 with beef cattle, 26 with sheep and 27 with goats). Within-herd prevalence by country for each type of ruminant is presented in Additional file 2: Table S4.

#### Under-estimation of prevalence

Prevalence was believed to be underestimated in 29 (60%) of 48 countries regardless of the geographic zone. Where reasons were given, in decreasing order of frequency they included: the type of tests used underestimate true prevalence (14 countries), lack of surveillance (12), lack of knowledge (farmer or veterinary) or awareness of the clinical signs of disease (2), reluctance to report due to farmer concerns about the consequences of quarantine or other action (1) and lack of concern about paratuberculosis from government (1).

#### Free-living ruminants and wildlife

Wildlife were known to have paratuberculosis in 18 (38%) of 48 countries which were not clustered in any particular geographic zone, but the situation was unknown in 26 countries (54%). Data were not available to separate infection with MAP from disease (pathology associated with MAP infection) in these hosts. Often the hosts were known to be in contact with farmed livestock. The list of species affected was extensive and included omnivorous, herbivorous and carnivorous mammals, including herbivorous marsupials, and even some birds as shown in Additional file 2: Table S5.

### Control programs for paratuberculosis

**Fig. 2:**
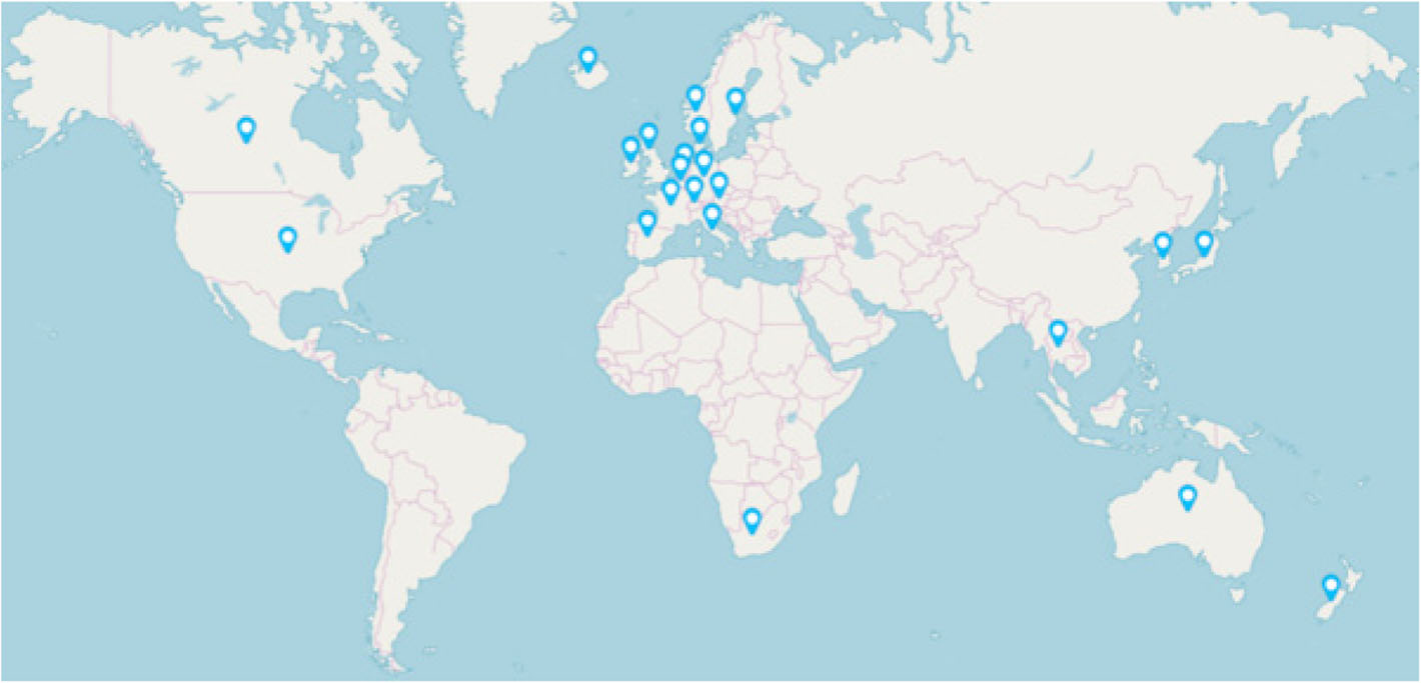
Countries represented in this study that had a control program for paratuberculosis between 2012 and 2018

A total of 22 (46%) of 48 countries had a control program for paratuberculosis in the period 2012 to 2018, i.e. one that commenced, was operating or ended in this period (Fig. 2) (Additional file 2: Table S6). Three of these countries had a prior program that ended before 2012, while 20 of them had a long-term program that will end after 2018. These long-term programs began as early as 1942 in the Netherlands and 1962 in Iceland, compared to as late as 2009 in New Zealand, with a median year of 2000. Seven countries indicated that they will commence a new program in the period 2018–2020, including Chile and Slovenia, neither of which had had control programs before, and five countries which already had a control program in 2012–2018. Only two countries had a control program with a nominated end date, Australia (2018) and Canada (2018). Thus control programs began at different times and changed over time in each country. Major chronological events in the control programs in 22 countries are summarised in Additional file 2: Table S7.

While countries with a control program represented most geographic regions, more than half (14) were in Europe. The majority of countries without a control program for paratuberculosis were in South and Central America, Asia and Africa (19 of 25 countries) and only 5 (20%) were in Europe (Table 6).

**Table 6:** Number of countries with a paratuberculosis control program between 2012 and 2018 in each major geographic region

Geographic region	No. of countries with a paratuberculosis control program between 2012 and 2018	No. countries in this study
Africa	1	6
Asia	3	7
Australasia	2	2
Europe	14	19
Middle East	0	2
North America	2	3
Central America	0	2
South America	0	7
Total	22	48

Using the United Nations Development Program index ranking for 2015, countries without a paratuberculosis control program (median 73, range 19–160) were more disadvantaged than those that had one (median 14, range 1–119).

Number of farms or average herd size did not differ between countries with and without a paratuberculosis control program (Additional file 3: Figure S1-S4). However, minimum herd size for camelids was larger in countries with a control program (*p* = 0.046) and the maximum herd sizes for dairy cattle, beef cattle and sheep were larger for countries with a control program than those without a control program (*p* = 0.04, *p* = 0.03 and *p* = 0.03, respectively).

### Reasons for having a control program for paratuberculosis

All 22 countries with a control program for paratuberculosis cited animal health as a reason for having a control program, whereas 90% cited reducing the economic or production losses (Table 7). Maintaining regional or international trade was a driver for 15 countries (68%) followed by animal welfare in 11 (50%). Public health was cited by eight countries (36%) in Asia (Japan, Korea and Thailand), Europe (Austria, Belgium, Germany and Republic of Ireland) and North America (Canada) as a reason for having a control program. An explicit statement regarding public health existed in the control program documentation or information of five countries (23%) (Austria, Germany, Japan, New Zealand and United Kingdom), two of which did not cite public health as a primary reason for having a control program. The Republic of Ireland made reference to providing “additional reassurance to the marketplace in relation to Ireland’s efforts to control Johne’s disease” in the programme objectives (http://animalhealthireland.ie/?page_id=340) while in the United Kingdom there was a general statement referencing the United Kingdom Food Standards Agency policy towards Johne’s disease and human health, advising that the precautionary principle is adopted (https://www.afbini.gov.uk/articles/johnes-disease).

**Table 7:** Primary reasons for having a control program among 22 countries

Reason	No. of countries	% of countries
Animal health	22	100
Reducing production losses	20	90
Maintaining trade, regional or international	15	68
Animal welfare	11	50
Public health	8	36

### Reasons for not having a control program for paratuberculosis

Of the 26 countries that did not have a program for paratuberculosis, nominated reasons were (Table 8): economic constraints, insufficient animal health resources beyond those already deployed to tackle other priority diseases, a lack of animal health capacity (including infrastructure, operational resources, laboratory diagnostic services) and lack of feasibility due to inadequate control tools such as poor diagnostic tests and poor vaccines. In some countries paratuberculosis was deemed not prevalent at herd or individual animal levels and was not considered to be a problem relative to other animal health issues or it was present but was not considered to affect the animal population. The availability of paratuberculosis vaccine for use by farmers was cited as a reason for not requiring a control program by one country. Other reasons were given by ten countries including: paratuberculosis is not perceived as an immediate problem; there is lack of interest from farmers due to legislative restrictions; bureaucratic ignorance; cessation of funding; lack of resources for collaboration between agencies and regulatory authorities; and lack of cost-benefit except for culling clinical cases.

**Table 8:** Reasons stated for not having a control program for paratuberculosis among 26 countries

Reason	No. countries	% countries
Economic constraints	12	46
Animal health resources are currently deployed to tackle other priority diseases	11	42
Other	10	40
Lack of national/regional animal health capacity, infrastructure or operational resources	8	31
Paratuberculosis is not prevalent at herd or individual animal levels and is not considered to be a problem relative to other animal health issues	8	31
Lack of laboratory diagnostic services	6	23
Paratuberculosis is present but is not considered to affect the animal population	5	19
Lack of feasibility due to inadequate control tools (eg. poor diagnostic tests, poor vaccines)	1	4
Paratuberculosis vaccine is available for use by farmers (there is no coordinated control program)	1	4

### Control programs for other diseases of livestock

Control programs for diseases other than paratuberculosis were present in many countries. Bovine tuberculosis (38 countries), bovine brucellosis (39), rinderpest (9) and foot and mouth disease (32) control programs are examples, while programs for other diseases of livestock were present in 42 countries. Countries without a control program for paratuberculosis were neither more nor less likely to have a control program for one of the other diseases (Additional file 2: Table S8). Countries with a control program for one of the other major livestock diseases were neither more nor less likely to have a control program for paratuberculosis.

### Veterinary services on paratuberculosis for farmers beyond control programs

**Fig. 3:**
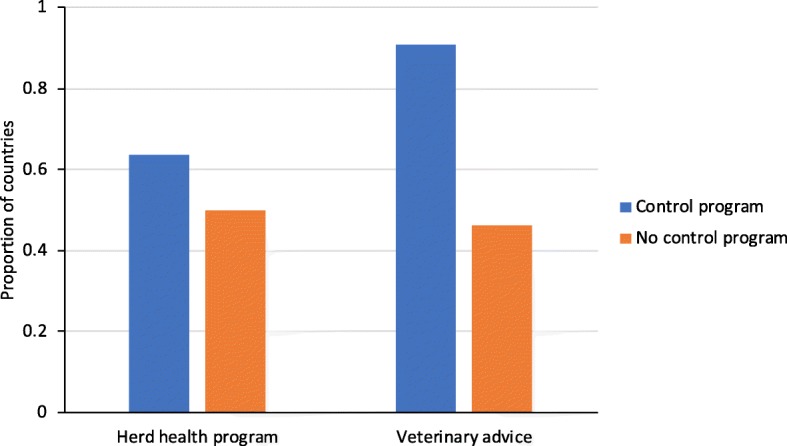
Inclusion of advice on paratuberculosis in herd health programs, and availability of veterinary advice for individual animals in countries with and without a control program for paratuberculosis

Services offered by veterinarians or others for individual farms included herd health programs in 27 of 48 countries and advice on paratuberculosis for individual animals in 32 of 48 countries. While the herd health services were available in a similar proportion of countries with or without paratuberculosis control programs (14/22 and 13/26 countries, respectively; *P* = 0.34), advice to farmers on individual cases of paratuberculosis was available in fewer countries without a control program for paratuberculosis than in countries with a control program for paratuberculosis (12/26 and 20/22, respectively; *P* = 0.001) (Fig. 3).

### Geographic organization of paratuberculosis control programs

It was very difficult to classify control programs strictly as regional or national. For example, in both Australia and Germany there was a national control program agreed through consensus of the regions, each of which had a State Government with jurisdictional control over biosecurity. In this case the programs were implemented and controlled by State jurisdictions, i.e. regionally, and they could differ from region to region in key aspects. There were also differences in the species included in control programs between countries. The geographic organization of paratuberculosis control programs are summarised by country and species in Additional file 2: Table S9.

Eighteen of 22 countries had a single national control program for the relevant species, while five had regional control programs (Belgium, Canada, Germany, France and Spain) that operated in some or most regions of the country, depending on the species. Two countries had more than one national control program for dairy and/or beef cattle while having a single national control program for other types of livestock (the Netherlands, United Kingdom). The regional program activities of regional or national programs were co-ordinated regionally in some countries (Australia, Belgium, Canada, France, Germany, Spain and United Kingdom) and nationally in all other countries. The control programs operating within species in different regions of a country were different from one another in some countries (Australia, Belgium, France, and Germany), but similar or identical to one another in other countries (Iceland, Korea, Spain, Thailand and the United Kingdom). In Canada, they were similar or identical in dairy cattle but different in beef cattle.

### Objectives of control programs

The number of objectives specified by a country and the level of detail in each objective varied between countries and sometimes the methods of achieving an objective was expressed within the objective. This made assessing objectives difficult, and sometimes it was unclear whether objectives expressed were comprehensive statements or whether the descriptions were directly correlated with program activities. Furthermore, objectives may have differed between the different types of livestock in a country. For example, two countries, Australia and the Netherlands, expressed their control program objectives differently for different livestock industries (dairy cattle, beef cattle and small ruminants).

Logically, program objectives and design reflected the stage of paratuberculosis control and initial prevalence. For example, stamping out and then ongoing surveillance was the goal in countries where the disease was very uncommon or absent (such as Sweden and Norway) compared to culling cases in countries where the disease was common. For simplicity, the objectives were aggregated across types of livestock and are presented in Table 9. The most common objective, expressed by 77% of countries with a control program, was to reduce the prevalence of MAP infection, followed by the related objective of reducing the incidence of clinical cases (45% of countries). The methods of doing so while also reducing spread to other herds was expressed quite specifically as a program objective by Spain: to reduce the prevalence of high faecal shedders or faecal shedding. Sweden, which was considered to be free of paratuberculosis, had the same objective but it would apply only if the disease were to be detected through surveillance. Reducing contamination of the human food chain (32% of countries), and the related objective (in two of these countries plus two others) of protecting markets for animal products was the next most common objective, meaning that 9 of 22 countries (41%) had an expressed objective related to product quality. Reducing spread to new farms or regions and certification or assurance of herds to provide a low risk source of stock including replacement stock, were each expressed by 27% of countries. Reducing economic losses and improving animal health, welfare and farm biosecurity were expressed by 18 and 9% of countries, respectively. National or regional eradication of paratuberculosis was an objective in only two countries (9%) (Norway and Sweden) while in three countries (14%) (Germany, the Netherlands and South Africa), eradication at herd level was an objective that could be chosen by a farmer and in one region of Belgium (Wallonia, with 120 herds participating out of 3000) regional eradication was a goal. Research on paratuberculosis and increasing education/awareness were not commonly expressed as objectives (9 and 4.5% of countries, respectively).

**Table 9:** Objectives of paratuberculosis control programs among 22 countries

Objective	No. countries	% countries
Reduce prevalence of MAP (equivalent term = control)	17	77.3
Reduce incidence of clinical cases	10	45.5
Reduce MAP contamination in the human food chain/improve consumer safety	7	31.8
Provide confidence or assurance to, or safeguard markets (including export)	4	18.2
Reduce spread to new farms or regions	6	27.3
Certification of freedom or provide information on low risk herds as a source of replacement stock	6	27.3
Reduce production/economic losses	4	18.2
Risk management: determine risk in a herd; allow trade/marketing with an accredited or specified risk level; reduce within herd spread	4	18.2
Provide individual farmers with tools to manage JD (where manage = prevention or control)	3	13.6
Eradication of MAP from herds that aim to do this	3	13.6
Research including determination of prevalence or incidence	2	9.1
Elimination of high shedders or reduction of faecal shedding	2	9.1
Improve animal health and welfare and farm biosecurity	2	9.1
Country-level eradication (detect, control then eradicate)	2	9.1
Region-level eradication (detect, control then eradicate)	1	4.5
Education and awareness	1	4.5

### Performance indicators for control programs

Thirteen of 22 countries (56%) had performance indicators for their control program (Table 10). The most common goal was an increase in the participation rate of herds in the control program (ten countries) followed by meeting targets in the number of low risk, free or certified herds (5) and reductions in the number of infected animals or clinical cases detected (5). Norway, which had an eradication program, included active surveillance targets and completing farm-level post eradication checks as primary objectives. Sweden, which had advanced beyond this to a surveillance program, used participation rate as the main performance indicator.

**Table 10:** Performance indicators for paratuberculosis control programs in 13 countries

Performance indicator	No. countries
Participation rate of herds	10
Number of low risk, free or certified herds	5
Incidence – the number of infected animals or clinical cases detected	5
Prevalence - within herd	3
Number of infected herds	2
Number of MAP shedders	2
Number of herds in a test and cull plan	1
Animal production data	1
Active surveillance targets met	1
Program review and post eradication checks completed	1

### Review of control programs

The control programs of 13 countries had been subject to review at intervals ranging from annually (three countries) to periodically every two to five years (four countries) and only once in the others. The one-time reviews were conducted as long ago as 2009 in the United States of America and as recently in 2015 in Germany. As a result of review there were substantial changes to the programs in four countries, changes to the objectives in three countries and changes to the operations and methods in nine countries. The programs were not terminated in any country as a result of review.

### Leadership of control programs

Leadership of the control programs among the 22 countries was extremely variable, ranging from seven distinct components in Canada to a simpler structure with just one major nominated leader in 11 countries. In seven of these single-leader programs the leader was the government, while in the remaining four, leadership was provided by a private organization, which in the case of Republic of Ireland was comprised of representatives of government and other sectors. There is likely to be some overlap in these classifications due to representation of government in private organizations and vice versa, and in general the programs of most countries had diverse representation with 11 having more than one type of leadership component. Government was represented in at least 15 countries, farmer organizations in at least 11, a veterinary organization in six, an industry milk organization in four, and an industry meat association in three.

### Funding of programs

The sources of funding for control programs were diverse and varied considerably between countries. Leadership was fully funded by government in 8 of 22 countries and partially funded by government in another eight countries. The next most common source was farmer organizations (eight countries) (see Additional file 2: Table S10). Program operations on the other hand were most likely to be funded wholly or partially by farmers (17 countries) or farmers in conjunction with government (eight countries) compared to government alone (three countries) (see Additional file 2: Table S11).

### Participation and incentives

Participation in the control program was compulsory in at least some regions of nine countries (40%), and voluntary in the other 13 countries. In Australia, participation was compulsory only in some states (Northern Territory, South Australia and Western Australia) while in the rest of the country it was voluntary except for notification of the disease. Compulsory participation was legislated in Austria, Japan, Norway and Switzerland and covered by ordinances in one region of Germany. Components of control that were compulsory varied from country to country according to the objectives of the program. For example, vaccination of sheep was mandatory in Iceland, where it was believed that without the legislation paratuberculosis would be much more widespread. Active surveillance was mandatory in Japan. In South Africa, animals diagnosed with MAP had to be isolated and slaughtered under the supervision of a state official, in-contact animals had to be tested and infected herds/flocks had to be placed under quarantine. Both Norway and Sweden had legislation that made reporting of any suspicion of MAP mandatory, and if MAP is detected (in any species), stamping-out, contact tracing, and eradication is compulsory.

Incentives for participation were identified in 15 countries (68%), comprising ten out of the 13 (77%) with voluntary programs and five out of the nine (56%) with compulsory programs (*p* = 0.29). Incentives for participation included higher prices or higher demand for animals or animal products (four countries), avoidance of penalties such as market access restrictions for non-participation (ten countries), subsidised test costs (five countries) and various other incentives. In some countries, the restrictions were onerous, as were the consequences of diagnosis which could mean exclusion from the market. For instance, in the Netherlands, dairy farmers are required by their milk processors to obtain a preferred herd status in a paratuberculosis control programme [53], meaning that herds have to be tested periodically and any test-positive cattle have to be culled. Incentives and penalties in these countries are listed in Additional file 2: Table S12.

### Financial assistance to farmers

At country level, full or partial financial support, assistance or compensation to farmers for one or more operational aspects of the control program was provided in one or more species between 2012 and 2018 in 12 of the 22 countries (55%). Due to program review processes, this may have begun or ceased during this period, for example in Australia financial support was available to beef farmers until 2016, then ceased. Among the 12 countries in which financial support was provided, the components covered included the cost of testing (11 countries), the cost of culling infected livestock (seven countries), and the value of culled livestock (eight countries). All three components were covered in four countries, two were covered in six countries and one was covered in two countries.

### Practices and tools used in control programs

Control programs for paratuberculosis in the 22 countries were generally multi-component and involved many possible practices and tools (Table 11). Those employed depended on the objectives and hence also on the prevalence of paratuberculosis within the country. The number of tools employed ranged from 2 to 11 among countries with 16 (73%) of the 22 countries employing five or more of the practices and tools listed in Table 11.

**Table 11:** Practices and tools used in control programs for paratuberculosis in 22 countries listed by frequency of inclusion

Tool	No. of countries	% of countries
Cull clinical cases	19	86.4
Hygienic rearing of neonates/juvenile livestock	17	77.3
Farm-level biosecurity to prevent introduction of infection	17	77.3
Test and cull subclinical cases	16	72.7
Environmental and pasture management	14	63.6
Communications program	14	63.6
Herd/flock assurance certification	13	59.1
Research program	11	50.0
Vaccination	7	31.8
Regional biosecurity to prevent introduction of infection	5	22.7
National biosecurity to prevent introduction of infection	4	18.2
Other	4	18.2
Stamping out infected herds/flocks	3	13.6
Individual animal assurance certification	3	13.6

Reflecting the fact that most countries were tackling relatively high burdens of paratuberculosis, practices and tools used by more than half of the countries were the culling of clinical cases (19 countries with a control program, 86%), hygienic rearing of neonates/juvenile livestock (17 countries, 77%), farm level biosecurity planning to prevent introduction of infection (17 countries, 77%), testing and culling of subclinical cases (73%) and environmental and pasture management (64%). In contrast stamping out infected herds/flocks (14%) was uncommon and applied only where the disease was considered to be rare. Some practices and tools would apply regardless of prevalence and included communications and education (64%), and herd/flock assurance or certification (59%). Half of the countries had a research program. Less than one third of the countries employed vaccination (32%), regional biosecurity planning to prevent introduction of infection (23%), national biosecurity planning to prevent introduction of infection (18%) or individual animal assurance certification (14%). Other tools were employed by 18% of countries, including Switzerland, where any animal traffic to and from the infected farm was forbidden, clinical cases as well as their sucking calves/lambs were culled, and the housing was cleaned and disinfected before the animal traffic ban was lifted from the farm. In Sweden, surveillance was the major tool to maintain assurance of national freedom. Vaccination was not permitted in some countries such as Denmark, required a special permit to be used in France, Germany and Spain, and was not applied to all species in countries where it was used, for example sheep and goats only in Australia, Spain and the Netherlands, and sheep only in Iceland and South Africa.

### Implementation of the control program at farm level

Among the 22 countries, the on-farm implementation of the control program was conducted by private veterinarians or para-veterinary staff (e.g., private animal health officers) in eight countries, both private and government veterinarians or para-veterinary staff in seven countries, and by government veterinarians or para-veterinary staff (e.g., animal health officers) in six countries. In the Netherlands, this work was done by a private organization in cooperation with private veterinarians.

### Manuals, protocols and definitions

A publicly available manual describing details of the control program such as the case definitions, rules and procedures of the control program was available for 18 of 22 countries. In Spain, the Government of Galicia provided such information for farmers on-line, but in other regions information would be provided to farmers upon request. There was no manual in South Africa but instead a legislative document. Citations for the information that was available for each country are provided in Additional file 2: Table S13. Definitions for terms used in control programs such as “infected” and “diseased” animals/herds and “control” and “eradication” were often provided in the manuals or other sources of information (77 and 52% of these countries, respectively). Detailed descriptions of the methods of diagnosis/surveillance, control and the rules/regulations associated with control programs were almost always provided in the manuals or other sources of information (96, 95 and 90% of countries where these components were applicable, respectively).

### Diagnostic tests and their purposes in control programs

Diagnostic tests used in control programs in any species in the 22 countries with a control program are summarised in Table 12. Serum ELISA, faecal PCR or culture and pathology were most commonly specified, while agar gel immunodiffusion, complement fixation test (both on blood), intradermal skin test and faecal ZN smear were least often specified. In other species, tests were applied in bison in Canada and Switzerland, water buffalo in Italy and water buffalo in Japan and Switzerland. Other types of tests included testing of environmental faecal samples in six countries (Australia, Canada, France, Germany, New Zealand and United States of America), ELISA on small pools of milk or blood samples in some regions of France, a pooled milk ELISA in one region (Lower Saxony) of Germany, and the serological Enferplex test on goat bulk milk in Norway.

**Table 12:** Types of diagnostic tests used in paratuberculosis control programs in 2012–2018 in each type of livestock. Data are the number of countries among the 22 countries with control programs, sorted by frequency of test

Test	Cattle - dairy	Cattle - beef	Sheep	Goats	Camelids	Deer - farmed	Other	Not used
Serum ELISA	17	17	9	10	1	3		3
Faecal PCR - individual	18	17	13	12	6	9	3	4
Faecal culture - individual	13	13	9	9	6	9	3	5
Pathology	16	15	13	14	8	11	3	6
Faecal PCR - pooled	12	10	5	4	2	3		10
Milk ELISA - individuals	10		1	2				11
Faecal culture - pooled	8	8	5	4	3	3		12
Milk ELISA - bulk milk	6			1				15
Environmental faecal test - culture or PCR	6	2	1	2				16
Complement fixation test	5	5	4	3	2	3	1	17
Faecal ZN smear	4	4	3	3	2	3	2	18
Other	1			1				20
Intradermal skin test	1	1	1	1				21
Serum AGID				1				21

The tests specified by most countries for individual animal diagnosis were serum ELISA, individual faecal PCR or culture and pathology; for individual animal certification/assurance they were individual faecal PCR or culture and serum ELISA; for herd-level screening they were serum ELISA, pooled faecal PCR and individual milk ELISA and for herd-level certification/assurance they were serum ELISA, individual or pooled faecal PCR and individual milk ELISA (Table 13). The complement fixation test (CFT) was used in five countries. In three, CFT was used when required for livestock export (South Africa, Thailand and USA). In Japan the CFT was used not only when requested by importing countries but also for individual diagnosis in small ruminants (sheep and goats) combined with a Johnin skin test. In the Netherlands in 2016 and 2017 the CFT was a minor test, used on only 1% of all submitted sera; the majority of these CFT tests were done in relation to breeding/artificial insemination in parallel to ELISA testing, and for livestock imports or exports.

**Table 13:** Purposes of use of each type of test among control programs in 22 countries. Data are the number of countries, sorted by frequency of test

Test	Individual animal diagnosis	Individual animal certification/ assurance	Herd-level screening	Herd-level certification/ assurance	Other purpose	Test is not used
Serum ELISA	17	2	14	9	2	3
Faecal PCR - individual	18	8	9	8	2	4
Faecal culture - individual	17	3	5	5	1	5
Pathology	15	1	1	2	2	6
Faecal PCR - pooled	3		11	6		10
Milk ELISA - individuals	10		11	6	1	11
Faecal culture - pooled	3		8	4		12
Milk ELISA - bulk milk			6	1	1	15
Environmental faecal test - culture or PCR	1	1	6	4		16
Complement fixation test	4	1	2			17
Faecal ZN smear	4	1	2	1	1	18
Other			2			20
Intradermal skin test	1					21
Serum AGID	1					21

### Communication, extension and education activities

While a defined communications program was present in 14 of 22 countries (Table 11), communication, extension or education and training activities were included within the control programs of 17 (77%) of the 22 countries within the period 2012–2018. The specific target audience for this activity was farmers (nine countries), veterinarians (12), government representatives (2) and stakeholders in general (3) and while a consistent objective was to increase awareness the means of diffusion of information included websites, conferences and seminars, field days and newsletters.

### Research activities

Research was included within control programs in 12 (55%) of 22 countries within the period 2012–2018. The most common research objectives related to improving diagnostic tests and/or diagnostic test validation (ten countries), epidemiological research such as risk factors, transmission dynamics and environmental survival of MAP (4), pathophysiology (3), disease control (3), vaccination (3) and economics (3). Research on prevalence, microbiology, farmer attitudes, food safety, animal genetics and closing general knowledge gaps were included in one or two countries each. Seven developed countries had research programs on paratuberculosis that were conducted independently of control programs (Denmark, Ireland, Japan, the Netherlands, Switzerland, United Kingdom and United States of America). However, in these countries the interaction between research and control programs varied between complete independence (for instance Switzerland) to closely linked (for instance, Ireland). Taken together, research on paratuberculosis that would be available to stakeholders was present in 18 (82%) of 22 countries with control programs.

### Results and success of control programs

The results of control programs operating between 2012 and 2018 were publicly available in 13 (59%) of 22 countries. Examples of the types of outcomes that were reported included incidence of paratuberculosis in sheep (Australia) and deer (New Zealand) based on abattoir surveillance, number of suspect and confirmed cases and farms (Austria, Japan), weekly test prevalence data for dairy cattle (Denmark), numbers of participating dairy (Germany, the Netherlands) or deer (New Zealand) farms, and lists of certified farms (Australia, United Kingdom).

The control program was reported to be successful in 16 (73%) of 22 countries. It was too early to tell in three countries and in three countries outcomes could not be assessed or it was unclear if success had been achieved. Of the countries that reported success, 13 commented that their program objectives had been or were being met while some mentioned specific outcomes such as market access maintained (2), prevented clinical cases (2), or farmers satisfied (2). In addition, four countries with successful outcomes acknowledged specific problems: declining numbers of farms in market assurance programs, the winding back of research and the national control program in general (Australia); herd-level prevalence did not diminish (Belgium); voluntary continuation of the program among producers was much lower than hoped for and funding dried up (Canada); the programme has benefited only a small minority of herds, with most herds not being in any JD control programme, therefore it is unlikely that the programme has had any significant impact on the individual animal or herd-level prevalence in the region (United Kingdom).

### Community and stakeholder support

In more than two thirds of countries with a control program, there was active community stakeholder support from one or more sectors including farmer organizations (82% of 22 countries), government (77%), veterinary organizations (73%) and private veterinarians (73%) (Table 14). Industry organizations for milk and individual farmers were supportive of control programs in the majority of the countries (73 and 64%, respectively). Industry organizations for meat, livestock trading and food processing were supportive in at least 32 to 40% of countries. Note that absence of recorded support from these stakeholder sectors in different countries did not mean that there was opposition, and in some countries it was uncertain whether or not there was support from some sectors (Table 14).

**Table 14:** Sources of stakeholder support by sector for paratuberculosis control programs in 22 countries. Data are the number and % of countries

Sector	Number	%
Farmer organization	18	81.8
Government	17	77.3
Industry organization - milk	16	72.7
Veterinary organization	16	72.7
Private veterinarians	16	72.7
Individual farmers	14	63.6
Industry organization - meat	9	40.9
Industry organization - livestock trading	7	31.8
Food processing industry	7	31.8

There was recorded support for control programs from at least three (range three to nine) of these stakeholder sectors in each of 19 countries (86%) (see Additional file 2: Table S14). Japan (government), South Africa (meat industry) and the United States of America (industry-milk) had active support from only one sector.

Measurement of support from stakeholders was generally informal and subjective. In most countries it depended on feedback obtained during direct engagement or meetings with stakeholders or was evidenced through receipt of ongoing funding. In Iceland, for example, it was generally understood that there was support from all stakeholders and sectors, but it was not formally recorded.

In Australia, a large variance in support among stakeholders was reported; it ranged from actively engaged and supportive through to actively opposed to any sort of program. Similarly, in France support from farmers was reported to be heterogeneous. In Germany (Lower Saxony), there was divergence within and between sectors: the government in one region supported the program by enacting an ordinance and although the milk industry, farmer collectives, veterinary associations, private veterinarians and individual farmers initiated the program and were closely involved in its development, not all farmers and veterinarians were in support of the program. The attitudes among the other stakeholders/beneficiaries towards the control program were diverse. In Switzerland it was recognised that the small percentage of farmers that had animals with clinical signs supported the control program, but there were no data on the opinion of the other farmers. In the United States of America, support between farming sectors differed: there was strong support and lobbying for control program funding by the dairy industry, but less activity from the beef cattle industry.

## Discussion

Analysis of the data in this study revealed that the global prevalence of paratuberculosis is high and therefore should be of great concern: in about half the countries more than 20% of herds and flocks were infected. Prevalence exceeded 40% even in some developed countries. Under reporting was common as was under-estimation of prevalence. Many countries, regardless of their UNDP development index rank, were unaware of the prevalence of paratuberculosis in their herds and flocks (Additional file 2: Table S3). Furthermore, the prevalence of paratuberculosis within infected herds and flocks often is “guesstimated”, and apparent and true prevalence is not often differentiated. Thus many of the data in Additional file 2: Table S4 may be underestimates. For example, in New Zealand, little knowledge exists about the prevalence of infected animals in the infected herds, whereas the annual incidence of clinical cases, which is the frequency of the final stage of the disease, is commonly < 1%. Subclinical infection is much more common, and to illustrate this point the true within-herd prevalence among four dairy herds with few clinical cases in 2010 was actually 6, 7, 15 and 19% [151]. In deer herds in New Zealand, while < 1% of adults died each year (an example of incidence), 45% were MAP culture positive from lymph nodes (an estimate of prevalence) [152].

One of the striking features of paratuberculosis is the variation in prevalence and incidence, regardless of the scale at which it is measured, be it national, regional, between-herd or even within-herd. The variability is due to many factors, including host species and management systems. However, it is important to note that the distribution, prevalence and incidence of paratuberculosis has not yet stabilised in many places, because MAP continues to spread into and within countries, industries and farms, and the transmission dynamics are determined by very long incubation periods [66].

In any country, the resources required to control paratuberculosis must be drawn from a finite pool of resources, and therefore the control effort must be justified and prioritised relative to other needs in animal health and indeed to the broader needs of the industry. Those needs may be determined by a variety of objective factors, including for example the need to reduce economic losses due to the burden of disease, the need to ameliorate animal welfare concerns or the need to meet market access specifications. Clearly the reasons for controlling paratuberculosis that were expressed by many of the countries with a control program addressed these needs. The rules mandated by membership of an economic entity such as the EU, membership of rules-based organizations such as the WTO and OIE, adherence to historical practice, or merely following the lead from nearby countries may also determine what is done or not done. In this study lack of resources of one kind or another was the most common reason among 26 countries for not implementing paratuberculosis control. Simply put, there were other priorities for the available resources. A significant finding was the absence of control measures in relatively under-developed countries; often these had large animal populations and arguably from a human development perspective would benefit the most from an animal health program. Veterinary advice to farmers on individual cases was characteristically missing in these countries too. Countries without paratuberculosis controls of any kind will suffer the greatest impact to human wealth and human health through lost production of animal protein and potential zoonotic impacts.

Paratuberculosis is a notifiable disease in most countries, probably because it is listed by the OIE whose member countries (most countries) have a reporting obligation which requires incidence data. However, lack of guidance from the OIE beyond the reporting requirement allows self-determination with respect to the control of paratuberculosis at country level; there are no OIE guidelines for paratuberculosis [61]. The OIE has existed since 1924 and has 182 member countries distributed on all continents. Its mandate includes elaboration of intergovernmental science-based standards for disease prevention and control. In contrast to paratuberculosis, strict OIE requirements exist for market access and international trade with respect to bovine tuberculosis [153]. Perhaps it was not surprising that we found control programs for bovine tuberculosis to be common while those for paratuberculosis were present in less than half the countries. Beyond bovine tuberculosis there are many other priority diseases, but we found that there was no correlation between engaging in control of these diseases and having a control program for paratuberculosis. This seems to be inconsistent, since paratuberculosis causes substantial production losses, diminished animal welfare and potential zoonotic risk all of which may affect market access. The latter was highlighted recently in Australia through two events, the first being the selective suspension in 2015 of exports of live cattle from farms under investigation of MAP infection in northern Australia, a region thought to be free of the disease, with disruption to a key market in Indonesia. The second event was a trade ban imposed by Japan on importation of live Australian breeder cattle due to detection of paratuberculosis in Australian cattle during their quarantine in Japan in 2016. The former event preceded a review of the national bovine Johne’s disease program in Australia, while the latter event followed this review [154]. Both events caused substantial national industry and government introspection about the best approaches to manage paratuberculosis across diverse geographic regions with different levels of prevalence in different livestock species, and about which certification practices to adopt in the future. The need for more information is likely to be common to many countries and is one justification for the present study.

A potential complication in control of mycobacterial diseases is the existence of wildlife reservoirs. Wildlife play a significant role in the maintenance of *M. bovis* infections in domestic stock but the situation for paratuberculosis is unclear. Although leakage of MAP from farms may lead to wildlife becoming infected, the role of wildlife in the MAP infection cycles of farmed livestock is unproven and we did not explore transmission of MAP between wildlife and farmed ruminants in this study. However, numerous species of free-living wildlife, including monogastric species and carnivores, were reported to be exposed to and infected with MAP (Additional file 2: Table S5). These data are not exhaustive and reviews of MAP in free-living ruminant and non-ruminant wildlife are available [56, 63, 155].

The sizes and complexities of the animal populations at risk of paratuberculosis were extraordinary. About 20% of countries had more than 10 million cattle, sheep or goats, all countries had several types of farmed ruminants and two thirds had six or seven types. Consequently, there were multiple husbandry systems in most countries and commonly there were tens of thousands of individual farms per country. The challenges for disease control in this landscape are enormous as paratuberculosis can spread between livestock species, farms, regions and countries prior to any clinical evidence, while controls need to be implemented at a small scale, i.e. farm level.

One of the limitations of this study is its large scale. For some countries, separate regional data were aggregated from the questionnaire into country-level data (see materials and methods), and this led to loss of some detail. For other countries such as Australia and Colombia, which have significant regional differences in both animal populations and paratuberculosis prevalence, the national data lack granularity. This can only be overcome by separate, detailed national reviews of paratuberculosis. To partially offset the loss of information, summaries are provided for selected countries in Additional file 4.

Just under half (46%) of the countries that participated in this study had a control program for paratuberculosis, the existence of which was justified most commonly on animal health grounds. But more than two thirds of these 22 countries were addressing concerns about market access, and one third had public health aims. The latter driver was overt in a minority of countries, but was probably concealed within the market access driver in the others. For example, in Ireland, the principal concern was to meet the expectations of international customers who operate in food safety sensitive markets, i.e. trade risk management, so public health was an indirect driver. Overall there were great differences between countries with respect to acknowledgment of human exposure to MAP from the livestock sector. For example the precautionary principle was invoked in the control programs of the United Kingdom and Japan, but public health was not overtly mentioned in the control program of Australia [154]. These inconsistencies are remarkable and point to the need for international guidance.

The Wingspread Statement on the Precautionary Principle was promulgated at a meeting in Racine, Wisconsin on 26 Jan 1998 and states “when an activity raises threats of harm to human health or the environment, precautionary measures should be taken even if some cause and effect relationships are not fully established scientifically” [156]. Application of the precautionary principle to MAP exposure in some countries appears to have led to a combination of mandatory government or industry-mandated animal disease control efforts aimed at protecting public health.

The organization, funding and leadership of paratuberculosis control programs in the different countries were quite varied, but there was consistency in objectives because so many countries had a significant burden of paratuberculosis to deal with. As 77% of countries aimed to reduce prevalence, there tended to be emphasis on culling clinical and subclinical cases. In contrast, where paratuberculosis was absent (Sweden), the primary activity was surveillance, and eradication measures would be undertaken there if paratuberculosis was detected. Sweden’s program may be considered a surveillance program. Only 2 of 22 countries (Norway and Sweden) had sufficiently low (or zero) prevalence that they could aim to eradicate the disease.

There was government funding of program leadership in about two thirds of countries, but actual operations tended to be funded by farmers and not by government alone. The majority of countries (60%) had voluntary control programs, and overall programs were supported by incentives for joining, plus financial compensation and/or penalties for non-participation. In Norway and Sweden, compulsory activity to detect (and if found) to eradicate MAP was financially supported by the state. Government support was considered to be an important foundation for surveillance of MAP in Sweden and one reason why prevention, control, and eradication of MAP in Sweden has been successful. Thus, special regulations supported by legislation may be required to control MAP.

There was strong evidence that ongoing review and modification of programs occurred over time and just over half had objective performance criteria. However, herd participation rates may not be a good measure of performance. A feature of successful disease control programs is a decline in the percentage of affected herds over time, while the participation rate may decrease for economic reasons alone. This has been seen in the sheep industry in Australia where some farmers prematurely cease vaccination against paratuberculosis to reduce their production costs. They do this soon after the incidence of clinical cases decreases, whereas successful control requires long term vaccination [111]. Participation rates in certification programs such as the Market Assurance Program in Australia fell over time due to progressive detection of subclinical infections in some herds and market failure to reward participation. These features were perceived negatively by herd owners and were seen as a failure of the national program. However, another perspective is that the rigour of the testing in the Market Assurance Program was effective surveillance, thereby enhancing other objectives of the national program. Arguably the reduction in the annual incidence of clinical cases as a performance indicator, while potentially beneficial in terms of reducing transmission, may lead to a false sense of security, premature cessation of strict control measures and has been confused with reducing prevalence (i.e. the prevalence of subclinical infection may be high in the absence of a high incidence of clinical cases). Furthermore, trends in apparent prevalence can be an inappropriate measure of progress in a test-and-cull scheme, because culling test-positive individuals after a test round will reduce the likelihood of detection using that test at the subsequent test round.

The practices and tools for control of paratuberculosis are well known. Culling of clinical cases, test and cull of subclinical cases, hygienic rearing of young livestock, biosecurity, and environmental pasture management were dominant approaches. However, vaccination was seldom used, despite its potential appeal given the difficulty of other management approaches, the recognised efficacy of vaccines and the scale of the need driven by high prevalence and large population sizes in some countries. Only seven countries with a control program included vaccination. The reasons were not explored in this study, but it is likely that interference of paratuberculosis vaccines with immunological tests for bovine tuberculosis is a major reason for not vaccinating cattle [108, 109]. Paradoxically, the multilateral international trade rules applying to tuberculosis [153] are probably holding back control of paratuberculosis for which there are invisible rules [61]. But this does not explain the lack of widespread use of vaccination in small ruminants in which efficacy has been proven [106] and long term application has been shown to have substantial benefits [111]. In 2000, Benedictus [113] had already concluded that paratuberculosis can be eradicated in sheep and goats by systematic vaccination together with biosecurity measures, while in cattle eradication can be achieved by vaccination followed by culling of faecal culture positive individuals and culling of entire herds if severely affected. In Australia, where bovine tuberculosis has been eradicated, vaccination of infected dairy or beef herds against paratuberculosis is likely to be cost-effective [35].

The application of laboratory tests in control programs has undoubtedly been beneficial and they are instrumental in test and cull programs. Serum ELISA and faecal tests for MAP were the most commonly applied tests regardless of livestock species and they were used for many purposes. Nevertheless, poor diagnostic tests or lack of testing capacity were cited as reasons for not having a control program by seven countries. These reasons can be a constraint to disease control, and so improving diagnostic tests or test validation were research objectives in around half of the countries with a control program.

Essential factors for consideration in disease control programs were listed by Thrusfield [150]. They include adequate knowledge of the disease (cause, maintenance and transmission), diagnostic feasibility and adequate surveillance as well as farmer/public opinion (stakeholder support). In addition, Houe et al. [157] emphasized the importance of socioeconomic factors such as motivation, logistics, resources and communication. Communication with farmers in particular has been highlighted as a need in several studies on compliance with control recommendations [86, 87]. Consequently, most countries have specific communication, extension and education activities within their control programs for paratuberculosis. Improving knowledge is also important; about half the countries had active research objectives, many with emphasis on diagnosis. Knowledge gaps and research needs for paratuberculosis were reviewed recently [158].

While greater than two thirds of countries with a control program for paratuberculosis acknowledged support from various sectors of the community and government, there was also evidence of community polarisation within sectors. Uninfected producers in some economies may actually gain from the production losses in infected herds [54] which can also lead to disharmony. The experience articulated by Australia, France and Germany was that impacts of disease control measures can be felt differently by different parts of the farming community. Furthermore, community engagement can lead to dramatic change in policy. In Australia, community and government support of the control and prevention program declined between 1995 and 2016 amid rising criticism from some sectors about inequitable social, financial and personal impacts on affected producers. In response, the organisations representing the farming industries, as well as State governments, shifted the national program away from one with stringent regulatory measures to one in which individual farmers can choose to manage their own risk of paratuberculosis according to market demands, while observing civic (common law) responsibilities with respect to biosecurity. The national program is complemented by regulatory controls in the low-prevalence jurisdictions of Western Australia and Northern Territory where there was community demand for such measures.

An important aspect of this study was to assess whether control programs for paratuberculosis were considered to be successful by the countries that had them. The results were publicly available in almost 60% of countries and success measured against objectives was reported in 73% of countries during self-assessment. Some countries acknowledged the difficulties and problems in measuring success.

Establishing a sustainable control programme is difficult. This was the experience in developed countries such as the United States of America, Canada and Australia where control programs have expired in recent years, regardless of apparent or partial successes. Ultimately, cessation follows a redirection of resources which may be for any number of reasons. Securing funding for what inevitably needs to be a long term control activity is a problem. In some countries the food processing industries and farmers do not seem to have the desire or the capacity to provide funding. The test of public good (public versus private benefit) that is needed for government to become involved may not have been proven and the discussion between government and industry may have not reached a sufficient maturity for parties to agree to a funding model. This is not assisted by the ambiguous public health status of MAP, which has resulted in the conclusion that controlling MAP is an animal health responsibility (there are proven zoonotic conditions that attract public health funding) [58–60].

### Recommendations for future control programs

Paratuberculosis is a common disease with direct and indirect animal health, animal welfare and economic impacts. It may also affect public health. There is no doubt that it will spread and increase in prevalence and incidence if it is not controlled [32, 159, 160]. Therefore, control of this disease would seem to be important, but impediments exist: the lack of an international animal health code for paratuberculosis, the inconsistent approaches to public health assessments between countries, the lack of data on true prevalence and the long time frames required for control measures. Once a commitment is made to controlling the disease there are a suite of issues that should be addressed. Recommendations and possible actions are as follows:

1. *International guidelines and procedures for MAP*

The adoption of an international code for paratuberculosis by OIE, leading to universal acknowledgment of the principles and methods of control in relation to endemic and transboundary disease is essential. The lack of any general guidelines for paratuberculosis control as well as fragmentation of the paratuberculosis control programs in different countries creates a vacuum where animal health authorities do not know what to recommend and where governments can avoid costly actions if they wish to. This dialogue will require a discussion about conflicts between the control programs for bovine tuberculosis and paratuberculosis that arise due to diagnostics for bovine tuberculosis, specifically the intradermal test [161].

Elevation of the international discussion of the public health significance of MAP beyond health departments of individual countries is also needed. This should be through forums such as World Health Organization (WHO), building upon existing, credible, unbiased assessments [58–60] to obtain a consensus statement.

2. *Surveillance*

Uniform enhanced surveillance to establish or avoid overlooking the true prevalence of MAP is needed in many countries. This knowledge will help guide countries to the appropriate stages of control or eradication, and enable the selection and prioritisation of objectives as well as appropriate tools.

3. *Performance indicators*

Without performance indicators the industry or organization funding the control program may become less motivated to continue, but the negative aspects of using participation rates or clinical incidence data as signs of achievement should lead to exploration of other measures. One measure could include prevalence data based on objective surveillance and testing. The number of paratuberculosis notifications to the OIE relative to the size of the national herd, and reductions in true prevalence based on surveillance could be used. For countries declared free of paratuberculosis, objective monitoring data would be used for ongoing self-declaration of freedom from paratuberculosis.

4. *Research*

Diagnostic tests must be improved to assist surveillance as well as test and cull strategies. While new tests appear from time to time, such as the phage-test for viable MAP in blood or milk [162], or improved culture methods [163], critical evaluation should be required using stringent guidelines before tests are used in control programs [164]. Improved diagnostic tests are also needed for bovine tuberculosis, to differentiate infection from mycobacterial vaccination, and thereby enable wider use of existing commercial vaccines for paratuberculosis.

Evaluating the role of potential domestic or wild reservoir hosts for MAP, and the means of preventing spread of MAP from farmed livestock to wildlife populations is important. It may avert for paratuberculosis the problems experienced with control of bovine tuberculosis in countries such as the United Kingdom and New Zealand, and mitigate collateral damage in valued wildlife populations from the paratuberculosis epidemic in livestock.

New methods for studying paratuberculosis may enhance discovery of vaccine candidates and diagnostics. A vaccine that prevents infection may have advantages for controlling MAP over current vaccines. Direct comparisons between test and cull programs and vaccination programs for MAP are warranted in a range of species and countries to obtain evidence for application of vaccines, particularly where test and cull is impossible due to the scale of the problem or cultural practices. Vaccination for MAP may be able to be evaluated efficiently in developing countries that do not require bovine TB testing of cattle.

5. *Holistic approach*

How effective will control programs be if the disease is controlled in only one sector? Some countries in this study had control programs only for one type of livestock, typically dairy cattle, even though the condition was present in others. In Australia, paratuberculosis control in sheep has been managed independently from that in beef cattle despite co-grazing of pastures and evidence both for and against spread of MAP between these species [112, 165, 166], and control in beef cattle has sometimes been managed independently from dairy cattle despite dairy calves entering beef production facilities. Similarly, in countries as diverse as Iceland and New Zealand there has been exchange of MAP between different livestock sectors [112, 167]. In New Zealand, beef cattle and sheep raised together tended to harbor the same genetic variants of MAP, indicating no specialization of the agent [167]. While apparent host preferences of different strains of MAP have been reported [168], it is unclear whether the pathogen can evolve to take advantage of opportunities to establish new niches for its persistence. For these reasons an holistic approach is required for control of paratuberculosis.

6. *Improved communication among interested parties*

The success of any control program is dependent on the synergistic actions of farmers, veterinarians, diagnostic laboratories, breeding associations, food processors and state veterinary authorities among other stakeholders. All the involved parties need to communicate and share knowledge with each other. All parties should have all of the relevant information and understand all of the advantages of paratuberculosis control as well as all of the risks that failure to control may present now and in the future. All these stakeholders need to create a mutually supportive and transparent environment to allow control of paratuberculosis.

7. *Sustainability of control programs*

As is the case for bovine tuberculosis, the time frame for successful control of paratuberculosis is measured in decades. The need for sustainability of such an effort arguably must be determined and justified economically. Unless direct consumer or other market demands provide appropriate price signals, control programs will be under-valued by the farming industries and not sustained. Research studies to better understand and forecast consumer and market demands/pricing with respect to the animal welfare and public health aspects of MAP infection are warranted.

### Bias

Bias of various kinds is a potential problem in any survey. Chain referral sampling was used in this study to find people with relevant knowledge on paratuberculosis. This is a purposive, non-random sampling method and is therefore subject to selection bias. However, the advantages of its use were that people with “rare” knowledge and specific expertise and interest were able to be located through social networks, and it avoided other types of bias. Both geographic and Western cultural bias were overcome to some extent as participants were well distributed globally and usually were local people employed in institutions belonging to the country. Livestock producing countries outside the major developed economies of Europe and North America were represented although there were major gaps such as Russia, China and much of Africa. “Experts” were nominated but their recruitment was coordinated by one person (Whittington) who issued an invitation with information on the eligibility criteria and maintained all communications. This approach may have resulted in both the high response rate and the completion by most participants of most questions in the extremely detailed and lengthy questionnaire. Most of the participants were researchers or animal health policy developers and were aware of the requirement for objective data for scientific publications. Arguably they were diligent because they were aware that they would be a co-author of this paper from the beginning. Given the eligibility criteria, participants had good understanding of the control program in their country, reasons for establishing the program, objectives of the program, and funding of the program. Therefore, this information is unlikely to be biased.

Bias in provision of specific and detailed information was minimised through use of a structured questionnaire. This captured mainly categorical and ordinal responses; there were relatively few open questions. Importantly, “unknown” was able to be selected as a response to many questions, to avoid forcing an opinion in the absence of data, and sources of information were recorded and are documented in the supplementary tables. To this extent the results of the study are transparent. Questions about disease burden required quantitative data which often originated from a credible source (government report or a peer reviewed publication) and thus were unlikely to be biased. However, it is possible that selection bias may have led to estimates of disease prevalence in some countries higher than the actual prevalence of the disease, because of the particular focus of the experts. Alternatively, it could be underestimated in some countries due to prestige or social desirability bias. Data on prevalence of paratuberculosis are generally problematic, and the elevation of local or regional data for presentation at national level is acknowledged to be a limitation of this and other studies on paratuberculosis. Some of the information presented here was derived from participants’ opinions, for example on the success of the control program, or community and stakeholder engagement; opinions can be biased and therefore should be interpreted with caution.

## Conclusion

Based on this review of 48 countries, paratuberculosis was a common disease that will continue to spread if it is not controlled. However, there were many challenges for disease control flowing from the need to deal with very large animal populations spread across large numbers of herds, over a long time-frame. Many countries have an unknown prevalence and distribution of paratuberculosis, which can only be resolved by surveillance. Although we did not estimate the economic losses, based on data in the literature (see Background) they would already be considerable. Formal control programs were underway in 22 mostly developed countries, and were justified most commonly on animal health grounds, protecting market access and public health. However, articulation of a public health objective was very variable between countries. The most common objective was prevalence reduction, but several countries had a national or regional eradication program following successful control, and Sweden and Norway were considered to be in a surveillance phase. While control was voluntary in 60% of countries, programs were often supported by incentives and/or penalties for non-participation. Government funding was commonly involved and may be essential for sustainability; certainly, the availability of funding for long-term control activities was problematical. However, when assessed against their objectives, control programs were reported to be successful in 73% of 22 countries.

To enhance the control of paratuberculosis globally will require leadership, commencing with an agreed international code for paratuberculosis, describing the principles and methods of control. All ruminant livestock industries must be involved to prevent one industry becoming a reservoir of MAP for another industry. Public health assessments of MAP between countries also require an unbiassed harmonisation. Paratuberculosis detection and control will be improved through research on improved diagnostic tests and epidemiology. Vaccination against paratuberculosis, and the competing objectives of bovine tuberculosis and paratuberculosis control that exist because of use of the skin test for bovine tuberculosis surveillance, require re-evaluation. There are winners and losers in any control program, and for this reason all stakeholders must be educated about long-term goals and benefits in order to create a mutually supportive environment to allow for control of paratuberculosis.

## Additional files

Additional file 1: Questionnaire 27–11-18 final. Clean printout of on-line questionnaire document. (PDF 969 kb)

Additional file 2: **Table S1** to **S14** ver 25–2-19 final. Tabulated results. (PDF 445 kb)

Additional file 3: **Fig. S25-S2-S19** final. Data plots. (PDF 102 kb)

Additional file 4: Country-specific summaries 25–2-19 Tables. (PDF 280 kb)

Additional file 5: **Table S15** ver 25–2-19 final. (DOCX 43 kb)

## Data Availability

The datasets used and/or analysed during the current study are available from the corresponding author on reasonable request.

## Abbreviations

AGID: Agar gel immunodiffusion
CFT: Complement fixation test
EFSA: European Food Safety Authority
ELISA: Enzyme-linked immunosorbent assay
EU: European Union
IAP: International Association for Paratuberculosis
MAP: *Mycobacterium avium* subspecies *paratuberculosis*
OIE: World Organization for Animal Health
PCR: Polymerase chain reaction
SPS: Sanitary and Phytosanitary Agreement of WTO
WHO: World Health Organization
WTO: World Trade Organization
ZN: Ziehl-Neelsen
