# Supplementary material for: Control of paratuberculosis: who, why and how. A review of 48 countries
Source: BMC Vet Res. 2019 Jun 13;15:198. doi: 10.1186/s12917-019-1943-4 (PMC6567393; doi:10.1186/s12917-019-1943-4)
Supplement: Supplementary file 1 — Questionnaire 27–11-18 final. Clean printout of on-line questionnaire document. (PDF 969 kb) [file 12917_2019_1943_MOESM1_ESM.pdf]

International Control Programs for Paratuberculosis - Questionnaire

1. Welcome

Paratuberculosis is an important disease of livestock and control programs in one form or another have existed since at least as early as 1920. Over the last 20 years control programs for paratuberculosis have been implemented in many developed countries, and two excellent reviews were published between 2009 and 2014 covering the situation in some countries up until 2012; these brought information together on programmes mainly related to cattle (Nielsen 2009; Geraghty et al 2014). However, there are other important, susceptible, livestock species and since 2012 new programs have commenced (eg Ireland) while others have been discontinued or substantially modified (eg Australia, USA). In fact there is a lack of up to date, authoritative information and it is currently impossible to ascertain from any single source what is being done about paratuberculosis in different countries, and the reasons for action or inaction on this disease. Consequently animal health authorities are not in a good position to make recommendations to their own governments or domestic animal industries, and sometimes are forced to respond quickly to changing circumstances with incomplete information.

Mycobacterium avium **subsp.** ***paratuberculosis*** **is abbreviated as MAP in this questionnaire.**

Thank you for participating in this project. Your input is important because it will help fill the international knowledge gap.

Geraghty, T., Graham, D.A., Mullowney, P., More, S.J., 2014. A review of bovine Johne's disease control activities in 6 endemically infected countries. Preventive Veterinary Medicine 116, 1-11.

Nielsen, S. 2009. Programmes on paratuberculosis in Europe. Proceedings of the 10th International Colloquium on Paratuberculosis. International Association for Paratuberculosis, pp. 101-108.

International Control Programs for Paratuberculosis - Questionnaire

2. Privacy statement and consent

Privacy and consent

1. This questionnaire is not anonymous.
2. All participants have accepted a nomination to provide information about paratuberculosis in their region or country, and have obtained permission to participate from their employer (if appropriate).
3. All participants are considered to be research collaborators in a project that uses this questionnaire to obtain information in a consistent format to facilitate analysis and publication of findings in a scientific manner.
4. All participants will be acknowledged by name, institution and country in a report that is produced using data analysed from this survey. All participants will be sent a copy of a draft report prior to its distribution or publication; all participants will be asked to comment and consent within one month, such consent not to be unreasonably withheld.
5. You agree not to provide confidential information that cannot be published. When completing questions you can enter "not applicable" or "N/A" in order to proceed to the next question in the event that you are unable or not entitled to provide a response to that question.
6. Responses to questions will be aggregated, analysed, summarised. Regional or country identification will be reported.
7. The survey will be configured to enable you to modify or correct your responses until such time as the survey is closed off for analysis.
8. According to information provided by Survey Monkey, through use of the Survey Monkey software, personal data collected from participants may be transferred to various countries, including the United States and other locations. Here is a link to the Survey Monkey Privacy Policy. The security of data in the Survey Money platform is described in Security.
9. Participants can contact the survey manager Richard Whittington by email at any time:- richard.whittington@sydney.edu.au

* 1. Do you agree to the above privacy terms? By clicking Yes, you consent that you are willing to complete this questionnaire.

◯ Yes

◯ No

Navigation buttons
NEXT - saves responses and proceeds to next page.
PREV - go back to previous page to review or edit a response.

3. Your details

In this section you will be asked to provide contact details to enable further direct communication and follow up on specific questions if needed.

You will be asked whether you are providing information about a region or an entire country .

If you are responding about more than one region or country , you will need to complete a separate questionnaire for each one, unless they are all very similar, in which case you can simply complete one questionnaire and list the regions/countries in Question 4. If you are in doubt please contact Richard Whittington by e-mail: richard.whittington@sydney.edu.au

If you need to complete more than one copy of the questionnaire , separate copies can be issued to you electronically. To arrange this please contact Richard Whittington by e-mail: richard.whittington@sydney.edu.au

* 2. Please enter your details

Your name

The name of your institution

e-mail

* 3. Are you responding about a country or a region in this copy of the questionnaire?

* 4. Which country or region are you going to provide information about in this questionnaire? (you can complete a separate copy of the questionnaire for another region)

Geographic zone (Asia, Africa, North America, South America, Europe, Pacific Islands, or Australasia)

Country

Region
(Important - enter N/A here if you are responding about the whole country)

5. If more than one person from the list of participants**from your country** is contributing to the responses in this copy of the questionnaire, please list their names and email addresses here.

* 6. What is your main interest, expertise or role in paratuberculosis control. Select any that apply.

- [ ] Field aspects
- [ ] Communications and education aspects
- [ ] Laboratory aspects
- [ ] Research aspects
- [ ] Management and coordination aspects
- [ ] Other

If you answered Other please describe

Navigation buttons
NEXT - saves responses and proceeds to next page.
PREV - go back to previous page to review or edit a response.

4. Information about the animal population

* 7. What is the size of the animal population in the country or region?
Please provide approximate numbers if possible.
One response is required in each row of the table.

	Not applicable	Unknown	<1,000	1,000 to 10,000	10,000 to 100,000	100,000 to 1,000,000	1,000,000 to 10,000,000	> 10,000,000
Cattle - dairy	○	○	○	○	○	○	○	○
Cattle - beef	○	○	○	○	○	○	○	○
Sheep	○	○	○	○	○	○	○	○
Goats	○	○	○	○	○	○	○	○
Camelids (define type in comments box)	○	○	○	○	○	○	○	○
Deer - farmed	○	○	○	○	○	○	○	○
Other	○	○	○	○	○	○	○	○

Other (please specify species or type e.g. cattle-feedlot)

[ ]

The next few questions are about the number of farms and herd size. These are optional questions, but please complete them if you have any information.

8. How many farms are in the country or region? Please enter a number.

Cattle - dairy [ ]

Cattle - beef [ ]

Sheep [ ]

Goats [ ]

Camelids [ ]

Deer - farmed [ ]

Other - please specify then enter a number [ ]

9. What is the average herd size in the country or region? Please enter a number.

Cattle - dairy

Cattle - beef

Sheep

Goats

Camelids

Deer - farmed

Other - please specify then enter a number

10. What is the minimum herd size? Please enter a number.

Cattle - dairy

Cattle - beef

Sheep

Goats

Camelids

Deer - farmed

Other - please specify then enter a number

11. What is the maximum herd size? Please enter a number.

Cattle - dairy

Cattle - beef

Sheep

Goats

Camelids

Deer - farmed

Other - please specify then enter a number

Navigation buttons
NEXT - saves responses and proceeds to next page.
PREV - go back to previous page to review or edit a response.

5. Background information about paratuberculosis (#1)

Notification of paratuberculosis and herd level prevalence

Herd level prevalence is the proportion of herds that are affected.

* 12. Is paratuberculosis (Johne's disease) or infection with MAP notifiable to the competent authority in your country or region? A response is required in each row of the table.

	Yes	No	Not applicable
Cattle - dairy	○	○	○
Cattle - beef	○	○	○
Sheep	○	○	○
Goats	○	○	○
Camelids	○	○	○
Deer - farmed	○	○	○
Other	○	○	○

Other (please specify)

* 13. What is the herd-level prevalence of paratuberculosis in your country or region? A response is required in each row of the table.

	Not applicable	Unknown	<1%	1-10%	10-20%	20-40%	>40%
Cattle - dairy	○	○	○	○	○	○	○
Cattle - beef	○	○	○	○	○	○	○
Sheep	○	○	○	○	○	○	○
Goats	○	○	○	○	○	○	○
Camelids	○	○	○	○	○	○	○
Deer - farmed	○	○	○	○	○	○	○
Other - please specify then enter a number	○	○	○	○	○	○	○

Other (please specify)

* 14. What is the source of information for herd-level prevalence estimates? Select any that apply; a response is required in every row.

	Not applicable	Apparent prevalence based on serology	True prevalence based on serology	Apparent prevalence based on bulk milk ELISA	True prevalence based on bulk milk ELISA	Abattoir monitoring	Other active surveillance	Passive surveillance	Other
Cattle - dairy	☐	☐	☐	☐	☐	☐	☐	☐	☐
Cattle - beef	☐	☐	☐	☐	☐	☐	☐	☐	☐
Sheep	☐	☐	☐	☐	☐	☐	☐	☐	☐
Goats	☐	☐	☐	☐	☐	☐	☐	☐	☐
Camelids	☐	☐	☐	☐	☐	☐	☐	☐	☐
Deer - farmed	☐	☐	☐	☐	☐	☐	☐	☐	☐
Other	☐	☐	☐	☐	☐	☐	☐	☐	☐

Please provide more information if possible:
i) If you answered Other active surveillance or Other, please describe.
ii) When were the prevalence estimates made?
iii) Please provide a reference if a study was published.

Navigation buttons

NEXT - saves responses and proceeds to next page.

PREV - go back to previous page to review or edit a response.

6. Background information about paratuberculosis (#2)

Within herd prevalence is the proportion of affected animals within herds that are affected.

* 15. What is the within-herd prevalence of paratuberculosis for the infected herds/flocks? A response is required in each row of the table.

	Not applicable	Unknown	<1%	1-5%	5-10%	10-15%	>15%
Cattle - dairy	◯	◯	◯	◯	◯	◯	◯
Cattle - beef	◯	◯	◯	◯	◯	◯	◯
Sheep	◯	◯	◯	◯	◯	◯	◯
Goats	◯	◯	◯	◯	◯	◯	◯
Camelids	◯	◯	◯	◯	◯	◯	◯
Deer - farmed	◯	◯	◯	◯	◯	◯	◯
Other	◯	◯	◯	◯	◯	◯	◯

Other (please specify)

* 16. What is the source of information for within herd prevalence estimates? Select any that apply; a response is required in every row.

	Not applicable	Apparent prevalence based on serology	True prevalence based on serology	Apparent prevalence based on individual milk ELISA	True prevalence based on individual milk ELISA	Apparent or true prevalence based on faecal culture or PCR	Abattoir monitoring	Other active surveillance	Passive surveillance	Other
Cattle - dairy	☐	☐	☐	☐	☐	☐	☐	☐	☐	☐
Cattle - beef	☐	☐	☐	☐	☐	☐	☐	☐	☐	☐
Sheep	☐	☐	☐	☐	☐	☐	☐	☐	☐	☐
Goats	☐	☐	☐	☐	☐	☐	☐	☐	☐	☐
Camelids	☐	☐	☐	☐	☐	☐	☐	☐	☐	☐
Deer - farmed	☐	☐	☐	☐	☐	☐	☐	☐	☐	☐
Other	☐	☐	☐	☐	☐	☐	☐	☐	☐	☐

Please provide more information if possible:
i) If you answered Other active surveillance or Other, please describe.
ii) When were the prevalence estimates made?
iii) Please provide a reference if a study was published.

Navigation buttons
NEXT - saves responses and proceeds to next page.
PREV - go back to previous page to review or edit a response.

7. Background information about paratuberculosis (#3)

* 17. If it is notifiable, in your opinion is paratuberculosisis (generally meaning clinical or sub-clinical Johne's disease) or MAP infection likely to be under-reported to authorities?

- Not applicable
- Unknown
- No
- Yes

If you answered Yes, please give reasons for under-reporting

* 18. In your opinion, is the prevalence of paratuberculosisis (generally meaning clinical or sub-clinical Johne's disease) or MAP infection likely to be under-estimated?

- Not applicable
- Unknown
- No
- Yes

If you answered Yes, please give reasons for under-estimating prevalence

* 19. Are free-living ruminants or wildlife in this country or region known to be infected with MAP?

- ◯ Not-applicable - there are no free-living ruminants or wildlife
- ◯ Unknown
- ◯ No
- ◯ Yes

If you answered Yes, please describe which species and the circumstances

Navigation buttons
NEXT - saves responses and proceeds to next page.
PREV - go back to previous page to review or edit a response.

8. Background information about disease control

Definition and scope for livestock disease control programs in general.

Included: control program means:

- an ongoing process of measures intended to interfere with the unrestrained occurrence of the disease (based on Thrusfield 2005).
- the measures must include elements of planning, coordination, documentation and evaluation, and may be conducted locally, regionally, or nationally.
- examples of measures include: estimation of prevalence to inform the decision making; surveillance to detect infected herds; control of the infection in infected herds and flocks; measures to prevent introduction of a disease into free populations.
- certification of herds and flocks as a low-risk source is included here.
- objectives may range from preventing an increase in prevalence through to eradication.

Excluded: control program does not mean:

- work done by a veterinarian on cases of disease on a farm.
- a private herd health program (also known as a private health and productivity scheme) offered by a veterinarian to an individual herd/farm.

* 20. In the period 2012-2018 is there a control program (or a surveillance program in the event of freedom) for a livestock disease other than paratuberculosis in the country or region? Answer yes if it started, ended or operated throughout this period. A response is required in each row of the table.

	Yes	No	Not applicable
Is there a program for Bovine Tuberculosis?	○	○	○
Is there a program for Bovine Brucellosis?	○	○	○
Is there a program for Rinderpest?	○	○	○
Is there a program for Foot and Mouth Disease?	○	○	○
Other - are there programs for other diseases of livestock (any species)? Give examples in the box below.	○	○	○

If you answered Other, please specify the species and the diseases or give some examples if there are too many to list them all.

Navigation buttons
NEXT - saves responses and proceeds to next page.
PREV - go back to previous page to review or edit a response.

9. Background information about paratuberculosis control (#1)

Definition and scope for paratuberculosis control programs:

Included: a control program for paratuberculosis means:

- an ongoing process of measures intended to interfere with the unrestrained occurrence of the disease (based on Thrusfield 2005).
- the measures must include elements of planning, coordination, documentation and evaluation, and may be conducted locally, regionally, or nationally.
- examples of measures include: estimation of prevalence to inform the decision making; surveillance to detect infected herds; control of the infection in infected herd and flocks; measures to prevent introduction of MAP into free populations;
- certification of herds and flocks as a low-risk source is part of a control program for paratuberculosis in some countries and is included here.
- objectives may range from preventing an increase in prevalence through to eradication.

Excluded: a control program for paratuberculosis does not mean:

- work done by a veterinarian on a case of paratuberculosis on a farm.
- a private herd health program (also known as a private health and productivity scheme) offered by a veterinarian to an individual herd/farm, in which paratuberculoisis control is included.
- However, some questions about these activities are included in this questionnaire.

Time period:

- the main part of the survey covers the period 2012 to 2018
- however there are questions in this section about control programs that ended before this period, or that are continuous or that will commence afterwards.

* 21. Is there a control program(s) for paratuberculosis operating in this country or region in the period 2012 to 2018? Answer yes if it started or ended or operated throughout this period.

◯ No

◯ Yes

* 22. If there was no control program for paratuberculosis operating in this country or region in the period 2012 to 2018, was there a control program(s) for paratuberculosis that ended prior to 2012?

- ○ No
- ○ Yes

If you answered Yes, please provide more information:
i) When did this control program end?
ii) Why did the control program end?
If more than one program, please provide details.

23. If you answered Yes to the previous question, what were the main outcomes from the control program(s) that ended before 2012?

* 24. Is there a long-term or continuous control program for paratuberculosis in this country or region, i.e. one that commenced before 2012, is operating now, and will continue after 2018?

- ○ No
- ○ Yes

If you answered Yes, please specify when this control program commenced.

Navigation buttons
NEXT - saves responses and proceeds to next page.
PREV - go back to previous page to review or edit a response.

10. Background information about paratuberculosis control (#2)

* 25. If there was no control program for paratuberculosis operating in the period 2012 to 2018, is a new control programme for paratuberculosis foreseen to commence in this country or region in 2018-2020?

- ○ No
- ○ Yes

If you answered Yes, please describe the main features of the new control program that is foreseen

* 26. If there was no national or regional control program for paratuberculosis in the period 2012-2018, please indicate why not:
Select any that apply (or select "Not applicable" if there is a control program and go to the next question).

- ☐ Not applicable - there is a control program
- ☐ Economic constraints
- ☐ Animal health resources are currently deployed to tackle other priority diseases
- ☐ Lack of national/regional animal health capacity, infrastructure or operational resources
- ☐ Lack of laboratory diagnostic services
- ☐ Lack of feasibility due to inadequate control tools (eg. poor diagnostic tests, poor vaccines)
- ☐ Paratuberculosis is not prevalent at herd or individual animal levels and is not considered to be a problem relative to other animal health issues
- ☐ Paratuberculosis is present but is not considered to affect the animal population
- ☐ Paratuberculosis vaccine is available for use by farmers (there is no coordinated control program)
- ☐ Other

If you selected Other, please provide reasons with reference to particular species if appropriate

* 27. Do veterinarians or others in this country or region provide advice on paratuberculosis to individual herds/farms as part of a private herd health program (also called a private health and productivity scheme), even if there is no nationally or regionally coordinated control program for paratuberculosis?

- ◯ Unknown
- ◯ No
- ◯ Yes

If you answered Yes, please provide more information or enter N/A to continue to the next question

* 28. Do veterinarians or others in this country or region provide advice to individual farmers on paratuberculosis, for example after diagnosis of a clinical case, even if there is no control program for paratuberculosis?

- ◯ Unknown
- ◯ No
- ◯ Yes

If you answered Yes, please provide more information or enter N/A to proceed to the next question.

* 29. Please confirm that there is a control program for paratuberculosis in this country or region that started, ended or was operational in the period 2012-2018.

If you select Yes, you can proceed to the next section of the questionnaire.

If you select No, you will be directed to the end of the survey.

- ◯ Yes
- ◯ No

Navigation buttons
NEXT - saves responses and proceeds to next page.
PREV - go back to previous page to review or edit a response.

11. Paratuberculosis Control Programs in 2012 - 2018 (#1)

Program structure and objectives

A single national control program may have different components, e.g. a test and cull program and a herd assurance program. For the purposes of this questionnaire, please regard these as components of a single national control program.

However, there may be situations where there is more than one national control program. There is an option to report that in the table below.

* 30. Is the control program national or regional? Select the option that best describes the control program in each species in the country or region about which you are responding. A response is required in each row.

	Not applicable - species is not present or is not the subject of a control program	A single national control program	More than one national control program	A regional control program
Cattle - dairy	◯	◯	◯	◯
Cattle - beef	◯	◯	◯	◯
Sheep	◯	◯	◯	◯
Goats	◯	◯	◯	◯
Camelids	◯	◯	◯	◯
Deer -farmed	◯	◯	◯	◯

Other (please specify)

* 31. In the case of a regional control program, select the option that best describes the situation **in the country**. A response is required in each row.

	Not applicable - it is a national program, species not present or not subject to a control program	Regional control programs are coordinated nationally	Regional control programs are coordinated regionally
Cattle - dairy	◯	◯	◯
Cattle - beef	◯	◯	◯
Sheep	◯	◯	◯
Goats	◯	◯	◯
Camelids	◯	◯	◯
Deer - farmed	◯	◯	◯

Other (please specify)

* 32. In the case of a regional control program, select the option that best describes the situation **in the country**. Select any that apply. A response is required in each row.

	Not applicable	A control program exists in every region of the country with livestock	A control program exists in most regions of the country with livestock	A control program exists in some regions of the country with livestock
Cattle - dairy	◯	◯	◯	◯
Cattle - beef	◯	◯	◯	◯
Sheep	◯	◯	◯	◯
Goats	◯	◯	◯	◯
Camelids	◯	◯	◯	◯
Deer - farmed	◯	◯	◯	◯
Other - please specify below	◯	◯	◯	◯

Other (please specify)

* 33. In the case of a regional control program, select the option that best describes the situation **in the country**. Select any that apply. A response is required in each row.

	Not applicable	The control programs in each region are different	The control programs in each region are very similar or identical
Cattle - dairy	◯	◯	◯
Cattle - beef	◯	◯	◯
Sheep	◯	◯	◯
Goats	◯	◯	◯
Camelids	◯	◯	◯
Deer - farmed	◯	◯	◯

Other (please specify)

* 34. What are the main objectives of the control program?
Examples might be i) to reduce the prevalence of MAP infection in livestock, ii) reduce the incidence of clinical cases of paratuberculosis, iii) to provide assurance about herd status to provide certified replacement stock, iv) to reduce MAP in the human food chain.

Please list the objectives. If there is more than one control program, please list the objectives of each separately.

Please provide a reference if available (eg the URL of the website for the control program, or a document)

Navigation buttons
NEXT - saves responses and proceeds to next page.
PREV - go back to previous page to review or edit a response.

12. Paratuberculosis Control Programs in 2012 - 2018 (#2)

Duration and performance indicators

* 35. In what year did the control program start?
Please list years for each control program if there is more than one.

36. Please list any major chronological events, such as addition of new components or new regions. Please list separately for each control program if there is more than one.

* 37. Is the control program time limited?

- No
- Yes

If you answered Yes, when will it end?

* 38. Are there performance indicators for the control program? For example: i) participation rates (targets with regard to numbers of herds or flocks participating; ii) achievements (targets such as reducing the number of affected herds, or increasing the number of accredited herds)

- No
- Yes

If you answered Yes, please list the performance indicators

Navigation buttons
NEXT - saves responses and proceeds to next page.
PREV - go back to previous page to review or edit a response.

13. Paratuberculosis Control Programs in 2012 - 2018 (#3)

Reasons for program

* 39. What are the reasons for having the control program? Select any that apply.

- [ ] Animal health
- [ ] Animal welfare
- [ ] Reducing production losses
- [ ] Public health
- [ ] Maintaining trade, regional or international
- [ ] Unknown
- [ ] Other

If you selected Other, please provide more information

* 40. What sources of information inform the reasons selected in the previous question. For example, are production loss data available for the region or country; which public health document is considered relevant? Please provide details/references.

* 41. Is there a statement about public health, such as reducing human exposure to *Mycobacterium avium* subsp. *paratuberculosis* (MAP), in documents or communications about the control program?

- ( ) No
- ( ) Yes

If you answered Yes, please provide details including a reference to a document or the URL

Navigation buttons

NEXT - saves responses and proceeds to next page.

PREV - go back to previous page to review or edit a response.

14. Paratuberculosis Control Programs in 2012 - 2018 (#4)

Review of program

* 42. Has the control program been reviewed?

- ◯ Yes
- ◯ No
- ◯ Unknown

43. If the control program has been reviewed, when was this done?

44. If the control program has been reviewed, who conducted the review (for example was it internal or external)?

45. If the control program was reviewed, did it change as a result of the review? A response is required in each row of the table.

	Yes	No
Did it change substantially?	◯	◯
Did the objectives change?	◯	◯
Did the operations/methods change?	◯	◯
Was the control program terminated?	◯	◯
Was the control program continued or extended?	◯	◯

46. If the control program was reviewed, please add further comments if you wish.

Navigation buttons
NEXT - saves responses and proceeds to next page.
PREV - go back to previous page to review or edit a response.

15. Paratuberculosis Control Programs in 2012 - 2018 (#5)

Leadership and funding

* 47. Who leads the control program? Select any that apply.

- [ ] Government
- [ ] Industry association/organisation - milk
- [ ] Industry association/organisation - meat
- [ ] Industry association - livestock trading
- [ ] Food processing industry
- [ ] Farmer collective/association/organisation
- [ ] Veterinary association/organisation
- [ ] Private veterinarians
- [ ] Individual farmers
- [ ] Other

If you answered Other please describe

* 48. How are leadership and coordination of the control program funded? Please check that the options selected total 100% (e.g. Farmer 50% plus Other 50%). A response is required in each row of the table.

	0%	25%	50%	75%	100%
Government $	○	○	○	○	○
Farmer organisation (i.e. a collective or society) $	○	○	○	○	○
Farmer $	○	○	○	○	○
Processing industry (meat, milk) $	○	○	○	○	○
Other $	○	○	○	○	○

If you answered Other please describe

* 49. How are the operations of the control program funded? Please check that the options selected in each row total 100% (e.g. Government 50% plus Farmer 50%). A response is required in each row of the table.

	0%	25%	50%	75%	100%
Government $	○	○	○	○	○
Farmer organisation (i.e. a collective or society) $	○	○	○	○	○
Farmer $	○	○	○	○	○
Processing industry (meat, milk) $	○	○	○	○	○
Other $	○	○	○	○	○

If you answered Other please describe

Navigation buttons
NEXT - saves responses and proceeds to next page.
PREV - go back to previous page to review or edit a response.

16. Paratuberculosis Control Programs in 2012 - 2018 (#6)

Participation and compensation

Control programs may be voluntary or compulsory, but the definitions for these categories can be ambiguous. A program may be voluntary, but there might be a severe market penalty for not participating, in which case farmers may be forced to participate. In this questionnaire a program is considered to be compulsory when there is a legal requirement to participate.

* 50. Is there a legal requirement to participate in the control program? In other words is the control program compulsory?

- ◯ No
- ◯ Yes

If Yes, please describe

* 51. Are financial or other incentives or restrictions in place to stimulate farmers to participate in the control program?

- ◯ No
- ◯ Yes

52. If you answered Yes to the previous question, what type of incentives are in place?

- ☐ Higher prices are paid to farmers who are in the control program for their animals or animal products (for example by livestock traders, stud breeders or milk processors)?
- ☐ There are local market access restrictions for non-participating farmers (for example they are unable to trade their livestock with farmers in other regions, or they are unable to sell their milk to a local processor)
- ☐ There are other incentives

Please describe the incentives or restrictions in more detail

* 53. Is there financial compensation for farmers whose livestock are affected by paratuberculosis?

- ◯ No
- ◯ Yes

54. If you responded Yes in the previous question, what is the nature of the financial compensation for farmers? Select any that apply.

- ☐ Cost of testing
- ☐ Cost of culling infected livestock
- ☐ Value of culled animals
- ☐ Other

If you answered Other please describe

Navigation buttons
NEXT - saves responses and proceeds to next page.
PREV - go back to previous page to review or edit a response.

THE UNIVERSITY OF SYDNEY

International Control Programs for Paratuberculosis - Questionnaire

17. Paratuberculosis Control Programs in 2012 - 2018 (#7)

Components and implementation

* 55. What types of tools are used in the control program? Select any that apply.

- [ ] Stamping out infected herds/flocks
- [ ] Hygienic calf/lamb/kid rearing
- [ ] Environmental and pasture management
- [ ] Cull clinical cases
- [ ] Test and cull subclinical cases
- [ ] Vaccination
- [ ] Herd/flock assurance certification
- [ ] Individual animal assurance certification
- [ ] Farm level biosecurity plan to prevent introduction of infection
- [ ] National biosecurity plan to prevent introduction of infection
- [ ] Regional biosecurity plan to prevent introduction of infection
- [ ] Communication program
- [ ] Research program
- [ ] Other

If you answered Other please describe

* 56. Concerning the role of veterinary services, who implements the control program at farm level?

- ( ) Government veterinarians or para-veterinary staff (eg animal health officers)
- ( ) Private veterinarians or para-veterinary staff (eg private animal health officers)
- ( ) Both Government and Private veterinarians or para-veterinary staff
- ( ) Other (please describe)

Navigation buttons
NEXT - saves responses and proceeds to next page.
PREV - go back to previous page to review or edit a response.

18. Paratuberculosis Control Programs in 2012 - 2018 (#8)

Protocols, definitions and rules

A control program may be accompanied by purpose-designed documents which provide case definitions, rules and procedures for operational aspects. This is called a "manual" for the purposes of this questionnaire. Alternatively, a program may be based on briefer documentation but refer to other sources for detailed information on case definitions and methods of control (for example case definition terminology for paratuberculosis: BMC Veterinary Research 2017, 13:328 DOI 10.1186/s12917-017-1254-6).

* 57. Is there a manual describing the case definitions, rules and procedures for the paratuberculosis control program?

- ◯ No
- ◯ Yes

* 58. If there is a manual, is it publicly available?

- ◯ Not applicable (there is no manual)
- ◯ No
- ◯ Yes

If you answered Yes, please provide a URL or reference for citation purposes

* 59. If there is not a specific manual, are other sources of information used for case definitions, and operational aspects?

- ◯ Not applicable (there is a manual)
- ◯ No
- ◯ Yes

If you answered Yes please provide a URL or reference to the other sources of information for citation purposes

* 60. This question refers to the control program manual or other sources of information. A response is required in each row of the table.

	Yes	No	Not applicable
Are definitions for paratuberculosis "infected" or "diseased" animals and/or herds provided?	○	○	○
Are terms such as "control" or "eradication"defined?	○	○	○
Are methods of diagnosis and surveillance described in detail?	○	○	○
Are methods of control described in detail?	○	○	○
Are the rules and regulations described in detail?	○	○	○

Navigation buttons
NEXT - saves responses and proceeds to next page.
PREV - go back to previous page to review or edit a response.

19. Paratuberculosis Control Programs in 2012 - 2018 (#9)

Diagnostic tests

* 61. Which types of diagnostic tests are recommended in the official strategies or are most commonly used in 2012-2018 in each species? Select any that apply; a response is required in each row.

	Test is not used	Cattle - dairy	Cattle - beef	Sheep	Goats	Camelids	Deer - farmed	Other species/type- please specify below
Blood ELISA	☐	☐	☐	☐	☐	☐	☐	☐
Blood agar gel immunodiffusion assay	☐	☐	☐	☐	☐	☐	☐	☐
Milk ELISA – bulk milk	☐	☐	☐	☐	☐	☐	☐	☐
Milk ELISA – individual animal	☐	☐	☐	☐	☐	☐	☐	☐
Faecal culture – individual	☐	☐	☐	☐	☐	☐	☐	☐
Faecal culture – pooled	☐	☐	☐	☐	☐	☐	☐	☐
Faecal PCR - individual	☐	☐	☐	☐	☐	☐	☐	☐
Faecal PCR - pooled	☐	☐	☐	☐	☐	☐	☐	☐
Environmental faecal test – culture or PCR	☐	☐	☐	☐	☐	☐	☐	☐
Pathology	☐	☐	☐	☐	☐	☐	☐	☐
Intradermal skin test	☐	☐	☐	☐	☐	☐	☐	☐
Complement fixation test	☐	☐	☐	☐	☐	☐	☐	☐
Faecal smear stained with ZN	☐	☐	☐	☐	☐	☐	☐	☐
Other test - please specify below	☐	☐	☐	☐	☐	☐	☐	☐

If you answered Other species or Other test please describe

* 62. Which types of diagnostic tests are recommended in the official strategies or are most commonly used in 2018 for each purpose? Select any that apply; a response is required in each row.

	Test is not used	Individual animal diagnosis	Individual animal certification/assurance	Herd-level screening	Herd-level certification/assurance	Other purpose - please specify below
Blood ELISA	☐	☐	☐	☐	☐	☐
Blood agar gel immunodiffusion assay	☐	☐	☐	☐	☐	☐
Milk ELISA – bulk milk	☐	☐	☐	☐	☐	☐
Milk ELISA – individual animal	☐	☐	☐	☐	☐	☐
Faecal culture – individual	☐	☐	☐	☐	☐	☐
Faecal culture – pooled	☐	☐	☐	☐	☐	☐
Faecal PCR - individual	☐	☐	☐	☐	☐	☐
Faecal PCR - pooled	☐	☐	☐	☐	☐	☐
Environmental faecal test – culture or PCR	☐	☐	☐	☐	☐	☐
Pathology	☐	☐	☐	☐	☐	☐
Intradermal skin test	☐	☐	☐	☐	☐	☐
Complement fixation test	☐	☐	☐	☐	☐	☐
Faecal smear stained with ZN	☐	☐	☐	☐	☐	☐
Other test - please specify below	☐	☐	☐	☐	☐	☐

If you answered Other test or Other purpose please describe

Navigation buttons
NEXT - saves responses and proceeds to next page.
PREV - go back to previous page to review or edit a response.

20. Paratuberculosis Control Programs in 2012 - 2018 (#10)

Communication, extension, education, research

A control program may include communication, education and extension activities. Education has an important role in influencing attitudes and opinion, which in turn can affect the success of a control program (Thrusfield 2007). Research may also be mandated within a control program, dependent on the available veterinary infrastructure, and its outcomes can improve a control program.

Some examples of these activities include:
i) dissemination of information about objectives and progress through newsletters, a website, or press releases to newpapers and other media
ii) training/education of farmers in disease recognition, tools and procedures for control of disease.
iii) training of veterinarians, who may become accredited to provide services to farmers in the control program
iv) extension of the latest information and advice on control methods to the rural community through workshops and farmer field days
v) research on improved diagnostic methods,
vi) research on validation of diagnostic methods in local livestock populations
vii) research on farmer perceptions about disease control

* 63. Are communication, extension, or education activities included as part of the control program?

- ◯ No
- ◯ Yes

If you answered Yes, what are the objectives and activities of the communication/extension/education components?

* 64. Is research included as part of the control program?

- No
- Yes

If you answered Yes, what are the objectives of the research?

Navigation buttons
NEXT - saves responses and proceeds to next page.
PREV - go back to previous page to review or edit a response.

THE UNIVERSITY OF SYDNEY

International Control Programs for Paratuberculosis - Questionnaire

21. Paratuberculosis Control Programs in 2012 - 2018 (#11)

Results of the control program

* 65. Are results from the control program publicly available? For example, are there objective data about progress towards meeting the objectives, or number of farms enrolled over time?

○ No

○ Yes

If If you answered Yes,
i) please describe the type of results that are available
ii) please provide a reference or source for the information

* 66. In your opinion, has the control program been successful? Please use this criterion: stakeholders or beneficiaries (e.g. governments, industry associations or farmer collectives, individual farmers, food processors) perceive a benefit and progress has been made towards meeting at least some of the objectives.

○ No

○ Yes

○ It is too early to tell

○ I am unable to decide

If you answered Yes or No, please describe why the program has or has not been successful.

Navigation buttons
NEXT - saves responses and proceeds to next page.
PREV - go back to previous page to review or edit a response.

22. Paratuberculosis Control Programs in 2012 - 2018 (#12)

Community support for control program

The opinion of farmers and public opinion are recognised to be important factors affecting the success of a control program. For this reason the next two questions are about the support of stakeholders for the control program.

* 67. Is there agreement/support for the control program from the following stakeholders/beneficiaries? A response is required in each row of the table.

	Yes	No	Unknown	Not applicable
Government	○	○	○	○
Industry association/organisation - milk	○	○	○	○
Industry association/organisation - meat	○	○	○	○
Industry association/organisation - livestock trading	○	○	○	○
Farmer collective/association/organisation	○	○	○	○
Veterinary association/organisation	○	○	○	○
Private veterinarians	○	○	○	○
Food processing industry	○	○	○	○
Individual farmers	○	○	○	○
Other	○	○	○	○

If you answered Yes or No to Other please describe

68. If you answered Yes to the previous question, how do you confirm, assess or measure community/stakeholder support?

That was the last question. Thank you for your time and effort in completing the questionnaire.

When you select DONE, your responses will be submitted.

You will be able to return to the questionnaire to review, revise or edit your responses at any time until midnight on Friday 30th March 2018 (Sydney Australia time, GMT + 10 hours) when the questionnaire will be closed.
