# Supplementary material for: Control of paratuberculosis: who, why and how. A review of 48 countries
Source: BMC Vet Res. 2019 Jun 13;15:198. doi: 10.1186/s12917-019-1943-4 (PMC6567393; doi:10.1186/s12917-019-1943-4)
Supplement: Supplementary file 2 — Table S1 to S14 ver 25–2-19 final. Tabulated results. (PDF 445 kb) [file 12917_2019_1943_MOESM2_ESM.pdf]

Supplementary Table 1. Animal populations

Country	Dairy Cattle	Beef Cattle	Sheep	Goat	Camelid	Deer	Other	Comments
Argentina	1,000,000 to 10,000,000	> 10,000,000	> 10,000,000	100,000 to 1,000,000	100,000 to 1,000,000	10,000 to 100,000	1,000 to 10,000	South American camelids: guanaco (600,000), llama, vicuña, alpaca, red deer and others cervids. Wild fauna: deer
Australia	1,000,000 to 10,000,000	> 10,000,000	> 10,000,000	1,000,000 to 10,000,000	100,000 to 1,000,000	10,000 to 100,000	Not applicable	Approx 250,000 alpaca, 10,000 llama, small numbers of farmed camel, and many 10,000's of feral camel.
Austria	Unknown	Unknown	100,000 to 1,000,000	10,000 to 100,000	1,000 to 10,000	Unknown	Not applicable	Total number of cattle: 1,954,391 New world camelids: 4,000-6,000
Bangladesh	> 10,000,000	> 10,000,000	1,000,000 to 10,000,000	> 10,000,000	Not applicable	Unknown	1,000,000 to 10,000,000	Buffalo 1,478,000
Belgium	100,000 to 1,000,000	1,000,000 to 10,000,000	100,000 to 1,000,000	10,000 to 100,000	Unknown	Unknown	Not applicable	
Bhutan	100,000 to 1,000,000	Not applicable	10,000 to 100,000	10,000 to 100,000	Not applicable	Not applicable	Not applicable	
Brazil	> 10,000,000	> 10,000,000	> 10,000,000	1,000,000 to 10,000,000	Unknown	Unknown	1,000,000 to 10,000,000	Buffalo – 948,103
Canada	100,000 to 1,000,000	1,000,000 to 10,000,000	100,000 to 1,000,000	100,000 to 1,000,000	1,000 to 10,000	1,000 to 10,000	1,000 to 10,000	Camelids, bison
Chile	100,000 to 1,000,000	1,000,000 to 10,000,000	1,000,000 to 10,000,000	100,000 to 1,000,000	100,000 to 1,000,000	1,000 to 10,000	Not applicable	
Colombia	100,000 to 1,000,000	100,000 to 1,000,000	10,000 to 100,000	1,000 to 10,000	Not applicable	Not applicable	10,000 to 100,000	Water buffalo (Bubalus bubalis)
Costa Rica	100,000 to 1,000,000	100,000 to 1,000,000	1,000 to 10,000	1,000 to 10,000	Not applicable	Not applicable	Not applicable	
Czech Republic	100,000 to 1,000,000	100,000 to 1,000,000	100,000 to 1,000,000	10,000 to 100,000	Not applicable	10,000 to 100,000	Not applicable	
Denmark	1,000,000 to 10,000,000	100,000 to 1,000,000	10,000 to 100,000	1,000 to 10,000	<1,000	<1,000	Unknown	
Ecuador	100,000 to 1,000,000	100,000 to 1,000,000	100,000 to 1,000,000	<1,000	Not applicable	Not applicable	Not applicable	
Finland	100,000 to 1,000,000	100,000 to 1,000,000	100,000 to 1,000,000	1,000 to 10,000	Unknown	<1,000	100,000 to 1,000,000	Other: reindeer. The population of camelids is not available because there is no register; We can only provide the number of the farms. Camelids = llama and alpaca
France	1,000,000 to 10,000,000	>10,000,000	1,000,000 to 10,000,000	1,000,000 to 10,000,000	Not applicable	Not applicable	Not applicable	
Germany	1,000,000 to 10,000,000	1,000,000 to 10,000,000	1,000,000 to 10,000,000	10,000 to 100,000	Unknown	Unknown	Not applicable	
Greece	100,000 to 1,000,000	100,000 to 1,000,000	1,000,000 to 10,000,000	1,000,000 to 10,000,000	Not applicable	Unknown	1,000 to 10,000	Buffalo
Iceland	10,000 to 100,000	1,000 to 10,000	100,000 to 1,000,000	1,000 to 10,000	Not applicable	Not applicable	Not applicable	Wild reindeer 6000-7000

Country	Dairy Cattle	Beef Cattle	Sheep	Goat	Camelid	Deer	Other	Comments
India	> 10,000,000	Not applicable	> 10,000,000	> 10,000,000	100,000 to 1,000,000	Not applicable	> 10,000,000	Other - buffalo
Iran	1,000,000 to 10,000,000	1,000,000 to 10,000,000	> 10,000,000	> 10,000,000	100,000 to 1,000,000	Not applicable	100,000 to 1,000,000	Camelids include Dromedary and Bactrian camels. Other: Buffalo
Israel	100,000 to 1,000,000	10,000 to 100,000	100,000 to 1,000,000	10,000 to 100,000	1,000 to 10,000	Not applicable	Not applicable	
Italy	1,000,000 to 10,000,000	1,000,000 to 10,000,000	1,000,000 to 10,000,000	1,000,000 to 10,000,000	Not applicable	Not applicable	100,000 to 1,000,000	Other: water buffalo
Japan	1,000,000 to 10,000,000	1,000,000 to 10,000,000	10,000 to 100,000	10,000 to 100,000	<1,000	<1,000	<1,000	Other: less than 200 water buffaloes are kept in zoos and small farms. Small number of camelids such as camel, alpaca and llama are kept only in zoos and guest ranches.
Korea	100,000 to 1,000,000	1,000,000 to 10,000,000	<1,000	<1,000	Unknown	<1,000	Unknown	
Lesotho	Unknown	100,000 to 1,000,000	1,000,000 to 10,000,000	1,000,000 to 10,000,000	Not applicable	Not applicable	Not applicable	Cattle farming in Lesotho is based on mixed herds with very few specific dairy herds
Mexico	1,000,000 to 10,000,000	> 10,000,000	1,000,000 to 10,000,000	1,000,000 to 10,000,000	Not applicable	Not applicable	10,000 to 100,000	Cattle include fighting bulls
Nepal	100,000 to 1,000,000	Not applicable	100,000 to 1,000,000	> 10,000,000	Unknown	Not applicable	Not applicable	
Netherlands	1,000,000 to 10,000,000	1,000,000 to 10,000,000	1,000,000 to 10,000,000	100,000 to 1,000,000	<1,000	<1,000	Not applicable	Data from 2016 on cattle population are publicly available at: https://opendata.cbs.nl/statline
New Zealand	1,000,000 to 10,000,000	1,000,000 to 10,000,000	> 10,000,000	10,000 to 100,000	10,000 to 100,000	100,000 to 1,000,000	Unknown	Alpaca (Lama pacos)
Nigeria	1,000,000 to 10,000,000	> 10,000,000	> 10,000,000	> 10,000,000	100,000 to 1,000,000	Not applicable	Not applicable	
Norway	100,000 to 1,000,000	10,000 to 100,000	1,000,000 to 10,000,000	10,000 to 100,000	1,000 to 10,000	1,000 to 10,000	100,000 to 1,000,000	Domesticated and wild reindeer and other wild cervids
Panamá	100,000 to 1,000,000	1,000,000 to 10,000,000	10,000 to 100,000	1,000 to 10,000	Unknown	Unknown	100,000 to 1,000,000	
Poland	1,000,000 to 10,000,000	1,000,000 to 10,000,000	100,000 to 1,000,000	10,000 to 100,000	<1,000	10,000 to 100,000	Unknown	Camelus bactrianus, Lama pacos, Lama glana, Vicugna vicugna individuals in zoos
Republic of Ireland	1,000,000 to 10,000,000	1,000,000 to 10,000,000	1,000,000 to 10,000,000	1,000 to 10,000	Unknown	Unknown	Not applicable	
Slovenia	100,000 to 1,000,000	100,000 to 1,000,000	100,000 to 1,000,000	10,000 to 100,000	<1,000	1,000 to 10,000	Not applicable	
South Africa	1,000,000 to 10,000,000	> 10,000,000	> 10,000,000	1,000,000 to 10,000,000	<1,000	Not applicable	Unknown	Cattle - feedlot
Spain	100,000 to 1,000,000	100,000 to 1,000,000	1,000,000 to 10,000,000	100,000 to 1,000,000	<1,000	1,000 to 10,000	Not applicable	
Swaziland	1,000 to 10,000	100,000 to 1,000,000	10,000 to 100,000	100,000 to 1,000,000	Not applicable	Not applicable	Not applicable	
Sweden	100,000 to 1,000,000	100,000 to 1,000,000	100,000 to 1,000,000	10,000 to 100,000	1,000 to 10,000	1,000 to 10,000	Unknown	Camelids are mainly alpaca

Country	Dairy Cattle	Beef Cattle	Sheep	Goat	Camelid	Deer	Other	Comments
Switzerland	100,000 to 1,000,000	100,000 to 1,000,000	100,000 to 1,000,000	10,000 to 100,000	1,000 to 10,000	10,000 to 100,000	<1,000	Q7-Q11: The numbers only include (animals in) animal holdings with agricultural land. Q7: Dairy cattle = cows kept for commercial milk production; Beef cattle = all cattle except the cows kept for commercial milk production (includes beef cattle, breeding animals, animals in non-commercial animal holdings, zoo animals etc.). Q8: Dairy cattle = farms with commercial milk production (at least one dairy herd). Summer pasture holdings excluded. Besides the dairy herd there may also be beef cattle or other cattle in these farms. Beef cattle = animal holdings without commercial milk production (beef farms, rearing farms, non-commercial animal holdings, zoos etc.) and summer pasture holdings. Camelids: Lama and alpaca. Other: bison.
Thailand	100,000 to 1,000,000	1,000,000 to 10,000,000	10,000 to 100,000	100,000 to 1,000,000	<1,000	1,000 to 10,000	Not applicable	
United Kingdom	1,000,000 to 10,000,000	1,000,000 to 10,000,000	1,000,000 to 10,000,000	1,000 to 10,000	Unknown	Unknown	Not applicable	No census data for other relevant species. Source DEFRA
United States of America	1,000,000 to 10,000,000	> 10,000,000	1,000,000 to 10,000,000	1,000,000 to 10,000,000	100,000 to 1,000,000	100,000 to 1,000,000	Not applicable	Camelids=alpaca and llama. Cattle beef is cow-calf only - feedlots not included
Uruguay	1,000,000 to 10,000,000	> 10,000,000	1,000,000 to 10,000,000	10,000 to 100,000	Not applicable	Not applicable	Not applicable	
Venezuela	1,000,000 to 10,000,000	1,000,000 to 10,000,000	100,000 to 1,000,000	1,000,000 to 10,000,000	Not applicable	Not applicable	100,000 to 1,000,000	Buffalo
Zambia	1,000,000 to 10,000,000	1,000,000 to 10,000,000	100,000 to 1,000,000	1,000,000 to 10,000,000	<1,000	1,000 to 10,000	> 10,000,000	
Zimbabwe	10,000 to 100,000	1,000,000 to 10,000,000	100,000 to 1,000,000	1,000,000 to 10,000,000	Not applicable	Not applicable	Not applicable	

Supplementary Table 2. Notifiability of paratuberculosis in ruminants among 48 countries

**Country**	**Dairy cattle**	**Beef cattle**	**Sheep**	**Goats**	**Camelids**	**Deer-farmed**	**Other**
Argentina	Yes	Yes					
Australia	Yes	Yes	Yes	Yes	Yes	Yes	Yes
Austria	Yes	Yes	Yes	Yes		Yes	
Bangladesh	Yes	Yes					
Belgium							
Bhutan	Yes		Yes	Yes			
Brazil	Yes	Yes	Yes	Yes	Yes	Yes	Buffalo
Canada	Yes	Yes	Yes	Yes	Yes	Yes	Bison
Chile	Yes	Yes	Yes	Yes			
Colombia	Yes	Yes	Yes	Yes			Water buffalo
Costa Rica	Yes	Yes					
Czech Republic							
Denmark							
Ecuador							
Finland	Yes	Yes	Yes	Yes	Yes	Yes	All animals
France							
Germany	Yes	Yes	Yes	Yes	Yes	Yes	
Greece							
Iceland	Yes	Yes	Yes	Yes			
India							
Iran							
Israel	Yes	Yes	Yes	Yes			
Italy	Yes	Yes					Buffalo
Japan	Yes	Yes	Yes	Yes		Yes	Buffalo
Korea	Yes	Yes					
Lesotho	Yes	Yes	Yes	Yes			
Mexico	Yes	Yes	Yes	Yes			
Nepal	Yes		Yes	Yes			
Netherlands							
New Zealand							
Nigeria							
Norway	Yes	Yes	Yes	Yes	Yes	Yes	All species
Panamá	Yes	Yes					
Poland	Yes	Yes	Yes	Yes	Yes	Yes	
Republic of Ireland	Yes	Yes	Yes	Yes		Yes	
Slovenia	Yes	Yes	Yes	Yes			
South Africa	Yes	Yes	Yes	Yes			
Spain	Yes	Yes	Yes	Yes	Yes	Yes	
Swaziland	Yes	Yes	Yes	Yes			
Sweden	Yes	Yes	Yes	Yes	Yes	Yes	All species
Switzerland	Yes	Yes	Yes	Yes	Yes	Yes	Bison
Thailand	Yes	Yes	Yes	Yes	Yes	Yes	
United Kingdom	Yes	Yes	Yes	Yes			
United States of America							
Uruguay							
Venezuela	Yes	Yes					
Zambia	Yes	Yes	Yes	Yes	Yes	Yes	Yes
Zimbabwe	Yes	Yes	Yes	Yes			
Total	35	33	28	28	12	15	10

Supplementary Table 3. Herd-level prevalence by country for each type of ruminant. The definition of prevalence may have varied between countries and between species within countries (proportion of herds with a MAP infection, proportion of herds being test-positive or proportion of herds with animals with clinical signs of paratuberculosis). The tests and selection of tested animals may have varied between countries and between species within countries

Country	Dairy cattle	Beef cattle	Sheep	Goats	Camelids	Deer-farmed	Other sp.	Other-comment
Argentina	10-20%	1-10%	Unknown	<1%	Unknown	1-10%	Unknown	Overall prevalence is 7.9% among 9 regions of Argentina. Beef cattle 7.0 %; Dairy cattle 17.1%
Australia	1-10%	<1%	1-10%	<1%	<1%	Unknown	Not applicable	There are significant regional and sectorial differences in prevalence in Australia
Austria	Unknown	Unknown	Unknown	Unknown	10-20%	Unknown	Unknown	Cattle farms: 19.05%. Prevalence in camelids refers only to new world camelids
Bangladesh	Unknown	Unknown	Unknown	Unknown	Unknown	Unknown	Unknown	
Belgium	>40%	20-40%	Unknown	Unknown	Unknown	Unknown	Unknown	
Bhutan	1-10%	Not applicable	Not applicable	Not applicable	Not applicable	Not applicable	Not applicable	
Brazil	Unknown	Unknown	Unknown	Unknown	Unknown	Unknown	Unknown	Other = Buffalo. There are studies that show percentages of positive animals in certain regions and using different methodologies (see review in Trop Anim Health Prod; DOI 10.1007 / s11250-017-1385-6). There are no data for Brazil as a whole.
Canada	>40%	1-10%	>40%	>40%	Unknown	Unknown	Unknown	Other = Bison
Chile	>40%	Unknown	Unknown	Unknown	Unknown	Unknown	Unknown	
Colombia	1-10%	20-40%	1-10%	<1%	Not applicable	Not applicable	<1%	Other = Water buffalo
Costa Rica	Unknown	Unknown	Unknown	Unknown	Not applicable	Unknown	Unknown	In 1992 a survey with 654 serum samples from the whole country demonstrated a herd-level prevalence of 18.7% and a prevalence of 11.9% among the tested animals
Czech Republic	Unknown	Unknown	Unknown	Unknown	Not applicable	Unknown	Not applicable	Assumed prevalence is higher than 40%
Denmark	>40%	Unknown	Unknown	Unknown	Unknown	Unknown	Unknown	
Ecuador	1-10%	Unknown	Unknown	Unknown	Unknown	Unknown	Unknown	
Finland	Unknown	Unknown	Unknown	Unknown	Unknown	Unknown	Unknown	Other = Reindeer
France	>40%	>40%	1-10%	10-20%	Not applicable	Not applicable	Not applicable	Prevalence is known from Brittany, but prevalence is unknown in most of France
Germany	>40%	Unknown	Unknown	Unknown	Unknown	Unknown	Not applicable	
Greece	10-20%	10-20%	10-20%	20-40%	Unknown	Unknown	Unknown	
Iceland	Unknown	Unknown	Unknown	Unknown	Not applicable	Not applicable	Not applicable	
India	20-40%	Not applicable	20-40%	20-40%	Not applicable	Not applicable	20-40%	Other = Buffalo

Country	Dairy cattle	Beef cattle	Sheep	Goats	Camelids	Deer-farmed	Other sp.	Other-comment
Iran	Unknown	Unknown	Unknown	Unknown	Unknown	Unknown	Not applicable	There are some papers on MAP prevalence in bulk tank milk and faecal samples, but they do not allow for conclusions about herd-level prevalence
Israel	>40%	Unknown	Unknown	Unknown	Not applicable	Not applicable	Not applicable	
Italy	>40%	Unknown	>40%	>40%	Not applicable	Not applicable	>40%	Other = Water buffalo
Japan	1-10%	<1%	<1%	<1%	Unknown	<1%	<1%	For dairy and beef cattle farms, herd-level prevalence was calculated as the number of known infected farms divided by the total number of farms. For goats and sheep, one case had been reported for each of these species in 2015. For deer and water buffalo, no case had ever been reported. Paratuberculosis in camelids is not listed as a notifiable disease in the Act on Domestic Animal Infectious Diseases Control
Korea	1-10%	10-20%	Unknown	Unknown	Unknown	Unknown	Unknown	
Lesotho	Unknown	Unknown	Unknown	Unknown	Not applicable	Not applicable	Not applicable	
Mexico	Unknown	Unknown	Unknown	Unknown	Not applicable	Not applicable	Not applicable	
Nepal	1-10%	Not applicable	1-10%	1-10%	Not applicable	Not applicable	Not applicable	
Netherlands	20-40%	10-20%	Unknown	>40%	Unknown	Unknown	Not applicable	
New Zealand	>40%	>40%	>40%	Unknown	Unknown	>40%	Not applicable	
Nigeria	Unknown	Unknown	Unknown	Unknown	Unknown	Unknown	Unknown	
Norway	<1%	<1%	<1%	<1%	<1%	Unknown	Unknown	Other = domesticated and wild reindeer and other wild cervids
Panamá	20-40%	Unknown	Unknown	Unknown	Not applicable	Not applicable	Not applicable	
Poland	Unknown	Unknown	Unknown	Unknown	Unknown	Unknown	Not applicable	
Republic of Ireland	20-40%	1-10%	Unknown	Unknown	Not applicable	Unknown	Not applicable	
Slovenia	10-20%	1-10%	Unknown	Unknown	Unknown	Unknown	Not applicable	
South Africa	Unknown	Unknown	1-10%	Unknown	Unknown	Not applicable	Not applicable	
Spain	>40%	20-40%	20-40%	>40%	<1%	Unknown	Not applicable	
Swaziland	Unknown	Unknown	Unknown	Unknown	Not applicable	Not applicable	Not applicable	
Sweden	<1%	<1%	Unknown	Unknown	Unknown	Unknown	Unknown	Estimates are based on negative surveillance results and MAP is considered absent. MAP has not been detected in dairy cattle, other ruminant species or wildlife.
Switzerland	1-10%	Unknown	Unknown	Unknown	Unknown	Unknown	Unknown	Other = Bison

Country	Dairy cattle	Beef cattle	Sheep	Goats	Camelids	Deer-farmed	Other sp.	Other-comment
Thailand	<1%	<1%	<1%	<1%	<1%	<1%	Not applicable	
United Kingdom	>40%	20-40%	1-10%	Unknown	Unknown	Unknown	Not applicable	
United States of America	>40%	10-20%	Unknown	1-10%	Unknown	Unknown	Not applicable	
Uruguay	>40%	<1%	Unknown	Unknown	Not applicable	Not applicable	Not applicable	
Venezuela	Unknown	Unknown	Unknown	Unknown	Not applicable	Not applicable	<1%	
Zambia	Unknown	Unknown	Unknown	Unknown	Unknown	Unknown	Unknown	
Zimbabwe	Unknown	Unknown	Unknown	Unknown	Not applicable	Not applicable	Not applicable	

Supplementary Table 4. Within-herd prevalence by country for each type of ruminant. Data are the estimated prevalence of paratuberculosis within known infected herds unless otherwise stated in the comments. The definition of prevalence may have varied between countries and between species within countries (proportion of herds with a MAP infection, proportion of herds being test-positive or proportion of herds with animals with clinical signs of paratuberculosis). The tests and selection of tested animals may have varied between countries and between species within countries

Country	Dairy cattle	Beef cattle	Sheep	Goats	Camelids	Deer-farmed	Other sp.	Comment
Argentina	10-15%	1-5%	Unknown	<1%	Unknown	5-10%	Unknown	
Australia	5-10%	1-5%	10-15%	5-10%	<1%	Unknown	Not applicable	These are estimates of prevalence of infection, not incidence
Austria	Unknown	Unknown	Unknown	Unknown	Unknown	Unknown	Unknown	
Bangladesh	Unknown	Unknown	Unknown	Unknown	Unknown	Unknown	Unknown	
Belgium	1-5%	1-5%	Unknown	Unknown	Unknown	Unknown	Unknown	
Bhutan	5-10%	Not applicable	Not applicable	Not applicable	Not applicable	Not applicable	Not applicable	
Brazil	Unknown	Unknown	Unknown	Unknown	Not applicable	Not applicable	Unknown	Other = Buffalo
Canada	10-15%	5-10%	>15%	>15%	Unknown	Unknown	Unknown	
Chile	10-15%	Unknown	Unknown	Unknown	Unknown	Unknown	Unknown	
Colombia	>15%	>15%	5-10%	Unknown	Not applicable	Not applicable	Unknown	
Costa Rica	Unknown	Unknown	Unknown	Unknown	Not applicable	Unknown	Unknown	
Czech Republic	Unknown	Unknown	Unknown	Unknown	Not applicable	Unknown	Not applicable	There are herds completely free of paratuberculosis and herds with up to 10% within-herd prevalence. Systematic and detailed information is lacking.
Denmark	5-10%	Unknown	Unknown	Unknown	Unknown	Unknown	Unknown	
Ecuador	1-5%	Unknown	Unknown	Unknown	Unknown	Unknown	Not applicable	Prevalence of ELISA positives known to be more than 25% in one herd
Finland	Unknown	Unknown	Unknown	Unknown	Unknown	Unknown	Unknown	Other = Reindeer
France	5-10%	5-10%	5-10%	5-10%	Not applicable	Not applicable	Not applicable	Great variation between regions, herds and farms. Prevalence is known from Brittany, but prevalence is unknown in most of France
Germany	5-10%	1-5%	Unknown	Unknown	Unknown	Unknown	Not applicable	This is true for the majority of herds (85%). 15% of the herds have a higher prevalence. Different herds have different prevalences. There is large variation between regions and herds; within-herd prevalence ranges from 0.1% to > 50%
Greece	5-10%	5-10%	10-15%	>15%	Unknown	Unknown	Unknown	
Iceland	Unknown	Unknown	Unknown	Unknown	Not applicable	Not applicable	Not applicable	

Country	Dairy cattle	Beef cattle	Sheep	Goats	Camelids	Deer-farmed	Other sp.	Comment
India	>15%	Not applicable	>15%	>15%	Not applicable	Not applicable	>15%	Other = Buffalo
Iran	Unknown	Unknown	Unknown	Unknown	Unknown	Unknown	Unknown	
Israel	1-5%	1-5%	Unknown	Unknown	Not applicable	Not applicable	Not applicable	In dairy herds the prevalence varies from 0 to 17%
Italy	1-5%	Unknown	1-5%	5-10%	Not applicable	Not applicable	1-5%	Other = water buffalo
Japan	Not applicable	Not applicable	Not applicable	Not applicable	Not applicable	Not applicable	Not applicable	As a result of test-and-cull strategy applied, all detected cattle are immediately removed from farms. Therefore, estimation of (apparent) within-herd prevalence is not meaningful although it is believed to be very low
Korea	10-15%	>15%	Unknown	Unknown	Unknown	Unknown	Unknown	
Lesotho	Unknown	Unknown	Unknown	Unknown	Not applicable	Not applicable	Not applicable	
Mexico	10-15%	1-5%	10-15%	10-15%	Not applicable	Not applicable	Unknown	Cattle include fighting bulls
Nepal	Unknown	Unknown	Unknown	Unknown	Unknown	Unknown	Unknown	
Netherlands	10-15%	Unknown	Unknown	1-5%	Unknown	Unknown	Not applicable	Dairy cattle: true animal level prevalence of infection across all herds (both infected and uninfected herds) was estimated at 0.058 (95% CI: 0.042, 0.076). Van Schaik et al 2003, Prev Vet Med 90, 281-285.
New Zealand	10-15%	<1%	<1%	Unknown	Unknown	>15%	Not applicable	For beef cattle and sheep we report annual incidence of clinical disease instead of prevalence; affected animals are culled quickly.
Nigeria	Unknown	Unknown	Unknown	Unknown	Unknown	Unknown	Unknown	
Norway	Not applicable	Not applicable	Not applicable	Not applicable	Not applicable	Not applicable	Not applicable	Currently there are no known infected herds
Panamá	>15%	Not applicable	Not applicable	Not applicable	Not applicable	Not applicable	Not applicable	
Poland	Unknown	Unknown	Unknown	Unknown	Unknown	Unknown	Not applicable	
Republic of Ireland	1-5%	1-5%	Unknown	Unknown	Not applicable	Unknown	Not applicable	
Slovenia	5-10%	5-10%	Not applicable	Not applicable	Not applicable	Not applicable	Not applicable	
South Africa	Unknown	Unknown	Unknown	Unknown	Unknown	Not applicable	Not applicable	
Spain	10-15%	1-5%	1-5%	1-5%	Unknown	Unknown	Unknown	
Swaziland	Unknown	Unknown	Unknown	Unknown	Not applicable	Not applicable	Not applicable	
Sweden	Unknown	Unknown	Unknown	Unknown	Unknown	Unknown	Unknown	There are currently no known infected herds
Switzerland	5-10%	5-10%	Unknown	Unknown	Unknown	Unknown	Unknown	Other = bison

Country	Dairy cattle	Beef cattle	Sheep	Goats	Camelids	Deer-farmed	Other sp.	Comment
Thailand	<1%	<1%	<1%	<1%	<1%	<1%	Not applicable	
United Kingdom	Unknown	Unknown	Unknown	Unknown	Unknown	Unknown	Not applicable	
United States of America	5-10%	<1%	Unknown	Unknown	Unknown	Unknown	Not applicable	
Uruguay	5-10%	<1%	Unknown	Unknown	Not applicable	Not applicable	Not applicable	
Venezuela	Unknown	Unknown	Unknown	Unknown	Not applicable	Not applicable	Not applicable	
Zambia	Unknown	Unknown	Unknown	Unknown	Unknown	Unknown	Unknown	
Zimbabwe	Unknown	Unknown	Unknown	Unknown	Not applicable	Not applicable	Not applicable	

Supplementary Table 5. Free living wildlife species with MAP infection

Common name	Scientific name	Country
Alpaca	*Lama pacos*	Chile
Alpine ibex	*Capra ibex*	Italy
American bison	*Bison bison*	United States of America
Badger	*Meles meles*	United Kingdom (Scotland)
Bighorn sheep	*Ovis canadensis*	Canada, United States of America
Bison	*Bison sp.*	India
Blackbuck	*Antilope cervicapra*	India
Blue bull (nilgai)	*Boselaphus tragocamelus*	India
Brushtail possum	*Trichosurus vulpecula*	New Zealand
Caribou	*Rangifer sp.*	Canada
Cat	*Felis catus*	New Zealand, United States of America
Chamois	*Rupicapra rupicapra*	Czech Republic
Crow	*Corvus corone*	United Kingdom (Scotland)
Sika deer	*Cervus nippon*	Korea, United Kingdom (Scotland)
White tailed deer	*Odocoileus virginianus*	United States of America
Key deer	*Odocoileus virginianus clavium*	United States of America
Elk	*Cervus elaphus canadensis*	Canada
European hedgehogs	*Erinaceus europaeus*	New Zealand
European rabbit	*Oryctolagus cuniculus*	India, Scotland, United Kingdom, New Zealand
Fallow deer	*Dama dama*	Czech Republic, United Kingdom
Fox	*Vulpes vulpes*	Greece, United Kingdom (Scotland)
Guanaco	*Lama guanicoe*	Argentina, Chile
Brown hare	*Lepus europaeus*	Argentina, Chile, Greece, United Kingdom
Huemul	*Hippocamelus bisulcus*	Chile
Indian hog deer	*Hyelaphus porcinus*	India
Jackdaw	*Corvus monedula*	United Kingdom (Scotland)
Jungle cat	*Felis chaus*	India
Mithun (gayal)	*Bos frontalis*	India
Crab-eating macaque	(*Macaca Fascicularis*)	India
Mouflon	*Ovis musimon*	Czech Republic, Korea
Mountain sheep - shapo	*Ovis orientalis vignei*	India
Paradise shelduck	*Tadorna variegata*	New Zealand
Pudu	*Pudu puda*	Chile

Common name	Scientific name	Country
Pygmy goat	*Capra aegagrus hircus*	United States of America
Rat	*Rattus norvegicus*	United Kingdom (Scotland)
Red deer	*Cervus elaphus*	Czech Republic, Denmark, Italy, Germany, United Kingdom
House mouse	*Mus musculus*	Greece
Black rat	*Rattus rattus*	Czech Republic, Greece
Roe deer	*Capreolus capreolus*	Czech Republic, Denmark, Italy, Spain, United Kingdom
Rook	*Corvus frugilegus*	United Kingdom (Scotland)
Rocky Mountain goat	*Oreamnos americanus*	United States of America
Stoat	*Mustela erminea*	United Kingdom (Scotland)
Striped hyena	*Hyaena hyaena*	India
Tammar wallaby	*Macropus eugenii decres*	Australia
Tule elk	*Cervus canadensis nannodes*	United States of America
Western grey kangaroo	*Macropus fuliginosus fuliginosus*	Australia
Weasel	*Mustela nivalis*	United Kingdom (Scotland)
Wild boar	*Sus scrofa*	Czech Republic, Korea, Italy
Wood mouse	*Apodemus sylvaticus*	United Kingdom (Scotland)

Supplementary Table 6. Countries with control programs for paratuberculosis. The start date is the earliest commencement date for any component of a control program in any species

Country	End before 2012	2012 to 2018	Long term[1]	Start date[3]	End date	New Program to start after 2018
Australia		Yes	Yes	1996	2018	Yes
Austria		Yes	Yes	2006		
Belgium		Yes	Yes	2006		
Canada		Yes	Yes	2007	2018	Yes
Chile						Yes
Denmark		Yes	Yes	2006		Yes
France		Yes	Yes	1980		
Germany	Yes	Yes	Yes	2003		
Iceland		Yes	Yes	1962		Yes
Italy		Yes	Yes	2014		
Japan		Yes	Yes	1971		
Korea		Yes	Yes	1980		
Netherlands	Yes	Yes	Yes	1942		
New Zealand		Yes	Yes	2009		
Norway		Yes	Yes	1978		
Republic of Ireland	Yes	Yes		2013		
South Africa		Yes	Yes	1997		
Slovenia						Yes
Spain		Yes	Yes	2004		
Sweden**		Yes	Yes	1998		
Switzerland		Yes		2015		
Thailand		Yes	Yes	1988		
United Kingdom		Yes	Yes	1998		Yes
United States of America		Yes	Yes	2002		
Total	3	22	20			7

[1] Commenced before 2012 and continues after 2018 [2] start date of programs running between 2012-2018 [3] considered to be a surveillance program after 2008

Supplementary Table 7. Commencement and major chronological events in paratuberculosis control programs in 22 countries

Country	Start	Major chronological events
Australia	1996	1996 first Market Assurance Program. 1999 first national program in sheep; 2000 first national program in cattle; 2012 latest plan for sheep; 2016 change to plan for cattle and a National Johne's Disease Project in place.
Austria	2006	2006 legislation came into force; no significant changes since.
Belgium	2006	2011 change in the classification of risk levels; mandatory testing of only dairy cattle in mixed herds (before that all animals had to be tested).
Canada	2007	In Alberta, in 2012 the Herd Status program started.<br>In Ontario, Phase I began in 2010 with subsidized testing of cows with milk/serum ELISA and removal of high titre positive cows with partial compensation; Phase 2 began in 2013 with voluntary participation in risk assessment and/or testing at owner's expense.<br>In Quebec from 2014 there was minimal effort to recruit more herds; in 2017 the program officially ended.<br>In Atlantic provinces in 2016 there was a move from environmental culture to environmental direct PCR.
Denmark	2006	2008 changed from in-house test to ID Vet ELISA; 2011 option to have a true prevalence and probability of freedom estimate calculated for the individual farm, along with categorisation of the herd
France	1980	At national level started in 1999; 2006 review of the manual. In Brittany, started in 1980; systematic screening of herds started in 2011 and 2018. Local programs now coordinated regionally for western France (by GDS Grand Ouest).
Germany	2003	At national level, in 2005 guidelines for the control of paratuberculosis in ruminant herds were published; in 2014 they were abolished and national guidelines for hygienic measures in ruminant herds containing a chapter for paratuberculosis control were published.<br>In Thuringia, in 2003 voluntary control started with biannual serological testing of each cow and confirmation by fecal culture, selection of FC positive cows; in 2007 annual herd testing by individual fecal culture testing of each adult cow and risk assessment; in 2015 adoption of the guidelines of the federal ministry, and additionally farmers may enroll in eradication of MAP at herd level.<br>In Lower Saxony, in 2016 voluntary animal testing and risk assessment began; in 2017 a compulsory animal testing and risk assessment by- law including testing of replacement stock began.
Iceland	1949	Paratuberculosis first diagnosed in sheep in 1939, cattle 1945 and goats 1969. 1949-1951 stamping out in two seriously affected areas; quarantine areas. 1962 vaccination of sheep started; 1966 compulsory vaccination of sheep in affected areas.
Italy	2014	National guidelines were approved in 2013; 2014 regions started to adopt national guidelines; 2016 the last region adopted national guidelines.
Japan	1971	1971 the Act on Domestic Animal Infectious Diseases Control was amended to add paratuberculosis to the list of its target diseases and enforcement of culling of all cases with compensation; 1997 legislation amended to start active surveillance for paratuberculosis; 1998 national surveillance program started in cattle; all dairy and beef breeding cattle targeted for serological surveillance at intervals <5 years; all detected animals slaughtered with compensation; for detected farms, five negative tests within 3 years required to regain free status; 2003 the Food Sanitation Act and relevant legislation were amended and processing and retail sale of carcasses and milk from animals diagnosed as infected with designated diseases including paratuberculosis was prohibited; 2006 the Guidelines of National Control Program for Paratuberculosis established by MAFF; 2013 serological tests (ELISA) were replaced with real time PCR.

Country	Start	Major chronological events
Korea	1980	JD is regulated as a 2nd grade legal communicable disease. Nationwide surveillance is carried out by serological testing of 20,000 head every year. Movement restrictions are applied to positive farms until the positive cattle have been eliminated from the farm. The positive case is announced in the KAHIS system.
Netherlands	1942	1942-1984: test-and-cull based on Johnin skin test and, from 1954, complement fixation test; 1984-1994: vaccination program; 1996 to present: Intensive Paratuberculosis Programme; 2006 to present: Milk Quality Assurance Programme; 2014 to present: Paratuberculosis Programme for Sheep and Goats.
New Zealand	2009	Deer: 2006 Johne's Management Ltd founded; 2009 abattoir surveillance started; 2010 guidelines and training and accreditation of vets, Focus Farm program launched and Farmer Manual sent to all deer farmers<br>Cattle dairy: 2014 dairy guidelines published, voluntary uptake<br>Sheep: 2014 vaccination recommended for fine-wool breeds, voluntary uptake; 2016 research on economic benefits completed and propagated
Norway	1978	Notifiable since 1907. Vaccination of goats was applied optionally from 1967. However, compulsory vaccination of all goat herds commenced in 1978 and this is considered to be the start of the control programme. 1978-2014 compulsory vaccination in goats, 1996 active surveillance started for cattle, 2000 camelids included, 2001 goats included, 2002 sheep included, 2001-2014 eradication programme in goats, 2006 goat bulk milk surveillance started.
Republic of Ireland	2013	2017 added knowledge exchange activities and enhanced communications; included pathways for positive and negative herds. 2013-2016, 2017-2022
South Africa	1997	N/A
Spain	2004	Basque Country: 2005 four farms were enrolled in vaccination program; 2018 20 farms enrolled in the program.
Sweden	1998	2005 survey in dairy cattle; 2006 and 2011 included targeted sampling of imported animals and herds with imported animals; 2012-2013 included targeted sampling of thin cows in dairy herds. Since 2008 the program has been a surveillance program.
Switzerland	2015	2015 paratuberculosis newly listed as an “animal disease to control” in the Ordonnance on Epizootic Diseases and defined control measures made compulsory at national level
Thailand	1988	1988 applied to dairy cattle; 1992 applied to livestock; <1992 test and slaughter; 1999 identify free farm; 2007 made a notifiable disease in Epidemic Act 2007.
United Kingdom	1998	2013 risk level assurance replaced herd freedom status.
United States of America	2002	2010, USDA revised the program standards decreasing the complexity of the certification program by eliminating the separation of the test negative and test positive classifications and to allow greater flexibility in testing options available to producers.

Supplementary Table 8. Comparison of countries with or without a control program for paratuberculosis and for other diseases. The values in cells represent the numbers of countries. P-values are based on Fisher's exact tests.

**Other disease**	**Control program for other disease**	**Control program for paratuberculosis**			**P-value**
		**Yes**	**No**	**Total**	
Bovine					0.20
	Yes	19	19	38	
	No	1	5	6	
	Total	20	24	44	
Bovine brucellosis					0.61
	Yes	18	21	39	
	No	1	3	4	
	Total	19	24	43	
Rinderpest					0.43
	Yes	5	4	9	
	No	8	14	22	
	Total	13	18	31	
Foot-and-mouth					0.71
	Yes	14	18	32	
	No	3	6	9	
	Total	17	24	41	
Other diseases					0.49
	Yes	21	21	42	
	No	0	2	2	
	Total	21	23	44	

Supplementary Table 9. The geographic organisation of control programs in each of the livestock species in the 22 countries that had control programs for paratuberculosis in the period 2012-2018. DC dairy cattle; BC beef cattle; S sheep; G goat; C camelid; D deer-farmed; WB water buffalo; B bison

**Country**	**Type of control program**			**Species is not present or not subject to a control program**
	**Regional control program**	**Single national control program**	**More than one national control program**	
Australia		DC BC S G C		D
Austria		DC BC S G D B		C
Belgium	DC BC			S G C D
Canada	DC			BC S G C D
Denmark		DC		BC S G C D
France	DC BC S G			C D
Germany	DC BC	S G		C D
Iceland		DC BC S G		C D
Italy		DC BC WB		S G C D
Japan		DC BC S G D WB		C
Korea		DC BC		S G C D
Netherlands		S G	DC BC	C D
New Zealand		DC S D		BC G C
Norway		DC BC S G C D		
Republic of Ireland		DC BC		S G C D
South Africa		DC BC S G		C D
Spain	DC BC S			G C D
Sweden[1]		DC BC S G C D		
Switzerland		DC BC S G C D WB B		
Thailand		DC BC S G C D		
United Kingdom		BC	DC	S G C D
United States of America		DC BC		S G C D
Total number of countries	5	18	2	

[1] Surveillance program since 2008

Supplementary Table 10. Sources of funding for leadership activities within paratuberculoisis control programs in 22 countries

Country	Government	Farmer_Org	Farmer	Processing	Other	Comments
Australia	25%	0%	0%	0%	75%	Levies on sales of livestock and their products nationally fund national management program. State control programs (SA, NT and WA) are funded by state level levies on farmers.
Austria	100%	0%	0%	0%	0%	
Belgium	0%	0-50%	0%	0-50%	100%	ARSIA
Canada	25-100%	0-75%	0%	0%	0%	
Denmark	0%	100%	0%	0%	0%	
France	0%	100%	0%	0%	0%	
Germany	0-50%	0-50%	0%	0%	0%	The programs are funded by animal disease funds which themselves are equally funded by the governments and farmers of the federal states concerned.
Iceland	100%	0%	0%	0%	0%	
Italy	100%	0%	0%	0%	0%	Certification costs and validation of biosecurity plans are covered by the public health system
Japan	100%	0%	0%	0%	0%	
Korea	75%	25%	0%	0%	0%	
Netherlands	0%	0%	100%	0%	0%	
New Zealand	0%	100%	0%	0%	0%	Deer only; in Cattle dairy and sheep there is no leadership and private vets respond to farmer demand
Norway	100%	0%	0%	0%	0%	Government provides complete funding of a complete surveillance and control program. Additional testing of goat bulk milk as an industry initiative is funded by the dairy industry. "Healthier goats project" 2001-2014 was a joint effort by authorities and industry.
Republic of Ireland	50%	0%	0%	50%	0%	The government contributes 50% which is matched by the milk processing sector. Farmers contribute in-kind through testing costs which are partially subsidised
South Africa	100%	0%	0%	0%	0%	The control program is limited to quarantine measures and slaughter of infected animals (without compensation), hence the inputs and costs are minimal with regard to leadership and coordination.
Spain	75-100%	0-25%	0%	0%	0%	
Sweden	75%	0%	25%	0%	0%	
Switzerland	100%	0%	0%	0%	0%	

Country	Government	Farmer_Org	Farmer	Processing	Other	Comments
Thailand	50%	0%	100%	0%	0%	
United Kingdom	0%	0%	100%	0%	0%	
United States of America	100%	0%	0%	0%	0%	Most program management activities are funded at a state level. USDA provided ~$166 million since 2002 for infrastructure and research; current federal funding is for laboratory proficiency testing, vaccine and diagnostic kit licensing and education.
No. of countries with component funding	16	8	3	2	3	

Supplementary Table 11. Sources of funding for operational activities within paratuberculosis control programs in 22 countries

Country	Government	Farmer Org	Farmer	Processing	Other	Comments
Australia	25%	0%	25%	0%	50%	National and state levies for half of the operational activities but also significant costs for farmers (e.g. testing on farm) and governments providing technical and communication support
Austria	100%	0%	0%	0%	0%	
Belgium	0%	50-75%	25%	0-50%	0%	
Canada	25-50%	0-75%	0-50%	0%	0%	
Denmark	0%	0%	100%	0%	0%	
France	0%	0-75%	0-25%	0%	0%	Funded by farmer organisation and farmer, but the proportion depends on the component. Eg screening herds: farmer organisation 100%
Germany	0-25%	0-50%	0-50%	0%	0-75%	The proportion of funding by the different stakeholders differs in the federal states
Iceland	0%	0%	100%	0%	0%	Farmers pay for vaccination.
Italy	0%	0%	100%	0%	0%	Sampling and diagnostic tests are payed by the farmers except for confirmation of clinical cases which is paid by the Government
Japan	100%	0%	0%	0%	0%	
Korea	75%	25%	0%	0%	0%	
Netherlands	0%	0%	100%	0%	0%	
New Zealand	0%	25%	75-100%	0%	0%	Deer: total management costs for the program operated by JML are born by a levy on venison sales; all on-farm cost are paid by farmers (monitoring, testing, culling/replacement, biosecurity measures). Cattle dairy and sheep: 100% farmer
Norway	100%	0%	0%	0%	0%	Government provides complete funding of a complete surveillance and control program. Additional testing of goat bulk milk as an industry initiative is funded by the dairy industry. "Healthier goats project" 2001-2014 was a joint effort by authorities and industry.
Republic of Ireland	50%	0%	25%	25%	0%	The government contributes 50% which is matched by the milk processing sector. However, farmers contribute in-kind through testing costs which are partially subsidised. The government contribution is to reduce annually over time.
South Africa	25%	0%	75%	0%	0%	
Spain	75-100%	0-25%	0-25%	0%	0%	

Country	Government	Farmer Org	Farmer	Processing	Other	Comments
Sweden	75%	0%	25%	0%	0%	
Switzerland	100%	0%	0%	0%	0%	
Thailand	50%	0%	50%	0%	0%	
United Kingdom	0%	0%	100%	0%	0%	
United States of America	0%	0%	100%	0%	0%	Farmers now have primary responsibility to fund the program costs (testing, animal identification, veterinary cost)
No. of countries with component funding	13	7	17	2	2	

Supplementary Table 12. Examples of incentives and penalties for participation in paratuberculosis control programs

Country	Compulsory	Incentive or penalty
Australia	Yes	There are no national restrictions, but some states have restrictions on which animals may enter. Market Assurance Programs are available for producers to demonstrate assurance and receive higher prices for their animals although this has not been consistently realised.
Belgium	No	A part of the testing cost of analysis is refunded to participating farmers.
Canada	No	Funding a vet to do the Risk Assessment and Management Program; free shipping and testing of environmental samples and individual animal milk/serum ELISA testing and removal of high titre positive animals.
France	No	There are recommendations to buy animals from low risk herds.
Germany	Yes	Non-compliance with the MAP-ordinance is an offence and penalties apply; farmers must repay funding which was received for MAP-control; large farms advertise their products with direct reference to their non-suspect status; an increasing number of farmers who buy replacement heifers ask for non-suspect herds and animals; there is higher demand for breeding stock from farms with "non-suspect for paratuberculosis" status.
Italy	No	No incentives. There are restrictions: milk produced by farms with clinical cases cannot be exported to some extra-EU countries that require specific warranties; test positive animals cannot be sold to other farms, but only sent to a slaughterhouse.
Netherlands	No	Dairy cattle herds delivering milk to milk processors must participate in the Milk Quality Assurance Program or the Intensive Paratuberculosis Programme; all test-positive cattle have to be removed from the herd. There are no incentives or restrictions for beef cattle, sheep and goats.
Norway	Yes	Sampling and testing free of charge for the owner; restrictions imposed on movement of animals in infected herds; dairy industry does not collect goat milk from infected herds.
Republic of Ireland	No	Subsidies for testing, funding of VRAMP and confirmatory PCR/Faecal culture.
Spain	No	Cost of testing
Sweden	No	Sweden is free from paratuberculosis; to be a member of the control program is to be considered to be high quality certification. it is difficult to assess how this program status influences the price of animals.
Switzerland	Yes	The affected farm is under official restriction; animal traffic to and from the farm is forbidden except directly to the slaughterhouse; milk of infected or suspect animals is discarded. Officially mandated sampling and testing is free of charge for the owner. Compensation is paid for animals that have to be culled.
Thailand	Yes	Annual testing is free of charge.
United Kingdom	No	Higher prices or ability to sell pedigree stock with low herd risk level. Some milk purchasers impose an absolute requirement for farmers to engage in the control program.

Supplementary Table 13. Sources for control program manuals in 22 countries with a control program for paratuberculosis in 2012 – 2018

Country	Source
Australia	https://www.animalhealthaustralia.com.au/what-we-do/endemic-disease/johnes-disease/national-johnes-disease-project/ and https://www.animalhealthaustralia.com.au/wp-content/uploads/2016/07/JD-in-cattle-definitions-and-guidelines_final_Apr-2017.pdf
Austria	Legislation: Verordnung der Bundesministerin für Gesundheit und Frauen über ein Überwachungsprogramm zur Bekämpfung der klinischen Paratuberkulose bei Wiederkäuern (Paratuberkulose – Verordnung) StF: BGBl. II Nr. 48/2006
Belgium	http://www.dgz.be/programma/programma-paratuberculose-voor-melkveehouders http://www.arsia.be/?page_id=91. http://www.arsia.be/?page_id=7790#
Canada	Under revision; Risk Assessment and Management Plan and Manual for the dairy program: http://www.johnes.ca/forms.htm Fact sheets: http://www.johnes.ca/johnes%20information.htm
Denmark	www.paratuberkulose.dk or direct link: https://www.landbrugsinfo.dk/Kvaeg/Sundhed-og-dyrevelfaerd/Sider/Raadgivermanual__Paratuberkulose_og_Salm.aspx
France	Not available
Germany	http://www.ndstsk.de/index.php?bereich=3&topic_id=948&akk=3&akv= http://www.tknds.de/cms_tknds/index.php?page=339; Empfehlungen des Bundesministeriums für Ernährung und Landwirtschaft für hygienische Anforderungen an das Halten von Wiederkäuern. BAnz AT 01.08.2014 B1; http://www.thueringen.de/mam/th7/tmsfg/veterinaerwesen/tiergesundheit/programm_zur_bekampfung_der_paratuberkulose.pdf
Iceland	http://www.mast.is/dyraheilbrigdi/sjukdomar/garnaveiki/ http://www.mast.is/library/Listar/Listi-garnaveiki-2008-2018.pdf http://www.mast.is/Uploads/document/Skyrslur/Garnaveiki_ahaettugreining_lokaskyrsla.pdf
Italy	http://www.izsler.it/izs_bs/allegati/443/Allegato%201%20A_MANUALE_latte_Rev_1.pdf http://www.izsler.it/izs_bs/allegati/443/Allegato%201_B_MANUALE_carne_Rev_1.pdf
Japan	https://www.naro.affrc.go.jp/niah/disease/files/boeki_taisaku_yoko_130401.pdf (in Japanese)
Korea	http://www.law.go.kr/lsSc.do?menuId=0&p1=&subMenu=1&nwYn=1&query=%EA%B0%80%EC%B6%95%EC%A0%84%EC%97%BC%EB%B3%91%EC%98%88%EB%B0%A9%EB%B2%95&x=0&y=0#undefined
Netherlands	https://www.gddiergezondheid.nl/formulieren/reglementen
New Zealand	Deer: https://deernz.org/sites/dinz/files/farmers_Johnes%20manual.pdf Dairy: https://static1.squarespace.com/static/57857740cd0f68284f643870/t/57e06eee440243a762e4106a/1474326345486/report+1-3+Johnes+disease+booklet+v1.pdf https://www.jdrc.co.nz/resources/
Norway	Norwegian food safety authority's guidelines for control of paratuberculosis. In Norwegian: https://www.mattilsynet.no/dyr_og_dyrehold/dyrehelse/dyresykdommer/paratuberkulose/retningslinjer_for_bekjemping_av_paratuberkolose.18128/binary/Retningslinjer%20for%20bekjemping%20av%20Paratuberkolose

Country	Source
	Case definition and diagnostic methods based on OIE manual<br>Kampen AH, Hermansen L, Agdestein A, Valheim M, and Er C. 2018. The surveillance programme for paratuberculosis in Norway 2017. Surveillance programmes for terrestrial and aquatic animals in Norway. Annual report 2017. Oslo: Norwegian Veterinary Institute.
Republic of Ireland	http://animalhealthireland.ie/wp-content/uploads/2015/08/20171117-JD-Technical-Manual-FINAL.pdf
South Africa	Animal Diseases Act (Reguation254):<br>http://www.nda.agric.za/vetweb/Legislation/GovGezet/Gov%20Gaz%20No%2017790%20R254%206%20Feb%201997.pdf
Spain	Some regions have information included in the Official bulletin. For the government of Galicia:<br>https://www.xunta.gal/dog/Publicados/2018/20180117/AnuncioG0426-271217-0003_es.html
Sweden	Available on request from the Board of Agriculture or Gård & Djurhälsan
Switzerland	https://www.blv.admin.ch/blv/de/home/tiere/tierseuchen/uebersicht-seuchen/alle-tierseuchen/paratuberkulose.html
Thailand	Manual of Animal Health, Department of Livestock Development (DLD) - NIAH Standard Diagnostic Manual, DLD
United Kingdom	http://www.checs.co.uk/wp-content/uploads/2017/09/CHeCS-Technical-Document-2017-Final-Version-for-publication-2.pdf
United States of America	https://www.aphis.usda.gov/animal_health/animal_diseases/johnes/downloads/johnes-ups.pdf

N/A, not available

Supplementary Table 14. Sectorial sources of active stakeholder support by country for paratuberculosis control programs in 22 countries

Country	Government	Industry-milk	Industry-meat	Industry-livestock trading	Farmer organization	Veterinary organization	Private veterinarians	Food processing industry	Individual farmers	Total no. stakeholders
Australia	Yes	Yes	Yes	Yes	Yes	Yes	Yes		Yes	8
Austria	Yes	Yes	Yes	Yes	Yes	Yes				6
Belgium	Yes	Yes			Yes	Yes	Yes		Yes	6
Canada	Yes	Yes	Yes	Yes	Yes	Yes	Yes	Yes	Yes	9
Denmark		Yes			Yes		Yes		Yes	4
France					Yes	Yes	Yes			3
Germany	Yes	Yes		Yes	Yes	Yes	Yes		Yes	7
Iceland	Yes	Yes	Yes	Yes	Yes	Yes	Yes	Yes	Yes	9
Italy	Yes	Yes			Yes	Yes	Yes	Yes	Yes	7
Japan	Yes									1
Korea	Yes	Yes	Yes	Yes	Yes					5
Netherlands	Yes	Yes			Yes	Yes	Yes		Yes	6
New Zealand				Yes	Yes	Yes	Yes		Yes	5
Norway	Yes	Yes	Yes		Yes	Yes	Yes	Yes	Yes	8
Republic of Ireland	Yes	Yes			Yes	Yes	Yes	Yes	Yes	7
South Africa			Yes							1
Spain	Yes				Yes	Yes	Yes		Yes	5
Sweden	Yes	Yes	Yes		Yes	Yes	Yes			6
Switzerland	Yes	Yes			Yes			Yes		4
Thailand	Yes					Yes	Yes		Yes	4
United Kingdom	Yes	Yes	Yes		Yes	Yes	Yes	Yes	Yes	8
United States of America		Yes								1
