# Supplementary material for: Control of paratuberculosis: who, why and how. A review of 48 countries
Source: BMC Vet Res. 2019 Jun 13;15:198. doi: 10.1186/s12917-019-1943-4 (PMC6567393; doi:10.1186/s12917-019-1943-4)

Supplementary Figure 1. Number of farms among 48 countries, 26 without a control program for paratuberculosis and 22 with a control program for paratuberculosis. The box represents the median, 25th and 75th percentiles and outlying countries as individual data points

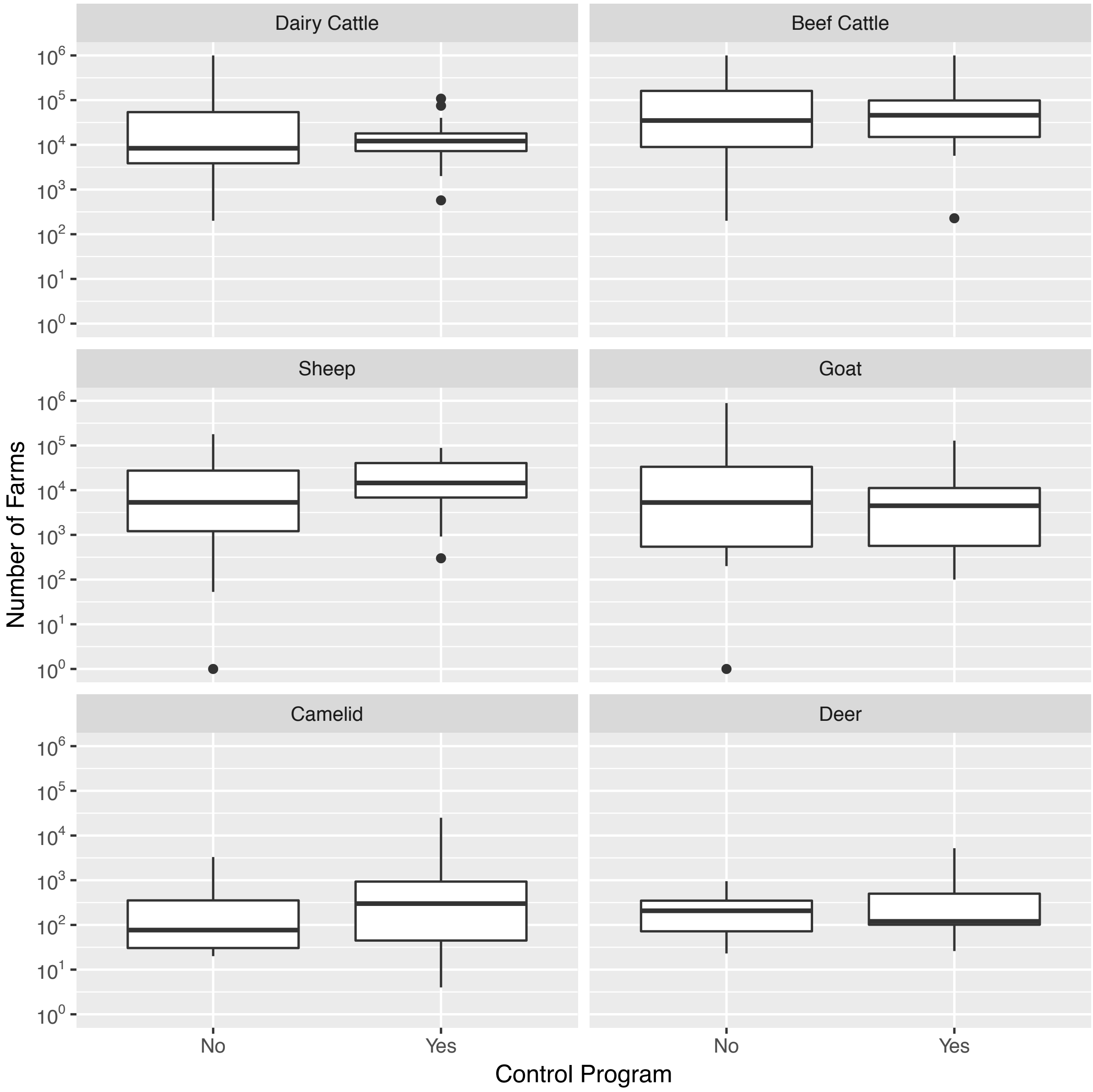

Supplementary Figure 2. Average herd size among 48 countries, 26 without a control program for paratuberculosis and 22 with a control program for paratuberculosis. The box represents the median, 25th and 75th percentiles and outlying countries as individual data points

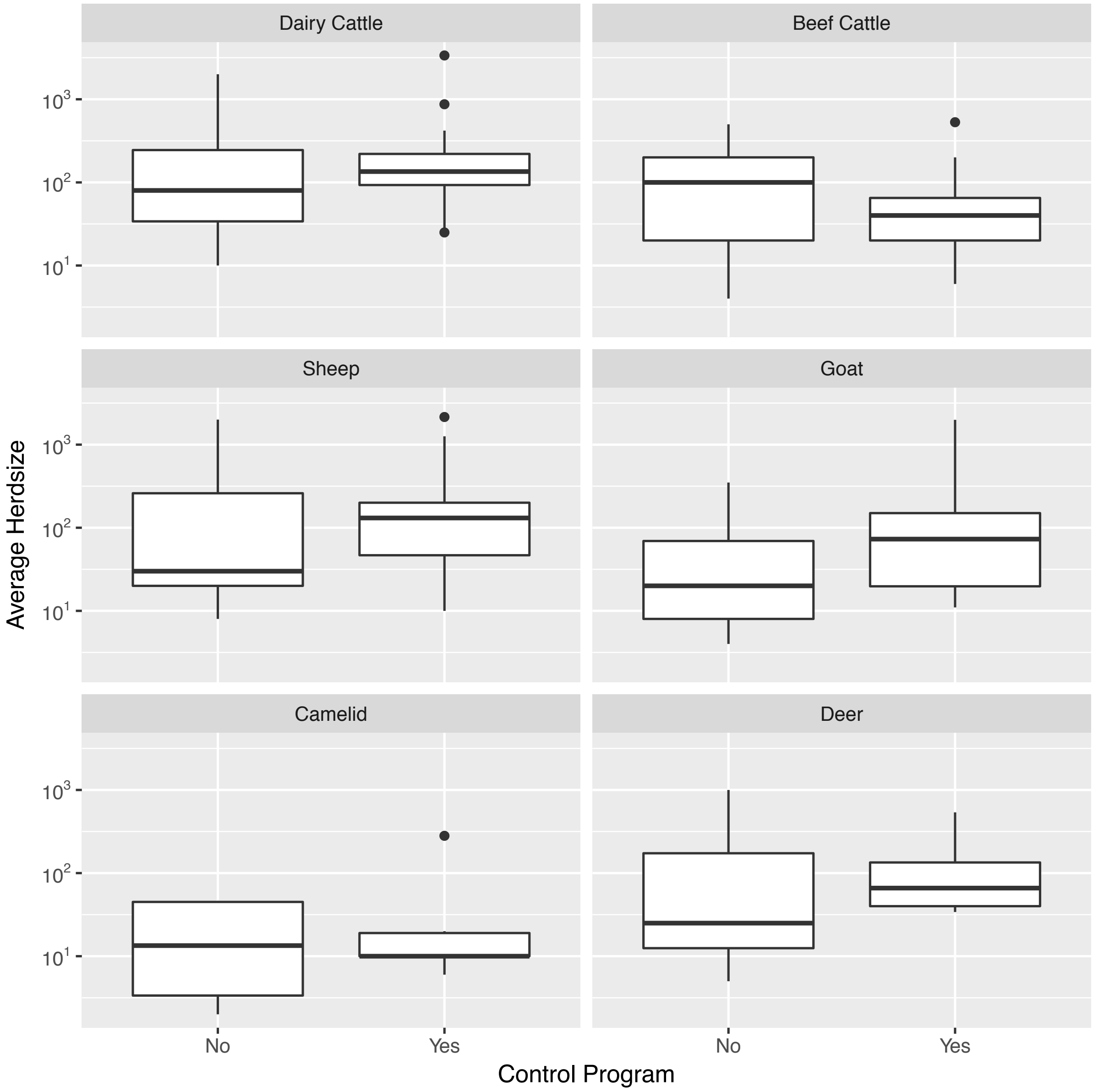

Supplementary Figure 3. Minimum herd size among 48 countries, 26 without a control program for paratuberculosis and 22 with a control program for paratuberculosis. The box represents the median, 25th and 75th percentiles and outlying countries as individual data points

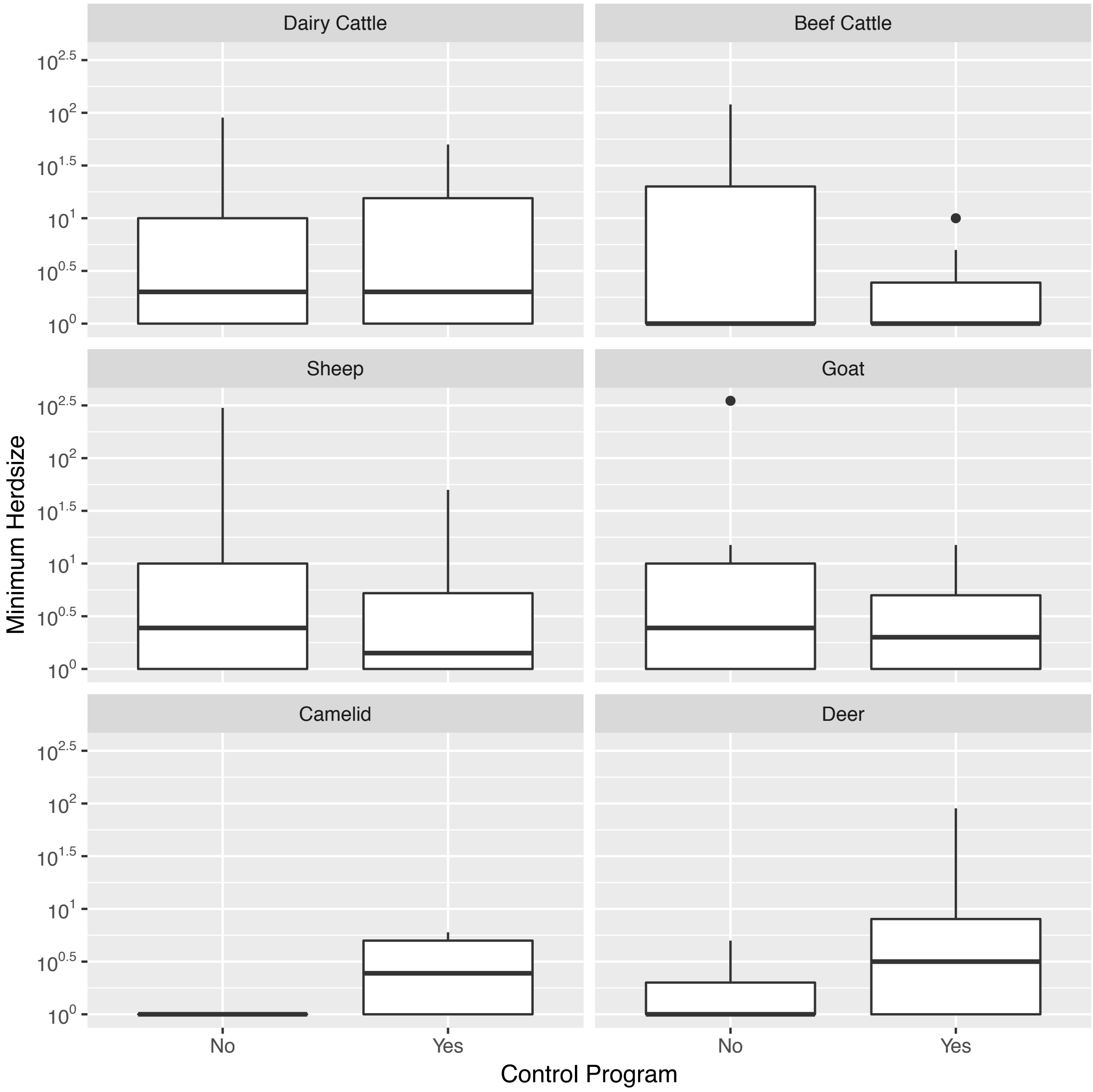

Supplementary Figure 4. Maximum herd size among 48 countries, 26 without a control program for paratuberculosis and 22 with a control program for paratuberculosis. The box represents the median, 25th and 75th percentiles and outlying countries as individual data points

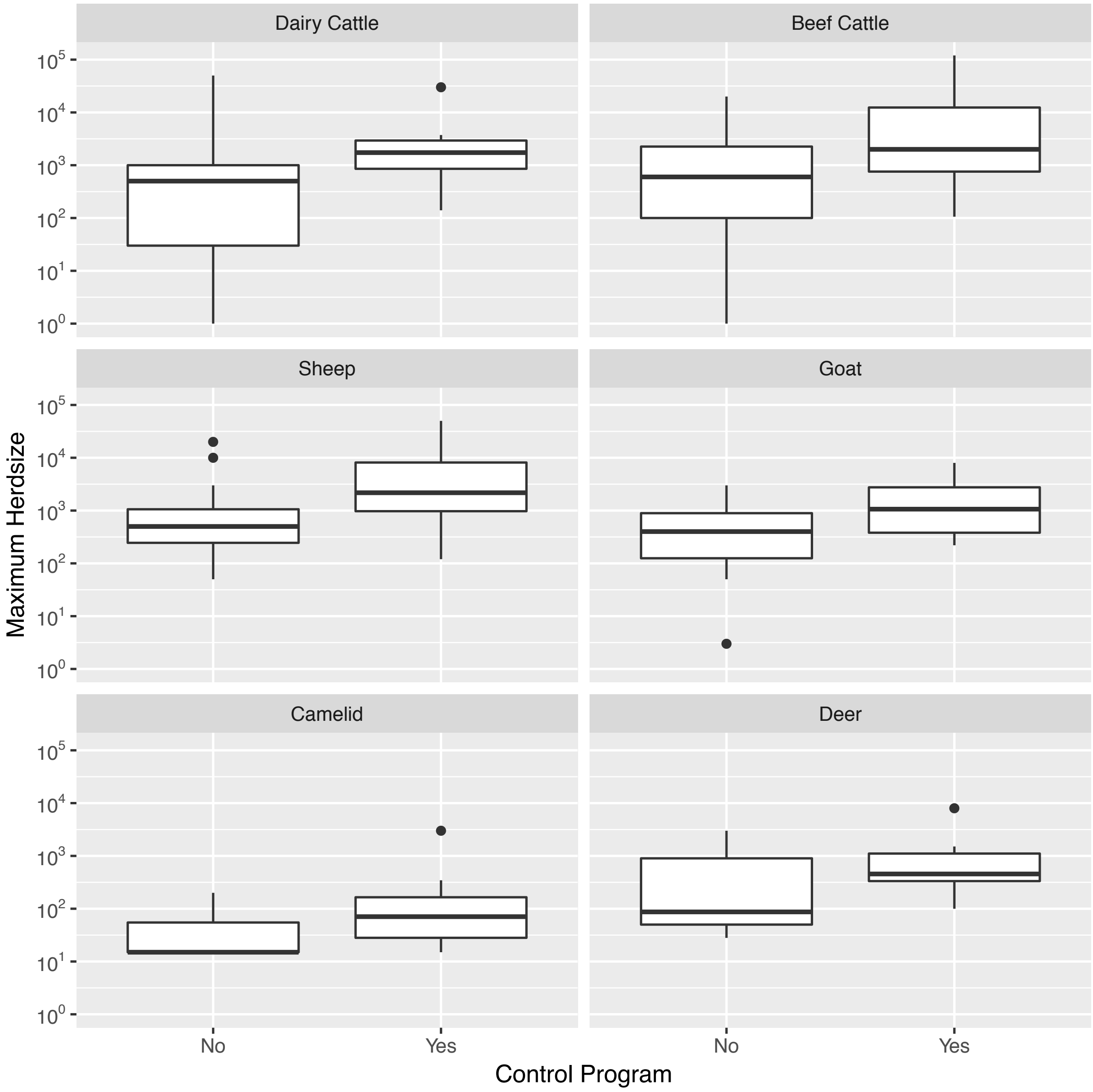

Supplement: Supplementary file 3 — Fig. S25-S2-S19 final. Data plots. (PDF 102 kb) [file 12917_2019_1943_MOESM3_ESM.pdf]
