# Supplementary material for: Control of paratuberculosis: who, why and how. A review of 48 countries
Source: BMC Vet Res. 2019 Jun 13;15:198. doi: 10.1186/s12917-019-1943-4 (PMC6567393; doi:10.1186/s12917-019-1943-4)
Supplement: Supplementary file 4 — Country-specific summaries 25–2-19 Tables. (PDF 280 kb) [file 12917_2019_1943_MOESM4_ESM.pdf]

Country-specific summaries

Argentina 2
Australia 3
Bangladesh 4
Belgium 5
Brazil 6
Canada 7
Colombia 8
Czech Republic 9
Denmark 10
Ecuador 11
Finland 12
France 13
Germany 14
Greece 15
Iceland 16
India 17
Iran 18
Republic of Ireland 19
Italy 20
Korea 21
Mexico 22
The Netherlands 23
New Zealand 24
Nigeria 25
Norway 26
Panama 27
Poland 28
Slovenia 29
Spain 30
Sweden 31
United Kingdom 32
United States of America 33
Zambia 34

Argentina	
**Animal population**	There were 10,000,000 dairy and beef cattle, 10,000,000 adult sheep, 1,000,000 goats, 100,000 camelids, deer and south american camelids (www.senasa.org).
**Husbandry systems**	The system of dairy farming is intensive with two or three daily milkings in an approximate population of 250 adult cows per establishment. In beef cattle, the breeding system is extensive with the animals on pastures throughout the year. The dairy goats are found in farms with intensive systems but the meat goats are in very extensive conditions, and because of this the prevalence is very low compared to the dairy goats. The confinement of red deer makes the prevalence and impact very high.
**Paratuberculosis situation**	Endemic in dairy cattle and potentially also in beef cattle. Reported in sheep and dairy goats with confirmation by isolation of MAP. The isolation of MAP from commercial milk has been reported.
**Specific features of current control program**	Not applicable.
**Other**	Control of paratuberculosis is voluntary, without any help form the government. The recommendations for voluntary control in dairy cattle, beef cattle or goats are to: carry out analysis by ELISA or use of PPD avium as screening tests with culture of feces or milk, and to remove the positive animals or suspects depending on the prevalence; to perform culture of faeces and affected tissues collectyed from animals in critical stages of the disease.

Australia	
**Animal population**	There were 1.5 million dairy cattle, 23 million beef cattle, 70 million sheep, 5 million goats (mostly rangeland), 300,000 alpacas, as well as some farmed deer and camels but these were mostly wild (1 million camels, 1 million deer) (www.agriculture.gov.au/abares).
**Husbandry systems**	Predominantly extensive systems with 50,000 farms with beef cattle and 9,000 farms with dairy cattle. In 2017-2018 there were 2.8 million beef cattle finished in feedlots. Sheep were produced on 32,000 farms. Two million rangeland goats are harvested annually (www.mla.com.au/prices-markets/Trends-analysis/fast-facts/).
**Paratuberculosis situation**	Endemic in dairy cattle in south eastern states; low prevalence elsewhere. Very low prevalence in beef cattle. Endemic in sheep in the southern states, but clinical disease is uncommon due to widespread use of Gudair® vaccine. Endemic in farmed goats, mostly dairy. Paratuberculosis is rare in camelids and deer. Surveillance is now limited.
**Specific features of current control program**	In 2016, national management of paratuberculosis in cattle changed from regulated control to market-driven, where farmers undertake practices dependent on their market requirements. They can use individual property biosecurity plans, assurance scores for beef and dairy cattle, and a national Cattle Health Declaration to assess, promote and manage risks. Western Australia and Northern Territory continue regulatory protection for paratuberculosis in cattle. The National Ovine JD 2013-18 Management Plan finished on 30 June 2018. Paratuberculosis in sheep is now managed by the national sheep industries within a broader Sheep Health Project, with various tools for sheep farmers at farm level for paratuberculosis – a Market Assurance Program, national Sheep Health Declaration and vaccination in endemic regions. Farmers in low prevalence areas may choose to work cooperatively under regional biosecurity plans. Abattoir monitoring of sheep has been a successful surveillance tool to inform policies and management. The goat and alpaca industries each have a Market Assurance Program, currently under review.
**Other**	Paratuberculosis remains notifiable for all species in all states and territories of Australia. Australia has well-established structures for consultation and collaboration between governments, industries and supporting sectors in the development and implementation of animal health programs, primarily for emergency diseases but including paratuberculosis.

Bangladesh	
**Animal population**	Estimated animal population based on a survey in 2015-2016: 23.78 million cattle, 3.35 million sheep, 25.76 million goats, 1.47 million buffalo.
**Husbandry system**	Bangladesh is an agricultural country. Livestock play an important role in the economy of the rural people. Most farmers maintain livestock in their household areas on a subsistence basis. Since livestock, especially dairy cattle and beef cattle, are an important source of income, many hundreds of small, semi-intensive, dairy and beef cattle farms have been established in rural and semi-urban areas together with a few large scale, intensive private or government owned farming systems producing dairy, cattle, sheep, goats and buffalo.
**Paratuberculosis situation**	Unpublished reports are available suggesting that cattle suffer from paratbuerculosis across the country from time to time. Diagnosis is mostly based on clinical findings. Organized field surveys (active surveillance) are crucial to get an overview on the occurrence of paratuberculosis and its distribution and also to evaluate its impact on animal production and the economy.
**Specific feature of current control program**	Not applicable.
**Other**	Symptomatic treatment of suspected cases could be considered. The exact situation with respect to patabuberculosis is not well known. A detailed study needs to be carried out.

Belgium	
**Animal population**	There were 2.3 million cattle, of which approximately 0.5 million were dairy cows; 0.2 million sheep and 0.1 million goats (estimates based on annual population census).
**Husbandry systems**	There were 23,000 cattle herds of which 7,000 were dairy herds, 25,000 sheep flocks and 9,600 goat herds.
**Paratuberculosis situation**	Endemic in dairy cattle and most likely also in beef cattle, although less commonly reported. Very little information exists on the situation in other ruminant species or wildlife.
**Specific features of current control program**	National monitoring program: voluntary, in dairy cattle, farmer and industry-driven since 2006, aiming to reduce the prevalence in affected herds and safeguard public health and trade. Approximately 5,200 participants. Annual or bi-annual testing (ELISA milk or serum) of at least all lactating animals older than 30 months. Seropositive animals are culled.<br>Regional (Wallonia) control program: voluntary, in dairy and beef cattle, ARSIA driven since 2011, aiming to eliminate the disease in affected herds. Approximately 120 participants. Annual testing (ELISA milk or serum and PCR faeces) of all animals older than 24 months. Application of biosecurity measures and strong shedders are culled with priority.
**Other**	

Brazil	
**Animal population**	There were 171,858,168 cattle, 8,254,561 goats;, 13,770,906 sheep and 948,103 buffalo (https://censos.ibge.gov.br/agro/2017/templates/censo_agro/resultadosagro/pecuaria.html).
**Husbandry systems**	There were 2,521,249 cattle farms, 326,166 goat farms 511,768 sheep farms and 14,728 buffalo farms. Beef cattle breeding focuses more on large farms, usually with more than 1000 hectares, while dairy cattle are more on small and medium-sized farms. Almost half the land on farms is allocated for pasture. (https://censos.ibge.gov.br/agro/2017/templates/censo_agro/resultadosagro/pecuaria.html).
**Paratuberculosis situation**	Disease present in multiple species, according to the Ministry of Agriculture, Livestock and Food Supply, health status of terrestrial animal diseases - Brazil, 2017. (http://www.agricultura.gov.br/assuntos/sanidade-animal-e-vegetable / animal health / archives-sisa / Animal_WAHID_Brazil_2017_02.pdf).
**Specific features of current control program**	Not applicable.
**Other**	Requires immediate notification of any confirmed case according to (a) Multiple species. List of diseases for compulsory notification to the Official Veterinary Service. (Regulatory Instruction no. 50, September 24, 2013).

Canada	
**Animal population**	Based on the 2016 Agricultural Census therew were 12.5 million cattle (adults and youngstock) of which 940,000 were dairy cows and 440,000 were replacement heifers, about 1 million sheep, 230,000 goats and 120,000 bison.
**Husbandry systems**	In 2017 there 10,900 dairy farms, 36,000 beef farms, 2,100 sheep farms and 860 goat farms. Dairy cattle are intensively housed in either free-stall (40% of farms) or tie-stall (60% of farms) housing, some of which may have seasonal access to pastures. Quebec and Ontario account for 2/3 of the national dairy herd. Organic milk production represents about 3% of the national farms. Beef cattle are either cow-calf operations or feedlots, with the majority of larger operations in western Canada. Sheep farms are primarily located in Ontario and Quebec (~50%) and contain mainly meat sheep. The majority of goat farms are dairy and reside in Ontario (~40%).
**Paratuberculosis situation**	Endemic in cattle, sheep and goats. It is a notifiable disease in most Canadian provinces. Estimated herd-level prevalence in dairy herds nationally is about 50%. There are no national estimates for beef cattle, but it is believed to be lower. While there are no national paratuberculosis levels for small ruminants, a provincial study of the dairy sheep and goat industries in Ontario estimated the flock- and herd-level true prevalence to be 66.8% and 83.0% respectively. In Québec, a study of cull sheep detected an animal prevalence of 3%.
**Specific features of current control program**	The only control programs are for dairy cattle. Voluntary control programs exist in each of Canada's four major regions, with some degree of national coordination. They are primarily farmer funded programs, with some degree of government support in the early stages. About 60% of herds nationally have participated in the programs to some degree. While there are differences among the regions, they are generally comprised of four major elements: education of farmers and vets; testing (method varies by region); on-farm risk assessment by a veterinarian; applied research. No control program exists for small ruminants.
**Other**	

Colombia	
**Animal population**	There were 26,367,814 cattle (female 17,338,570; male 9,029,244), 1,578,684 sheep, 1,000,132 goats and 336,417 buffalo (for 2018, Colombian Agrarian Institute, ICA www.ica.gov.co).
**Husbandry systems**	Cattle production is very diverse. There are 599,953 cattle herds and 2,319 buffalo herds nationally. There are several types of production systems, ranging from intensive beef, dairy and dual-purpose production in some regions of the country to extensive in others. Several *Bos taurus*, *Bos indicus* and Colombian creole breeds are used for beef, dairy, and dual-purpose production. Multiple cattle production systems coexist in different agro-ecological zones, with variable degrees of intensification, and are located in socio-economic environments of very diverse nature. The production of meat and milk is basically based on extensive beef production, low-tropic dairy production, and on dual-purpose systems, which comprise about 80.5 % of the total national livestock inventory, and contribute 50 and 70 % of milk and meat for the domestic market, respectively.
**Paratuberculosis situation**	Endemic. Paratuberculosis was first reported in cattle in 1924. Since then, the disease has been diagnosed mainly in cattle (specialized dairy herds) and sheep. Very few occurrences in goats, beef cattle, dual-purpose cattle or buffalo. Studies have focused primarily on agent detection and local prevalence estimations. Epidemiological information in the country is still scarce.
**Specific features of current control program**	Not applicable.
**Other**	Compared to cattle, the populations of sheep, goats and buffalo are small and therefore paratuberculosis has been less well investigated, and control efforts in the future would probably take much longer than in cattle. The disease has been notifiable only since 2015.

Czech Republic	
**Animal population**	There were 1,421,000 cattle of which 373,000 were dairy, 211,000 beef cattle, 217,000 adult sheep, 28,000 goats. There were also wild animals: 10, 369 red deer; 7,782 fallow deer, 98,574 row deer and 5,285 mouflon.
**Husbandry systems**	Cattle were bred in 18,786 farms (about 10,000 farms have 1-10 animals); dairy cattle in 3,500 farms; the majority of dairy cattle are bred indoor. Sheep were bred in 17,700 farms (about 12,000 farms have 1-10 animals). Goats were bred in 7,300 farms (about 6,400 farms have 1-10 animals).
**Paratuberculosis situation**	Assumed herd-level prevalence in cattle is higher than 40% (SVA test of 1,000 bulk tank milk samples in 2006). There are herds completely free of paratuberculosis and herds with up to 10% within-herd prevalence. However, detailed systematic information is lacking. MAP infection is assumed in sheep and goats based on research on milk samples in 2016 in which ELISA was positive. There is high incidence of paratuberculosis in wild ruminants based on past research projects (Machackova et al., 2004; Kopecna et al., 2008) and private testing by some owners of cattle/beef pastures. There is awareness of animal breeders of pasture contamination and risk of infection of domestic ruminants from wildlife sources.
**Specific features of current control program**	Not applicable.
**Other**	Since 2018 the Ministry of Agriculture has supported research on a certification programme for dairy cattle. Until 2017, (based on legislation), farmers had to report the occurrence of paratuberculosis in their herds to SVA. Nevertheless, there was no obligation for farmers to test animals for paratuberculosis. In the case of positive herds, restrictions (announcement of an outbreak, restrictions for import, export, milk production) were applied. For this reason, farmers deliberately avoided testing for paratuberculosis.

Denmark	
**Animal population**	There were 1.3 million female cattle (of which 570,000 were dairy cows) and 0.22 million male cattle, 71,000 adult sheep, 20,000 goats (2017, Statistics Denmark, www.dst.dk).
**Husbandry systems**	Cattle were from about 2,800 dairy herds and 10,000 beef herds. The latter were mostly small-holders who raised cattle as a hobby, there being only 500-1000 herds practicing commercial beef production. Sheep were from 6,700 flocks, goats were from 3,100 herds (Central Herd register, www.chr.fvst.dk), and as such, very few commercial goat and sheep production systems exist. The majority of cattle are kept indoors, except organic dairy cows (n~450) which have to have access to pasture for a minimum 150 days annually.
**Paratuberculosis situation**	Endemic in dairy cattle and potentially also in beef cattle, although less commonly reported. Has never been reported in sheep and goats, but no systematic testing has been carried out at least in the past 20 years.
**Specific features of control program**	A voluntary industry-driven paratuberculosis program has been in place in dairy herds since 2006 aiming to reduce the prevalence in affected herds. The programme is risk-based with frequent testing to increase the diagnostic sensitivity, where transmission from 1-time positive animals must be reduced via management-related features and repeated test-positive animals are recommended to be culled. No control programmes exist for non-dairy cattle, although a similar setup is recommended in affected beef herds if required.
**Other**	Because paratuberculosis has never been reported in sheep, goats or other ruminants, and because of their minor importance, no control activities are related to these species.

Ecuador	
**Animal population**	There were 4.13 million cattle of which 896,000 were dairy cows, 478,000 sheep and 36,000 thousand goats. (2016, INEC, www.ecuadorencifras.com).
**Husbandry systems**	The cattle are kept indoors. Of the 4.13 million cattle, 19.2% were for meat production, 11.8% for dairy production and 69% for both (2016, INEC, www.ecuadorencifras.com).
**Paratuberculosis situation**	Endemic in dairy cattle according to preliminary studies, but the national situation is unknown. Notification of paratuberculosis is limited. There are no reports of paratuberculosis in other animal species.
**Specific features of current control program**	Not applicable.
**Other**	Only some industries seek to control the disease with annual serological tests (ELISA), marking of positives and separation of the calves.

Finland	
**Animal population**	In 2017 there were 480,000 dairy cattle, 307,000 beef cattle, 144,000 sheep, 7,800 goats, 300 farmed deer, 193,000 reindeer.
**Husbandry systems**	In 2017 cattle were from about 7,800 dairy (average herd size 61) and 3,800 beef (average herd size 81) farms; sheep were from 3,900 farms; goats were from 960 farms; farmed deer were from 23 farms and reindeer from 4,400 establishments. The number of camelids (llama and alpaca) is not known but they are from 168 farms. Most of the dairy cattle and a part of the beef cattle farming is intensive.
**Paratuberculosis situation**	Since the early 1900s, paratuberculosis was recorded for the first time in 1992. In total it has been detected in five beef herds only, between 1992–2000. Since then paratuberculosis has not been found in beef cattle. It has never been detected in any other production animal or wildlife host and has never been found in dairy cattle.
**Specific features of current control program**	Not applicable.
**Other**	

France	
**Animal population**	There were 9 million dairy cattle, 10 million beef cattle, 1.3 million sheep and 7 million goats (http://idele.fr/).
**Husbandry systems**	Cattle are from about 193,000 herds, sheep are from about 4,900 flocks while goats are from 41,000 herds (http://idele.fr/).
**Paratuberculosis situation**	Paratuberculosis is present at varying levels in regions and herds. At national level, the prevalence of paratuberculosis is not precisely determined.
**Specific features of current control program**	Two main actions have been in place for cattle herds, on a voluntary basis:<br>- Control plan in infected herds with clinical cases<br>- Certification for herds with a very low level of risk
**Other**	

Germany	
**Animal population**	There were 4.2 million dairy cows and an equal number of dairy youngstock, about 3 million beef cattle, 1.5 million sheep, 35,000 dairy goats and other small goat and deer populations.
**Husbandry systems**	One of the most intensive dairy industries with mainly indoor free stalls and some pastoral systems in the north. Large variation of herd size with an average of about 300 cows in the eastern part and 40 cows in the southern part. About 275,000 beef cattle herds (>10 cattle) with extensive grazing, a high variation in herd size and a high proportion of small herds. About 9,900 sheep flocks, mainly extensive grazing (wide range of flock size, mostly 50- 500 sheep). About 300 commercial goat farms and numerous small hobby holdings.
**Paratuberculosis situation**	Endemic in pastoral based dairy cattle systems in the northern and western part and in 30-50% of the large dairy farms in the eastern part; low herd level prevalence in the southern part, low prevalence in beef herds; unknown prevalence in sheep, goats and deer with relevant underreporting.
**Specific features of control program**	Paratuberculosis is notifiable with no regulation at the federal level. Federal recommendations for voluntary control at farm level were published in 2014; key features: control stage with 3 levels (1. basic hygiene management, 2. identification of high-risk animals, 3. identification and removal of shedders), certification stage (3 years) and monitoring of MAP-non-suspect herds. This was adopted by 7 federal states as regional control programmes tailored according to the local production systems, prevalence situation and infrastructure for testing and counselling of farmers. Participation and compliance varies among regions. There is mandatory control in one federal state (Lower Saxony) based on serological testing, trade regulations for serologically positive cows and risk assessment. There is no systematic control in sheep and goat flocks. Vaccination is only possible with permission of the veterinary authority. In most voluntary programmes the animal industries carry both the responsibility and the risk, partly supported by the regional animal disease funds which are based on contributions paid by farmers.
**Other**	Commercially available diagnostic test kits have to be evaluated and licensed by the Federal Research institute of Animal Health (Friedrich-Loeffler-Institute). Research is focussed on the demands of the regional control programmes and is mainly funded by regional animal disease funds. There are no market incentives for milk and only to a small extend for breeding stock. Milk processors and their organisations encourage but do not subsidize the control efforts.

Greece	
**Animal population**	In 2016 there were 553,805 cattle of which 117,971 were dairy cattle; 8,738,618 dairy sheep; 3,887,902 dairy goats (Greek Statistical Authority).
**Husbandry systems**	The majority of cattle are kept indoors with the exception of a few organic dairy herds. Dairy sheep and goats are kept under semi-intensive management, while few flocks are reared intensively. The animals graze on communal pastures throughout most of the year and are additionally fed concentrates. They spend most of the day outside and are moved into the shed during the night. Greece has the largest goat herd in the EU. A plethora of traditional cheese products, which are of protected destination of origin (e.g. feta) or protected geographical indication, depend on the production of sheep and goat milk.
**Paratuberculosis situation**	Paratuberculosis is endemic in dairy cattle, sheep and goats. The majority of sheep and goat flocks are infected. A formal control scheme does not exist. Efforts to control paratuberculosis are voluntary, at the farm/flock level and aim to eliminate clinical cases and associated production losses. The most popular control option is vaccination of affected herds/flocks followed by annual vaccination of replacements. Fewer herds/flocks, often the ones that are severely affected, may select whole herd testing and removal of positive animals as a first step. Subsequently, vaccination of the remaining herd/flock followed by annual vaccination of replacements may follow.
**Specific features of current control program**	Not applicable.
**Other**	Sheep and goats: communal grazing and between-flock contact and trade of animals hampers attempts to control MAP infection at the flock level. Under the existing semi-intensive management system, control efforts should be at least at the regional level.

Iceland

**Animal population**	There were 26,742 dairy cattle, 24,654 beef cattle, 376,944 adult sheep and 1,300 goats.
**Husbandry systems**	Dairy cattle were from 619 herds, beef cattle from 772 herds, sheep from 2372 flocks and goats from 113 herds. The herd size was generally moderate. The average number of animals on farms was 43 dairy cows, 32 beef cattle, 159 sheep and 12 goats. The animals graze/go outdoors in the summer. In the winter most animals are housed and fed indoors.
**Paratuberculosis situation**	MAP sheep strain is the endemic strain in Iceland, infecting sheep, cattle and goats. Sheep farms have mainly been affected as the bacterial strain is more pathogenic in sheep. In recent years, outbreaks on sheep farms have been very rare.
**Specific features of current control program**	Paratuberculosis in sheep is kept under control by vaccination. The country is divided into areas depending on the paratuberculosis situation and the movement of animals and hay from endemic to paratuberculosis free areas is prohibited. Paratuberculosis in cattle is rare and spread of infection from sheep to cattle is less likely because mixed farming is gradually disappearing.
**Other**	

India	
**Animal population**	There were 187 million cattle, 63 million sheep, 133 million goats and 110 million buffalo.
**Husbandry systems**	Traditionally based on extensive husbandry. However, human population growth (1.33 billion) and limited land resources have led to intensification of livestock production systems over the last 25 years. 75% of large and 65% of small ruminants are under intensive production and commercial goat farming has emerged as a new animal industry.
**Paratuberculosis situation**	Endemic in domestic livestock. Based on surveys conducted between 1985 and 2017 the prevalence was 43% in cattle, 41% in sheep, 23% in goats and 36% in buffalo. In wild ruminants (deer, bison, blue bulls) and other animals (elephants, monkeys, rabbits, hyena, cats) it was 19% (microscopy) to 32% (culture). Prevalence of MAP in soil and water samples was high. MAP was also commonly detected in raw milk of individual animals, commercial milk samples, pooled milk samples and fresh cheese (paneer), infant milk powder, butter and ice cream using a range of methods. Mass screening of human samples from 2008 to 2017 showed 31% prevalence. Molecular epidemiology (2004-2017) in domestic livestock, wild ruminants, wild carnivores, monkeys, milk and milk products, human beings and the environment confirmed that 'Indian Bison Type' was the most prevalent (97%) strain of MAP, followed by 'Cattle type' MAP (3%).
**Specific features of current control program**	Not applicable.
**Other**	A test and slaughter approach from 1979 to 2004 did not reduce the prevalence in goats at the Central Institute for Research on Goats, Mathura (Uttar Pradesh). An 'Indigenous vaccine' was developed in 2005 using an 'Indian Bison type' of MAP. It was effective in cattle, sheep, goats and buffalo and was licensed for commercial production. Diagnostic tests, including approaches to differentiate infected and vaccinated animals (DIVA) were developed. The country has the necessary technical information and technologies to undertake a national control program, but it is awaiting government initiative.

Iran	
**Animal population**	There were 1,373,791 dairy and beef cattle, 65,600 domestic cattle, 18,500,600 goats, 46,601,000 sheep, 183,900 camelids (including Dromedary & Bactrian camels), 215,220 buffalo (Statistical Center of Iran, 2017; www.amar.org.ir).
**Husbandry systems**	There were 17,132 dairy herds, 8,929 beef herds. Nearly all cattle are kept indoors. (Statistical Center of Iran, 2017; www.amar.org.ir). Very few commercial intensive goat and sheep production systems exist, most animals being kept under traditional husbandry In which they are moved from one place to another by tribal herdspeople. During the temperate seasons, most of them graze on pastures. But during the cold seasons, they are fed manually indoors. There are different sheep breeds such as Afshari and Sangsari in different provinces, according to climate conditions. Camelids and buffaloes are also kept in some provinces, according to climatic conditions.
**Paratuberculosis situation**	Paratuberculosis is endemic in Iran. However, sheep and goats are affected much more than cattle. Testing is voluntary.
**Specific features of current control program**	Not applicable.
**Other**	Currently, there is no control program for paratuberculosis in Iran. But, regarding the high economic losses observed in sheep and goats, a control program is needed in the near future.

Republic of Ireland	
**Animal population**	In 2016-2017 there were 6.67 million dairy and beef catle of which 1.34 million were dairy cows, 4.66 million were female cattle and 2.01 million were male cattle. There were 3.9 million sheep and 7,800 goats (https://www.cso.ie).
**Husbandry systems**	Dairy production generally operates on a seasonal grass-based system with cattle housed for the winter months (early Dec – end March). During spring/summer and early autumn, supplements may be fed to address nutritional shortfalls in the pasture base and to match production levels. While housed, animals are fed a diet based on silage/concentrates. Beef production systems generally are pasture-based with animals housed during the winter months.
**Paratuberculosis situation**	Endemic in dairy and beef cattle. Reported in sheep and goats by private veterinary practitioners, but there has been no systematic testing undertaken and prevalence data are not available for small ruminants.
**Specific features of current control program**	From January 2019 Phase Two of the Irish Johne's Control Programme is to be based on:<br>1. Veterinary risk assessment and management plans (VRAMPs)<br>2. Whole herd testing (WHT)<br>3. Ancillary testing<br>4. Calculation of a Herd Assurance Score (HAS) based on test results and movement history<br>5. Bulk tank milk testing on a bi-annual basis for the purpose of national case finding (most heavily infected herds).
**Other**	A four-year programme is proposed from January 2019, to be based on cost-sharing between government, the milk processing sector and farmers.

Italy	
**Animal population**	There were 5.5 million cattle (2.6 million dairy cows, 2.9 million beef cattle), 6.9 million adult sheep, 1 million goats. Also about 397,000 water buffalo are present, mainly in the southern part of the country (Animal National Database for 2017 - http://statistiche.izs.it/portal/page?_pageid=73,12918&_dad=portal&_schema=PORTAL).
**Husbandry systems**	Cattle are from about 28,000 dairy herds and 128,000 beef herds. The majority of dairy cattle are kept indoors in intensive farms. Sheep are from 91,000 flocks and goats are from 53,000 flocks, mainly extensively reared. Water buffalo are from about 3,000 farms, mainly reared outdoor. (Animal National Database for 2017 - http://statistiche.izs.it/portal/page?_pageid=73,12918&_dad=portal&_schema=PORTAL).
**Paratuberculosis situation**	Paratuberculosis is widespread in Italy, where over 50% of bovine dairy herds are infected. In order to improve the health status of dairy herds and to protect the dairy export market, the Italian Ministry of Health issued in 2013 the "National guidelines for the control of bovine paratuberculosis and for assigning the health ranking of herds". The surveys that have been carried out in sheep and goat report similar prevalence estimates. The infection is present in wild ruminants, in particular deer.
**Specific features of current control program**	The program has been initially industry-driven and is managed through the collaboration of public veterinary services, bovine practitioners and the network of public laboratories (Istituti Zoooprofilattici Sperimentali).<br>The main components of the national guidelines are:<br>1. A passive surveillance system with mandatory reporting of clinically affected cows to the Veterinary Services.<br>2. A classification of bovine herds based on the risk of MAP infection in the herd, based on yearly individual ELISA test. There are seven MAP risk levels; the first two (PTC, PT0) are assigned by the national veterinary health services on the basis of presence (PTC) or absence (PT0) of clinical cases. The PTC level identifies those herds that, having had a clinical case, are not allowed to sell milk destined for dairy product exports. The accreditation for the other levels (PT1 to PT5) is obtained upon specific request of the farmer. The health status of the herd is based on results of standardized serological testing schemes voluntarily applied (S1 and S2 protocols).<br>3. The voluntary adoption of herd control programs, aimed at gradually reducing within-herd prevalence by adopting biosecurity measures and an appropriate testing scheme.
**Other**	The Guidelines have been applied to cattle and water buffalo herds. At the moment no activities are related to goats, sheep or wildlife.

Korea	
**Animal population**	In 2018 there were 0.4 million dairy cows, 3 million Korean native cattle (HANWOO) and 0.14 million beef cattle, about 0.2 million Korean native goats (Black goat) and less than 1,000 sheep and other goats (Statistics Korea, www.kostat.go.kr/).
**Husbandry systems**	Dairy cattle are from 6,522 dairy farms. Korean native cattle are from 92,581 farms and there are 7,346 beef cattle farms. Farm sizes are variable, ranging from 10 to more than 1,000 head. The majority of cattle are kept indoors. Korean native goats are free living in mountainous areas.
**Paratuberculosis situation**	Endemic in cattle farms, especially long-established farms. Between 400-500 positive cases per year are reported. Most of them are serologically positive. Serological testing based on ELISA is carried out on 20,000-30,000 head of cattle each year. No systematic testing of sheep, Koren black goats or other goats is undertaken.
**Specific features of current control program**	Paratuberculosis is a legal communicable disease in Korea. Several legal restrictions such as movement control etc. are applied to positive cases, both serologically detected and clinical. The positive animals are recommended to be culled. This system is mainly applicable to dairy cattle.
**Other**	Several wildlife species and black goats might be reservoirs of paratuberculosis in Korea.

Mexico	
**Animal population**	There were 2.5 million dairy cattle and 31 million beef cattle, 8.7 million sheep, 8.7 million goats. There are several species of deer in the wild and in hunting grounds, but their population sizes are not known. https://www.gob.mx/siap/documentos/poblacion-ganadera
**Husbandry systems**	Predominantly intensive systems for dairy cattle (50.6%), while 21% are semi-intensive, 18.3% are dual purpose (dairy and beef) and 9.8% are family systems. Beef cattle are mainly in extensive sytems with some finished in feedlots. In 2018, there were 120,000 fighting bulls at 33 cattle ranches nationwide. https://www.gob.mx/siap/documentos/poblacion-ganadera<br>Goats are in extensive systems (90%) with a small proportion in intensive systems (10%); only 5% of the total population belong to breeds specialized in dairy production and 95% are creole goats or crosses for dual purpose (meat and milk).<br>Sheep are in extensive and intensive systems. Two million sheep are harvested per year. There is an important tradition of consumption of sheep meat, but only 70% of the national demand is produced domestically http://www.anetif.org/files/pages/0000000034/20-produccion-de-carne-ovina.pdf.
**Paratuberculosis situation**	There are no recent studies on the paratuberculosis situation, but it has been detected in the different ruminant species. It was diagnosed ~30 years ago in bullfighting herds and vaccination was used for control at the initiative of owners and veterinarians. However, vaccine is not currently available because it interfers with the intradermal test used in the bovine tuberculosis campaign. In 2005 the economic impact was estimated in dairy cattle at $ 916 USD/per cow; the largest effect was due to the decrease in milk production http://132.248.9.34/ptb2005/01674/0350789/0350789.pdf.<br>There are insufficient diagnostic laboratories to develop diagnostic tests for paratuberculosis, and most producers are not willing to pay for testing because they believe the cost is high. Paratuberculosis control is carried out on some farms with their own financing.
**Specific features of current control program**	There is no campaign against this disease and no compulsory health program for herds or flocks.
**Other**	There is an interest in the disease only in some livestock sectors. In general, in the milk production system in cattle there is no interest in knowing the paratuberculoisis situation,even though there is participation in compulsory sanitary campaigns (e.g. tuberculosis and brucellosis).

The Netherlands	
**Animal population**	There were 3.9 million cattle, 1.3 million sheep and 0.6 million goats.
**Husbandry systems**	Cattle are kept in 16,600 dairy herds (average 102 adults) and 18,000 non-dairy herds (including dairy heifer rearing units, suckler herds, veal calf herds and bull fattening herds). Sheep are kept in 29,600 flocks of which 30% are large flocks (average 138 head) and 70% small flocks (average 7 head). Dairy goats are kept in 350 herds (average 1,200 head). (https://www.gddiergezondheid.nl/diergezondheid/monitoring). Seasonal grazing for ≥120 days is applied by approx. 75% of the dairy cattle herds. Young stock of 13% of the dairy herds are reared in specialised rearing units. Sheep are grazed for most of the year whereas dairy goats are mainly housed indoors in deep litter straw pens.
**Paratuberculosis situation**	MAP infection is endemic in dairy cattle, beef cattle and goats and has occasionally been observed in sheep. MAP control programs have been run from 1942 onwards, with a focus on test-and-cull (1942 -1984), vaccination (1984-1994), the combination of test-and-cull and preventive management measures in infected herds (1998 onwards) and surveillance of unsuspected herds (1998 onwards). From 2011 onwards, dairy cattle farmers are required by their milk processors to obtain a preferred herd status in a control programme, meaning that test-positive cattle have to be culled. Dairy goats are commonly vaccinated.
**Specific features of current control program**	Present programmes are: 1. The Intensive Paratuberculosis Program, initiated in 1998 and aiming at the certification-and-surveillance of unsuspected herds as a source of replacement cattle as well as elimination of MAP from known infected herds. 2. A milk quality assurance programme (MQAP), also known as Paratuberculose Programma Nederland (PPN), initiated in 2006 and aiming to reduce the concentration of MAP in milk delivered to the milk factories. 3. A control programme for sheep and goats, initiated in 2014 and aiming to reduce the MAP prevalence and the incidence of clinical paratuberculosis and provide assurance about herd/flock status.
**Other**	

New Zealand	
**Animal population**	There were 6.4 million dairy cattle, 3.7 million beef cattle, 31 million sheep, 1.1 millin deer, 66,000 dairy goats, and 20,000 alpaca.
**Husbandry systems**	Livestock are kept on 12,000 dairy farms, 17,000 beef farms, 13,000 sheep farms, 92 dairy goats farms, 1,100 deer farms and an unknown number of lifestyle blocks with alpaca. All species are kept in all-year pasture grazed, primarily seasonal calving/lambing systems. Lactating dairy cattle (450/herd) are supplemented with bailage but almost no concentrates. Many dairy farmers move calves, heifers and/or cows for off-farm grazing/rearing. Following artificial insemination, dairy and beef bulls enter dairy herds for natural mating (4-6 weeks), and dairy born 50% beef calves are moved to beef finishing farms. Beef cattle graze together with sheep for about 3 months around calving/lambing, covering a period of high MAP-susceptibility. Deer are grazed alone or together with sheep and/or beef cattle.
**Paratuberculosis situation**	There are no data for dairy cattle. About 42% of beef herds, 76% of sheep flocks and 59% of deer herds are infected with MAP, with annual clinical disease incidence of <1%. Due to frequent contacts between sheep and beef cattle, 80% of MAP infected beef cattle are carrying ovine type-I MAP and are developing clinical JD much less frequently than deer, dairy cattle or sheep. Sharing pasture reduces the within-species contact rate due to low species specific stocking density, hence co-grazed sheep and beef cattle appear to have lower infection prevalence and lower clinical JD incidence.
**Specific features of current control program**	All control schemes are voluntary. A JD control guide is available to be used by veterinarians for dairy clients. No control recommendations are generally practised by beef farmers. Vaccines are used by sheep farmers, mainly fine wool breeders. A systematic control programme exists for deer.
**Other**	The national goal for paratuberculosis control is to reduce production losses in domestic livestock. A multi-centre research programme was implemented during 2008 – 2016 (JDRC). Research has since subsided and currently relies on small-project funding at Massey and Otago universities.

Nigeria	
**Animal population**	There were 19.5 million cattle, 72.5 million sheep, 41.3 million goats (http://fmard.gov.ng/retreat-on-livestock-and-dairy-development-in-nigeria-keynote-address-delivered-by-the-hon-minister-of-agriculture-and-rural-development-chief-audu-ogbeh).
**Husbandry systems**	Extensive. In most instances, goats and sheep are reared together and in some instances reared with cattle. Dairy cattle are also reared together with beef animals in most traditional settings.
**Paratuberculosis situation**	No information is available on paratuberculosis in Nigeria. This is because it is not one of the diseases of economic or public health importance in the country. Again, little or no research work is currently being carried out on paratuberculosis when compared with bovine tuberculosis, which gradually gaining research interest.
**Specific features of current control program**	Not applicable.
**Other**	As a result of poor government funding and policy coupled with the fact that about 95% of cattle and other ruminants are owned by Fulani pastoralists who are mostly uneducated, animal health/welfare is still very rudimentary in Nigeria. Hence, veterinary care and support are limited, leading to an array of livestock diseases including paratuberculosis.

Norway	
**Animal population**	There were approximately 220,000 dairy cattle, 90,000 beef cattle, 1 million adult sheep, 80,000 goats, of which 35,000 are dairy goats, 2,000 llamas and alpaca and 700,000 wild, semi-domesticated and farmed cervids.
**Husbandry systems**	There are approximately 8,000 dairy cattle herds, 6,000 beef cattle herds, 14,000 sheep flocks (all for meat production), 300 dairy goat herds and 1,200 other goat herds. All are mainly indoor housing. Legislation requires that all cattle except bulls kept for slaughter have access to pasture for $\geq$ 2 months during summer. All goat herds and sheep flocks graze on fields or rangeland pastures for 3 to 5 months during summer.
**Paratuberculosis situation**	There are currently no known herds infected with paratuberculosis. Historically there have been few cases in cattle; the last registered case was in 2015. All cases in cattle after 1996 have been related to the importation of cattle, or infection from goats. Six cases have been reported in sheep, all related to infected goat herds or imported cattle; the last registered case was in 2007. The infection was not reported in camelids in Norway until 2014, when two cases in alpaca were registered. Paratuberculosis was endemic in goats before 2001, particularly in western parts of Norway. A large eradication programme in goats was active from 2001 to 2014. There was one registered case in goats in 2015.
**Specific features of current control program**	In the surveillance and control programme, fecal samples from 100 cattle herds, 120 goat herds, 30 sheep flocks and approx. 150 holdings with camelids are investigated for MAP each year. Participation is compulsory, and sampling and analysis is paid by the authorities. In addition, the industry analyses bulk milk samples from dairy goats five times a year. Paratuberculosis is notifiable. Suspected animals are sampled. Vaccination has been prohibited since 2015. Restrictions on trade and movement of animals apply on suspicion or diagnosis. Stamping out (or sanitation in goat herds) is compulsory upon diagnosis.
**Other**	The disease eradication programme ”Healthier goats” in endemic areas was based on sanitizing goat herds. Goat kids were removed at birth before contact with the environment, fed paratuberculosis free cows’ milk and reared separately from their dams. This is labour intensive and demanding to perform, however it has proven to be an efficient tool to eradicate the infection from goat herds. The programme has been a success, as there have been no positive tested dairy goats in sanitized herds since 2010.

Panama	
**Animal population**	There were approximately 1,521,500 cattle. This includes more than 210,400 cattle in dairy farms. Sheep and goat population sizes have not been estimated yet.
**Husbandry systems**	Both intensive and extensive husbandry systems are used by both the dairy and beef cattle industries. These systems include a total of 43,858 herds. Nearly 40,000 herds are dedicated to beef cattle.
**Paratuberculosis situation**	An outbreak of paratuberculosis was reported in 2013 in one dairy cattle herd during a scientific research program. Nearly 25% of animals were positive in a commercial serological test. No clinical signs were observed but visible gross intestinal lesions and histopathological findings were compatible with paratuberculosis infection. MAP was not recovered by culture.
**Specific features of current control program**	Not applicable.
**Other**	Currently, there is no surveillance or control program for paratuberculosis. Paratuberculosis vaccination has not been implemented yet. Since trade in live cattle is frequent within Panama and with neighbouring countries, a national survey of paratuberculosis remains key to determine the true prevalence of the disease. Also, studies to determine the economical impact in the dairy industry are required. Finally, scientific research is required to determine the effect of paratuberculosis and its prevalence on the diagnosis of bovine tuberculosis in Panama.

Poland	
**Animal population**	There were 6,035,700 cattle (including 2,340,700 dairy cows), 268,500 sheep and 82,000 goats (https://stat.gov.pl).
**Husbandry systems**	Cattle breeding is very fragmented among 382,700 dairy cattle herds, 295,000 of which raise only one to ten cows. The number of farms with more than 100 dairy cows is around 2,060. There are about 1,000 beef herds with an average of 24 head (www.nowypm.pl).
**Paratuberculosis situation**	Endemic in dairy cows and potentially also in beef cattle and goats. Paratuberculosis has never been reported in sheep.
**Specific features of current control program**	Not applicable.
**Other**	Until now, cases of paratuberculosis have been reported only in large dairy large herds with over 100 cows.

Slovenia

**Animal population**	There were 480,000 cattle of which 200,000 were dairy cows, 120,000 adult sheep, 30,000 goats, 1,500 deer and 150 camelids.
**Husbandry systems**	Cattle are from 5050 dairy herds (average 40 cattle per herd, range 1 to 580) and 25,000 mostly beef herds (around 11 animals per herd, range 1 to 780). Cattle are often bred indoors, but usually most of them have access to pastures. The time of access depends on the territory (Alpine, Mediterranean or Pannonian). Goats and sheep are mostly in Alpine and Mediterranean parts of country. Goats are from 3,840 herds (range 1 to 290 animals). Sheep are from 5340 flocks (range 1 to 720 animals). Deer are in 124 herds and camelids are bred on 35 farms.
**Paratuberculosis situation**	Endemic in dairy cattle, the most often in Holstein Friesians. There is also high prevalence in Limousine cattle but much less in other beef cattle breeds. Paratuberculosis is also found in goats, being a very serious problem in some breeds, and also in sheep. Paratuberculosis is a notifiable disease in cattle, sheep and goats and is excluded from artificial insemination centres.
**Specific features of current control program**	Not applicable.
**Other**	In November 2018 a project will commence: “Rising the competitive position of Slovenian cattle farming by controlling paratuberculosis in dairy cattle herds” financed by Ministry of Agriculture, Forestry and Food and the Slovenian Research Agency. The main goal of the project is to establish a paratuberculosis control program.

Spain	
**Animal population**	There were 5,537,800 cattle, 29,150,787 sheep, 6,438,511 goats, 16,223 farmed deer, 580 camels and 300 South American camelids.
**Husbandry systems**	Average herd sizes were 75 for intensive dairy cattle, 59 for extensive beef cattle, 175 for extensive bullfighting cattle,<br>790 for intensive dairy sheep, 480 for extensive dairy sheep,<br>179 for extensive meat sheep, 232 for intensive dairy goats, 130 for extensive dairy goats, 90 for extensive meat goats, 20 for camels (tourism), 11 for other camelids (pets) and 32 for deer.
**Paratuberculosis situation**	Endemic in dairy cattle and some dairy goat and sheep regions; rare in beef and meat sheep; common in bullfighting farms.
**Specific features of control program**	Control programs are voluntary. They are linked to subsidies through local farmer associations. For cattle, detection is based mostly serological testing and fecal confirmatory PCR, with culling of positives being the recommendation. For sheep and goats, there is routine vaccination of replacement stock in affected regions/farms.
**Other**	

Sweden	
**Animal population**	In 2017 there were 1.5 million cattle including 322,010 dairy cows and 207,620 suckler cows in meat production, and 301,468 adult sheep. Goats were not systematically counted but were estimates to be <20,000 (Agricultural Statistics 2018, www.jordbruksverket.se).
**Husbandry systems**	In 2017 there were about 16,700 holdings with cattle; 3,600 of these had dairy cattle. Legislation requires that all cattle (except bulls kept for slaughter) have access to pasture during the summer. The number of holdings with sheep and goats were 9,300 and 2,500, respectively; the majority were small-scale enterprises (Surveillance of infectious diseases in animals and humans in Sweden 2017, www.sva.se).
**Paratuberculosis situation**	Due to limited livestock trade, legislation and systematic eradication campaigns during the 20th century, Sweden has a unique situation where prevalence is very low, or not present at all. Sporadic cases have previously occurred in beef cattle, all of them connected directly or indirectly to imported animals. In accordance with legislation (since 1952; Swedish Act of Epizootic diseases, SFS 1999:657), detection of cases has been followed by whole herd stamping-out, tracing and sanitation measures, with the goal to eradicate the disease and to prevent spread. Vaccination is prohibited by law and notification is mandatory on clinical suspicion. MAP has not been detected in dairy cattle, other ruminant species or wildlife. Importation of livestock to Sweden has been very limited and based on an agreement among Swedish dairy farmers, no dairy cattle have been imported since Sweden joined the EU in 1995. (Surveillance of infectious diseases in animals and humans in Sweden 2017, www.sva.se).
**Specific features of current control program**	Surveillance of paratuberculosis in Sweden is currently based on three main components: 1. Passive surveillance based on mandatory clinical suspicion 2. Enhanced passive surveillance based on sampling of all adult ruminants submitted for post mortem examination, and 3. Active surveillance based on the voluntary surveillance program. The program includes all main beef breeding herds and some dairy herds selling calves to beef herds within the program. Program herd status is based on repeated faecal sampling and control of livestock trade. The program also includes awareness campaigns directed to farmers and staff performing meat inspection at abattoirs.
**Other**	

United Kingdom	
**Animal population**	In 2017 there were 9,787,000 cattle and calves of which 1,539,000 were adult beef cattle and 1,904,000 were adult dairy cattle. In addition there were 23,310,000 sheep and lambs (Defra statistics https://www.gov.uk/government/statistical-data-sets/structure-of-the-livestock-industry-in-england-at-december#history).
**Husbandry systems**	The majority of cattle are extensively grazed during spring/summer and housed during autumn/winter; the practice can vary depending on weather, availability of feed, stage of lactation. A small number of herds may be out wintered or in all-year housing. There is a stratified system for sheep: hill, upland and lowland extensive grazing. Co- or sequential grazing of cattle and sheep is common practice.
**Paratuberculosis situation**	Endemic in cattle and sheep. Prevalence survey data are available only for the national dairy herd in 2006-2007 (http://archive.defra.gov.uk/foodfarm/farmanimal/diseases/atoz/johnes/index.htm). Notifiable for cattle, sheep and goats in Northern Ireland but not in Great Britain.
**Specific features of current control program**	A number of voluntary paratuberculosis control programs for cattle have been in place since 1998. These are regulated by Cattle Health Certification Standards UK (CHeCS). The current Johne's Disease Risk Level Certification Program (beef and dairy) aims to provide an assessment of the risk of paratuberculosis being present in the herd, to reduce the risk of paratuberculosis within the herd and to allow the marketing of cattle with an accredited risk level. The Johne's Disease Risk Level Reduction Programme (dairy) aims to reduce the detrimental effects on herd productivity caused by paratuberculosis and to reduce disease prevalence over time with the long term goal to achieve freedom from disease, but the removal of test positive animals is not a strict requirement. Additionally, the voluntary industry-led National Johne's disease management plan was launched in 2015 to manage and then reduce the incidence of paratuberculosis in dairy cattle and engage 95% of dairy farmers in Great Britain in credible and robust disease management activities. There are six options for control strategies with different goals to suit different herd situations (http://www.actionjohnesuk.org/control-strategies/).
**Other**	There are currently no control programmes for sheep and goats in UK and uptake of the Gudair vaccine is low. Vaccination of cattle is not recommended due to interference with statutory skin testing for bovine tuberculosis.

United States of America	
**Animal population**	There were 9 million dairy cattle, 89 million beef cattle, 5 million sheep and 2 million goats (https://www.nass.usda.gov).
**Husbandry systems**	Mainly intensive dry lot and loose housing based dairy farming (herd size average about 144), extensive grazing of beef cattle (herd size average about 98), sheep (flock size average about 60), and goats (herd size average about 98).
**Paratuberculosis situation**	National studies conducted by United States Department of Agriculture (USDA) Animal and Plant Health Inspection Service, Veterinary Services, National Animal Health Monitoring System (NAHMS) estimate a herd prevalence for paratuberculosis of 68.1 percent for the dairy industry, with herds that had greater than 500 animals with an estimated herd prevalence of 95 percent (NAHMS Dairy 2002), and at 7.9 percent for the beef industry (NAHMS Beef 1997). At the time of these studies 31.7% of the dairy producers participated in a paratuberculosis control program of some kind and 35.3% tested for paratuberculosis in their herds. More recent national studies have not been completed. A national estimate has not been determined for other livestock species although paratuberculosis is known to exist.
**Specific features of control program**	Paratuberculosis controls in livestock species is mainly a voluntary effort in the USA. Movement of livestock species is only restricted in cases of animals confirmed to be infected using an official organism detection test (i.e. culture or PCR in an approved laboratory). Paratuberculosis is not reportable nationally although some states still have regulations requiring positive cases to be reported. Control programs are mostly voluntary and based on risk assessment and a herd management plan developed between the producer and a private veterinarian. An organized program does not exist for the sheep and goat industries although individual states will assist producers that reach out to them. The main role of federal and state governments is outreach, education and laboratory testing.
**Other**	USDA maintains approved laboratory testing through its National Veterinary Services Laboratories ensuring state and private laboratories are proficient.

Zambia	
**Animal population**	There were 4,984,909 cattle, 890,000 sheep and 2.1 million goats.
**Husbandry systems**	83 % of cattle farming is under traditional extensive production, with less than 17% being under intensive commercial production.
**Paratuberculosis situation**	This is a neglected disease. Although on paper it is a notifiable disease, there are no control mechanisms in place or efforts to test herds. However, based on information from large animal practitioners, paratuberculosis is endemic in both extensive and intensive animal production.
**Specific features of current control program**	Not applicable.
**Other**	Cases of paratuberculosis in cattle are recorded routinely at the University of Zambia's veterinary post-mortem and pathology laboratories, especially in exotic dairy breeds.
