# Supplementary material for: Control of paratuberculosis: who, why and how. A review of 48 countries
Source: BMC Vet Res. 2019 Jun 13;15:198. doi: 10.1186/s12917-019-1943-4 (PMC6567393; doi:10.1186/s12917-019-1943-4)
Supplement: Supplementary file 5 — Table S15 ver 25–2-19 final. (DOCX 43 kb) [file 12917_2019_1943_MOESM5_ESM.docx]

Table S15. Roles of authors

| **Name** | **Discussed and agreed the scope of the study** | **Designed or pilot tested the questionnaire** | **Collected, managed, analysed data** | **Attended a meeting to discuss the results** | **Wrote sections of the paper** | **Wrote a country-specific summary** | **Critically reviewed drafts of the paper** |
| --- | --- | --- | --- | --- | --- | --- | --- |
| Richard Whittington | Y | Y | Y | Y | Y | Y | Y |
| Karsten Donat | Y | Y | Y | Y | Y | Y | Y |
| Maarten F.Weber | Y | Y | Y | Y | Y | Y | Y |
| David Kelton | Y |  | Y | Y | Y | Y | Y |
| Søren Saxmose Nielsen | Y | Y | Y |  | Y | Y | Y |
| Suzanne Eisenberg | Y |  | Y | Y | Y | Y | Y |
| Norma Arrigoni | Y |  | Y | Y | Y | Y | Y |
| Ramon Juste | Y |  | Y | Y | Y | Y | Y |
| Jose Luis Sáez | Y | Y | Y |  | Y | Y | Y |
| Navneet Dhand | Y | Y | Y |  | Y |  | Y |
| Annalisa Santi | Y |  | Y |  | Y | Y | Y |
| Anita Michel | Y |  | Y |  |  |  | Y |
| Herman Barkema | Y | Y | Y | Y |  | Y | Y |
| Petr Kralik | Y | Y | Y | Y |  | Y | Y |
| Polychronis Kostoulas | Y |  | Y |  | Y |  | Y |
| Lorna Citer | Y | Y | Y | Y |  | Y | Y |
| Frank Griffin | Y | Y | Y | Y |  | Y | Y |
| Rob Barwell | Y |  | Y | Y |  | Y | Y |
| Maria Aparecida Scatamburlo Moreira | Y |  | Y | Y |  | Y | Y |
| Iva Slana | Y |  | Y | Y |  | Y | Y |
| Heike Koehler | Y |  | Y | Y |  | Y | Y |
| Shoor Vir Singh | Y |  | Y | Y |  | Y | Y |
| Han Sang Yoo | Y |  | Y | Y |  | Y | Y |
| Gilberto Gris | Y |  | Y | Y |  | Y | Y |
| Amador Goodridge | Y |  | Y | Y |  | Y | Y |
| Matjaz Ocepek | Y |  | Y | Y |  | Y | Y |
| Joseba Garrido | Y |  | Y | Y |  | Y | Y |
| Karen Stevenson | Y |  | Y | Y |  | Y | Y |
| Mike Collins | Y |  | Y | Y |  | Y | Y |
| Bernardo Alonso | Y |  | Y |  |  | Y | Y |
| Karina Cirone | Y |  | Y |  |  | Y | Y |
| Fernando Paolicchi | Y |  | Y |  |  | Y | Y |
| Lawrence Gavey | Y |  | Y |  |  | Y | Y |
| Md Tanvir Rahman | Y |  | Y |  |  | Y | Y |
| Emmanuelle de Marchin | Y |  | Y |  |  | Y | Y |
| Willem Van Praet | Y |  | Y |  |  | Y | Y |
| Cathy Bauman | Y |  | Y |  |  | Y | Y |
| Gilles Fecteau | Y |  | Y |  |  | Y | Y |
| Shawn McKenna | Y |  | Y |  |  | Y | Y |
| Miguel Salgado | Y |  | Y | Y |  |  | Y |
| Jorge Fernández-Silva | Y |  | Y | Y |  |  | Y |
| Radka Dziedzinska | Y |  | Y |  |  | Y | Y |
| Gustavo Echeverría | Y |  | Y |  |  | Y | Y |
| Jaana Seppänen | Y |  | Y |  |  | Y | Y |
| Virginie Thibault | Y |  | Y |  |  | Y | Y |
| Vala Fridriksdottir | Y |  | Y |  |  | Y | Y |
| Abdolah Derakhshandeh | Y |  | Y |  |  | Y | Y |
| Masoud Haghkhah | Y |  | Y |  |  | Y | Y |
| Luigi Ruocco | Y |  | Y |  |  | Y | Y |
| Satoko Kawaji | Y |  | Y | Y |  |  | Y |
| Eiichi Momotani | Y |  | Y | Y |  |  | Y |
| Cord Heuer | Y |  | Y |  |  | Y | Y |
| Solis Norton | Y |  | Y |  |  | Y | Y |
| Simeon Cadmus | Y |  | Y |  |  | Y | Y |
| Angelika Agdestein | Y |  | Y |  |  | Y | Y |
| Annette Kampen | Y |  | Y |  |  | Y | Y |
| Joanna Szteyn | Y |  | Y |  |  | Y | Y |
| Jenny Frössling | Y |  | Y |  |  | Y | Y |
| Ebba Schwan | Y |  | Y |  |  | Y | Y |
| George Caldow | Y |  | Y |  |  | Y | Y |
| Sam Strain | Y |  | Y |  |  | Y | Y |
| Mike Carter | Y |  | Y |  |  | Y | Y |
| Scott Wells | Y |  | Y |  |  | Y | Y |
| Musso Munyeme | Y |  | Y |  |  | Y | Y |
| Robert Wolf | Y |  | Y |  |  |  | Y |
| Ratna Gurung | Y |  | Y |  |  |  | Y |
| Cristobal Verdugo | Y |  | Y |  |  |  | Y |
| Christine Fourichon | Y |  | Y |  |  |  | Y |
| Takehisa Yamamoto | Y |  | Y |  |  |  | Y |
| Sharada Thapaliya | Y |  | Y |  |  |  | Y |
| Elena Di Labio | Y |  | Y |  |  |  | Y |
| Monaya Ekgatat | Y |  | Y |  |  |  | Y |
| Andres Gil | Y |  | Y |  |  |  | Y |
| Alvaro Nuñez | Y |  | Y |  |  |  | Y |
| José Piaggio | Y |  | Y |  |  |  | Y |
| Alejandra Suanes | Y |  | Y |  |  |  | Y |
| Jacobus H. de Waard | Y |  | Y |  |  |  | Y |
